# 2-Allylphenyl glycosides as complementary building blocks for oligosaccharide and glycoconjugate synthesis

**DOI:** 10.3762/bjoc.8.66

**Published:** 2012-04-18

**Authors:** Hemali D Premathilake, Alexei V Demchenko

**Affiliations:** 1 Department of Chemistry and Biochemistry, University of Missouri – St. Louis, One University Boulevard, St. Louis, MO 63121, USA

**Keywords:** carbohydrates, glycosylation, leaving group, oligosaccharides, orthogonal strategy, selective activation

## Abstract

The *O-*allylphenyl (AP) anomeric moiety was investigated as a new leaving group that can be activated for chemical glycosylation under a variety of conditions, through both direct and remote pathways. Differentiation between the two activation pathways was achieved in a mechanistic study. The orthogonal-type activation of the AP moiety along with common thioglycosides allows for the execution of efficient oligosaccharide assembly.

## Introduction

**Scheme 1:**
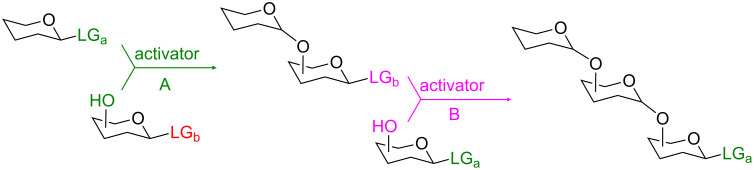
Orthogonal strategy introduced by Ogawa et al.

Current knowledge about the key roles of carbohydrates is still limited. However, thanks to the explosive growth of the field of glycobiology in recent years, we have already learned that carbohydrates are involved in a broad range of vital biological processes (e.g., fertilization, anti-inflammation, immunoresponse, joint lubrication, antigenic determination) [1]. Carbohydrates are also involved in many harmful processes (e.g., bacterial and viral infections, development of tumors, metastasis, tissue rejection, congenital disorders). The fact that many of these processes are directly associated with the pathogenesis of deadly diseases, including AIDS, cancer, pneumonia, septicemia, hepatitis and malaria [2–4], has been particularly stimulating for major scientific efforts in the field of modern glycosciences. The traditional chemical synthesis of oligosaccharides is lengthy because it involves multiple manipulations of protecting and/or leaving groups between glycosylation steps [5]. Many advanced strategies that shorten the oligosaccharide assembly by minimizing or even eliminating additional manipulations between coupling steps, are based either on chemoselective or on selective activation of leaving groups [6]. The use of selective activation [7] offers more flexibility than that of the chemoselective activation which relies on the nature of the protecting groups [8], and Ogawa’s orthogonal strategy is conceptually the most attractive approach that has been developed to date [9–12]. This technique implies the use of two orthogonal leaving groups, LG_a_ and LG_b_, the selective activation of which can be reiterated to give streamlined access to oligosaccharides (Scheme 1).

Yet, the orthogonal strategy remains underdeveloped, with too few examples to become universally applicable. Only Ogawa’s *S*-phenyl (SPh) versus fluoride [9–10,13] and our thioimidate-based approaches [14–16] are known. A very promising orthogonality was shown for *O-*pentenyl versus *O-*propargyl glycosides by Hotha et al. [17] and for *S-*glycosyl *O-*methyl phenylcarbamothioate (SNea) versus thioglycosides/thioimidates by us [18], but still their applicability to multistep synthesis remains to be proven. Working to expand this concept, our group reported a related, albeit less flexible, semiorthogonal approach with the use of *S-*ethyl and *O-*pentenyl leaving groups [19], which was later extended to fluoride/*O-*pentenyl combination by Fraser-Reid and Lopez [20]. Oxygen- [21] and sulfur-based [22] leaving groups fit into many expeditious strategies for oligosaccharide synthesis [6]. However, suitable reaction conditions for the orthogonal activation of these two classes of leaving groups are yet to be found. Commonly, *O-*glycosides are too stable to be used as effective glycosyl donors [21]. Pent-4-enyl *O-*glycosides introduced by Fraser-Reid are unique in this category of leaving groups, because they can be glycosidated under mild conditions by using I^+^ generated in situ. Also this method has its limitations since I^+^ can also activate thioglycosides. Indeed, only the semiorthogonality of the *O-*pentenyl and SEt leaving groups could be established [19]. Additionally, 4-pentenol is rather expensive ($ 323/50 g, Aldrich), and although *O-*pentenyl can be introduced from the anomeric acetate directly, the most economical synthesis includes a three-step protocol, with halide, orthoester, and the rearrangement of the latter to glycoside.

## Results and Discussion

As a part of the ongoing research effort to develop versatile building blocks, we present herein the development of a new *ortho*-allylphenyl (AP) leaving group. In line with other efforts [23–26], the AP group was specifically designed to address the drawbacks of *O-*pentenyl glycosides and to create a more flexible approach for oligosaccharide synthesis. Concomitantly with our studies, Hung et al. came up with essentially the same idea and reported the synthesis of AP mannosides and their activation for *O-*mannosylation in the presence of ICl/AgOTf [27].

The following considerations driving our efforts were of particular relevance. First, chemists have been making aryl glycosides for some 130 years [28], and many excellent protocols for their synthesis are available [29]. We determined that AP glycosides can be readily obtained from the corresponding peracetate by using inexpensive 2-allylphenol ($ 35/100 g, Aldrich) in the presence of BF_3_·Et_2_O. For instance, acetylated AP β-D-glucopyranoside was obtained in 92% yield. Second, we anticipated that the same promoters used for *O-*pentenyl activation [30] can also activate the AP leaving group. However, since AP glycosides bear structural features of both aryl and pentenyl glycosides, they should offer a more versatile activation profile than either class of the leaving group. Our working hypothesis is that activation of the AP leaving group with I^+^ takes place by the formation of an epi-iodonium ion, which is then opened with the anomeric oxygen, similarly to that known for *O-*pentenyl glycosides [30]. It is possible that the activation of the AP leaving group can also be achieved with TMSOTf or BF_3_·Et_2_O via the direct anomeric activation pathway, which was expected to become the key feature of the AP-mediated glycosylation approach in comparison to that of both *O-*pentenyl or thioglycosides. As a result, this pathway may offer a suitable platform for developing a fully orthogonal approach in combination with thioglycosides.

To pursue this methodology we obtained a range of differently protected AP glucosides, including perbenzylated **1a**, perbenzoylated **1b**, and derivative **1c** equipped with the superarming protecting-group pattern (2-*O-*benzoyl-3,4,6-tri-*O-*benzyl) [31]. For comparison, we also obtained the AP donor **1d** of the D-galacto series. With glycosyl donors **1a**–**d** in hand, we began evaluating their applicability to chemical glycosylation using a range of standard glycosyl acceptors **2**–**5** [18]. Encouragingly, the reaction of glycosyl donor **1a** with the primary glycosyl acceptor **2** in the presence of TMSOTf was completed within 15 min and provided the corresponding disaccharide **6a** in 82% yield (Table 1, entry 1). As expected, when a control experiment was set up with MeOTf, no glycosylation of **2** took place (Table 1, entry 2). The fact that the AP group in **1a** can be activated with TMSOTf, but not with MeOTf, offers a basis for exploring its orthogonality to thioglycosides. This is because thioglycosides show a completely opposite reactivity trend, namely no reaction with TMSOTf and smooth glycosylation with MeOTf [32].

NIS/TMSOTf is a powerful promoter for the activation of both *O-*pentenyl and thioglycosides. It was also found to be effective for the activation of AP glycosyl donor **1a**, upon which disaccharide **6a** was obtained in 80% yield. Even faster reaction and higher yield was obtained by using the NIS/TfOH promoter system, wherein the resulting disaccharide **6a** was obtained in 90% yield (Table 1, entry 4). The latter reaction conditions were chosen for use in the investigation of the glycosylation of the secondary glycosyl acceptors **3**–**5**.

**Table 1:** Glycosylation of AP glycosyl donors **1a**–**d**.

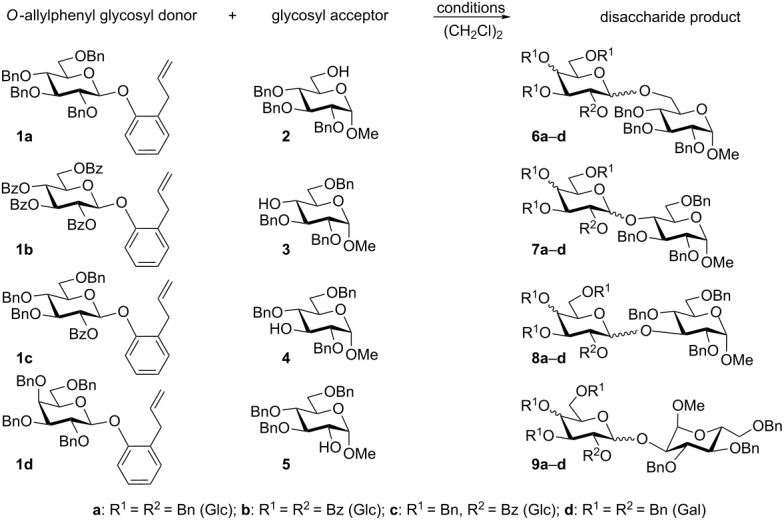				

entry	donor + acceptor	conditions^a^	time	product (yield, α/β ratio)

1	**1a** + **2**	TMSOTf, rt	15 min	**6a** (82%, 2.7/1)
2	**1a** + **2**	MeOTf, rt	24 h	no reaction
3	**1a** + **2**	NIS/TMSOTf, 0 °C	40 min	**6a** (80%, 1.4/1)
4	**1a** + **2**	NIS/TfOH, 0 °C	15 min	**6a** (90%, 1/1.5)
5	**1a** + **3**	NIS/TfOH, 0 °C	4 h	**7a** (73%, 1.2/1)
6	**1a** + **4**	NIS/TfOH, 0 °C	30 min	**8a** (82%, 1.0/1)
7	**1a** + **5**	NIS/TfOH, 0 °C	3 h	**9a** (72%, 1/1.3)
8	**1b** + **2**	MeOTf, rt	24 h	no reaction
9	**1b** + **2**	TMSOTf, rt	24 h	no reaction
10	**1b** + **2**	NIS/TMSOTf, 0 °C	6 h	**6b** (77%, β only)
11	**1b** + **2**	NIS/TfOH, 0 °C	2 h	**6b** (71%, β only)
12	**1b** + **5**	NIS/TfOH, 0 °C	3 h	**9b** (78%, β only)
13	**1c** + **2**	MeOTf, rt	24 h	no reaction
14	**1c** + **2**	TMSOTf, −20 °C	2.5 h	**6c** (73%, β only)
15	**1c** + **2**	NIS/TMSOTf, 0 °C	10 min	**6c** (84%, β only)
16	**1c** + **2**	NIS/TfOH, −20 °C	15 min	**6c** (93%, β only)
17	**1d** + **2**	NIS/TfOH, 0 °C	15 min	**6d** (85%, 1.5/1)
18	**1d** + **3**	NIS/TfOH, 0 °C	4 h	**7d** (81%, 1.2/1)
19	**1d** + **4**	NIS/TfOH, 0 °C	30 min	**8d** (82%, 1.0/1)
20	**1d** + **5**	NIS/TfOH, 0 °C	2 h	**9d** (80%, 3.0/1)

^a^performed in the presence of molecular sieves 4 Å (or 3 Å in MeOTf-promoted reactions, entries 2, 8, and 13).

**Scheme 2:**
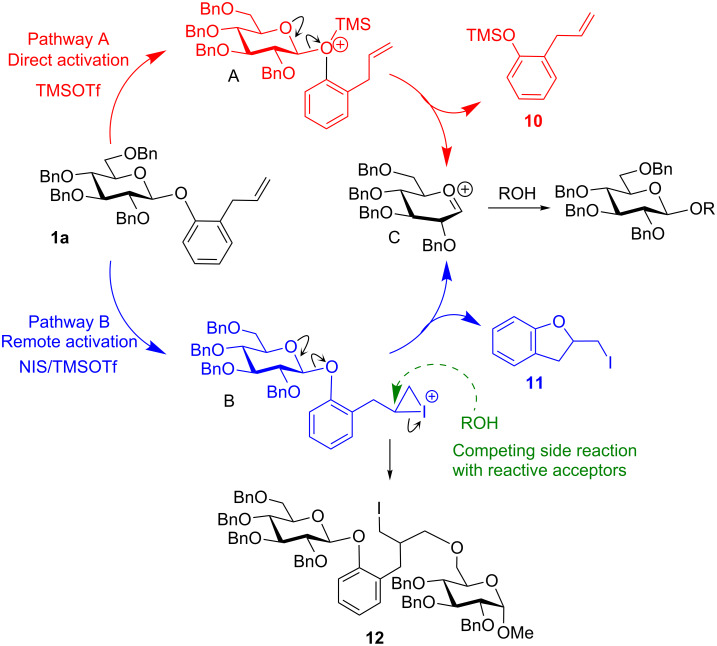
Determination of the AP activation pathways.

These couplings were also proven to be feasible, and the corresponding disaccharides **7a**–**9a** were obtained in 72–82% yield (Table 1, entries 5–7). As anticipated, the reactivity of the perbenzoylated (disarmed) counterpart **1b** was significantly lower than that of **1a**, and TMSOTf-promoted glycosylation of **1b** was practically ineffective (Table 1, entry 9). This observation along with the fact that the armed AP leaving group in **1a** can be readily activated with TMSOTf (Table 1, entry 1) suggests that AP glycosides can be applied in accordance with the classic armed–disarmed strategy [33]. NIS-promoted glycosylations of **1b** have proven to be of preparative value and the desired disaccharides **6b** and **9b** were isolated in 71–78% yield (Table 1, entries 10–12). In order to gain a more flexible activation profile for the synthesis of 1,2-trans glycosides, we also investigated AP glycosyl donor **1c**, bearing a superarming protecting-group pattern [31]. As expected, all of the previously established activation conditions were very effective, and glycosylation of **1c** with acceptor **2** readily produced disaccharide **6c** (Table 1, entries 14–16). Again, particularly efficient was the NIS/TfOH-promoted reaction wherein disaccharide **6c** was isolated in 93% yield (Table 1, entry 16). To broaden the scope of the AP approach, we tested its applicability to the synthesis of D-galactosides, which are a highly important and abundant sugar series. All glycosylations of AP donor **1d** proceeded smoothly in the presence of NIS/TfOH and the corresponding disaccharides **6d**–**9d** were obtained in 80–85% yield (Table 1, entries 17–20). To verify the two anticipated activation pathways by which the AP group may depart, all components of the key experiments (Table 1, entries 1 and 3) wherein glycosyl donor **1a** reacted with glycosyl acceptor **2** in the presence of TMSOTf and NIS/TMSOTf, respectively, were separated and analyzed. In the TMSOTf-promoted reaction, (*o*-allylphenoxy)trimethylsilane (**10**) was isolated and identified by comparison with analytical data obtained from a commercial sample. This result suggests that the activation of the AP moiety with TMSOTf takes place through the anomeric oxygen atom (**A**, Pathway A, Scheme 2). In the NIS/TMSOTf-promoted reaction, 2-iodomethyl-2,3-dihydrobenzofuran (**11**) [34] was isolated and its identity was proven by spectral methods indicating that activation with I^+^ takes place through the remote allyl moiety (**B**, Pathway B). In addition, in the latter experiment we detected the presence of adduct **12**, which was formed as the result of a competing attack of the glycosyl acceptor oxygen, as opposed to endocyclization of **B** through the anomeric oxygen leading to oxacarbenium intermediate **C**.

In accordance with Fraser-Reid’s armed–disarmed approach*,* electronically activated (armed) glycosyl donors are chemoselectively activated over deactivated (disarmed) glycosyl acceptors bearing the same type of leaving group [30,35]. To explore this avenue, we obtained a disarmed (benzoylated) 6-OH AP glycosyl acceptor **13** (Supporting Information File 1), which was coupled with the armed AP glycosyl donor **1a** in the presence of TMSOTf. As anticipated, this reaction was feasible and the expected disaccharide **14** was obtained in 78% yield (Table 2, entry 1). With the ultimate goal of developing distinct orthogonal differentiation of AP and thioglycosides, we next obtained glycosyl acceptor **15** [36] equipped with an ethylthio leaving group. TMSOTf-promoted chemoselective glycosylation between building blocks **1a** and **15** produced the expected disaccharide **16** in 71% yield (Table 2, entry 2).

**Table 2:** AP glycosides as glycosyl donors and acceptors in chemoselective and selective activations.

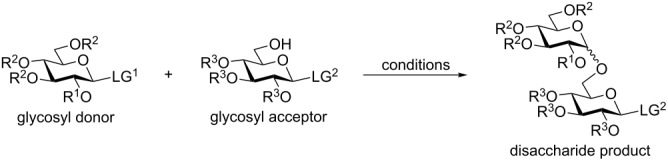				

entry	donor; acceptor	promoter^a^	time	product (yield, α/β ratio)

1	**1a** (LG^1^ = OAP, R^1^ = R^2^ = Bn); **13** (LG^2^ = OAP, R^3^ = Bz)	A	10 min	**14** (78%, 1.0/1)
2	**1a**; **15** (LG^2^ = SEt, R^3^ = Bn)	A	15 min	**16** (71%, 1.0/1)
3	**1a**; **17**(LG^2^ = STol, R^3^ = Bz)	A	1 h	**18** (75%, 2.4/1)
4	**1a**; **19** (LG^2^ = SPh, R^3^ = Bn)	A	1 h	**20** (90%, 1.0/1)
5	**1a**; **21** (LG^2^ = SPh, R^3^ = Bz)	A	2 h	**22** (98%, 1.8/1)
6	**1c** (LG^1^ = OAP, R^1^ = Bz, R^2^ = Bn); **21**	A	30 min	**23** (75%, β only)
7	**24** (LG^1^ = SEt, R^1^ = R^2^ = Bn); **25** (LG^2^ = OAP, R^3^ = Bn)	B	2 h	**26** (82%, 1.8/1)
8	**27** (LG^1^ = STaz, R^1^ = R^2^ = Bn); **25**	B	1 h	**26** (78%, 1.0/1)
9	**28** (LG^1^ = STol, R^1^ = R^2^ = Bn); **25**	B	4 h	**26** (97%, 1.2/1)
10	**29** (LG^1^ = SPh, R^1^ = R^2^ = Bn); **25**	B	6 h	**26** (90%, 1.0/1)

^a^performed in the presence of molecular sieves 4 Å (A: TMSOTf) or 3 Å (B: MeOTf) at rt or 0 °C (entry 6).

**Scheme 3:**
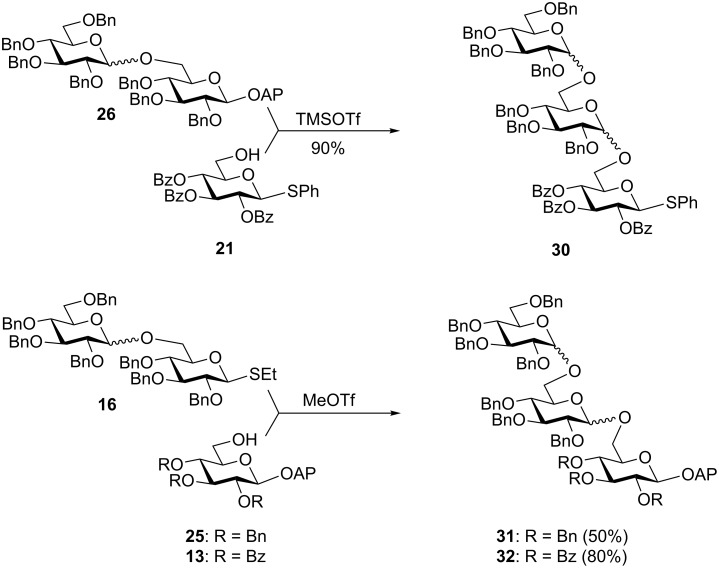
AP building blocks in oligosaccharide synthesis.

In a selective activation fashion, glycosyl acceptors **17** [37] and **19** [38], equipped with *S-*tolyl (STol) and *S-*phenyl leaving groups, respectively, were glycosylated with AP glycosyl donor **1a** to afford disaccharides **18** and **20** in 75 and 90% yield, respectively (Table 2, entries 3 and 4). Also the disarmed SPh acceptor **21** [39] led to disaccharide **22** in 98% yield. The synthesis of a 1,2-trans-linked disaccharide was also possible with glycosyl donor **1c** leading to the corresponding β-linked disaccharide **23** in 75% yield (Table 2, entry 6). This series of experiments clearly demonstrates that the AP leaving group can be reliably activated with TMSOTf in the presence of *S-*alkyl/aryl leaving groups. We next investigated glycosyl acceptor **25** equipped with the AP leaving group. MeOTf-promoted glycosylation of SEt, STaz, STol, and SPh glycosyl donors **24** [40], **27** [14], **28** [41], and **29** [42], respectively, with acceptor **25** afforded the respective disaccharide **26** in 78–97% yield (Table 2, entries 7–10). These series of results indicates a completely orthogonal character of AP and the thioglycosides. To expand this observation, disaccharide **26** was coupled with thioglycoside acceptor **21** in the presence of TMSOTf leading to trisaccharide **30** in 90% (Scheme 3). Since **30** is equipped with the SPh anomeric leaving group, it is available for further chain elongation directly. In a similar fashion, thioglycoside disaccharide **16** was coupled with AP acceptors **25** and **13** in the presence of MeOTf to afford trisaccharides **31** and **32** in 50 and 80% yield, respectively. Since these trisaccharides are equipped with the AP leaving group, their direct application to further chain elongation can be envisaged.

## Conclusion

In conclusion, we investigated the *O-*allylphenyl (AP) anomeric moiety as a new leaving group that can be activated for chemical glycosylation under a variety of conditions including Lewis acid and iodonium ion mediated pathways. The two activation pathways were confirmed by a mechanistic study. We also demonstrated that the application of the AP moiety allows executing oligosaccharide assembly by an orthogonal concept. The application of the AP glycosides may stretch well beyond their initial intended purpose because the alkene moiety can be utilized in a variety of other modes. Similar to that of *O-*pentenyl [30], it can be temporarily deactivated toward the I^+^ activation pathway by the addition of Br_2_, which can be reverted as needed (active–latent strategy). Direct conjugation of the AP moiety to biomolecules, monolayers, arrays, etc., should be possible by executing thiol–ene chemistry [43], ozonolysis/reductive amination [44–46], or other ligation protocols [47–48].

## Experimental

### General remarks

Column chromatography was performed on silica gel 60 (EM Science, 70–230 mesh), reactions were monitored by TLC on Kieselgel 60 F_254_ (EM Science). The compounds were detected by examination under UV light and by charring with 10% sulfuric acid in methanol. Solvents were removed under reduced pressure at <40 °C. CH_2_Cl_2_ and ClCH_2_CH_2_Cl were distilled from CaH_2_, directly prior to application. Anhydrous DMF (EM Science) was used as received. Methanol was dried by heating under reflux with magnesium methoxide, distilled and stored under argon. Pyridine and acetonitrile were dried by heating under reflux with CaH_2_ and then distilled and stored over molecular sieves (3 Å). Molecular sieves (3 Å or 4 Å), used for reactions, were crushed and activated in vacuo at 390 °C during 8 h in the first instance and then for 2–3 h at 390 °C directly prior to application. DOWEX MONOSPHERE 650C (H) was washed three times with MeOH and stored under MeOH. Optical rotations were measured with a Jasco P-1020 polarimeter. ^1^H NMR spectra were recorded in CDCl_3_ at 300 MHz; ^13^C NMR spectra were recorded in CDCl_3_ at 75 MHz (Bruker Avance) or 125 MHz (Varian). Anomeric ratios were determined by comparison of the integral intensities of the respective groups of signals in the ^1^H NMR spectra. HRMS determinations were made with the use of a JEOL MStation (JMS-700) mass spectrometer.

### Synthesis of glycosides

**Typical MeOTf-promoted glycosylation procedure (Method A)*.*** A mixture of glycosyl donor (0.11 mmol), glycosyl acceptor (0.10 mmol), and freshly activated molecular sieves (3 Å, 300 mg) in 1,2-dichloroethane (1.4 mL) was stirred under argon for 1 h. MeOTf (0.33 mmol) was added and the reaction mixture was monitored by TLC. Upon completion (Table 1, Table 2), the solid was filtered off and the residue was rinsed with CH_2_Cl_2_. The combined filtrate (30 mL) was washed with 20% aq NaHCO_3_ (10 mL) and water (3 × 10 mL). The organic layer was separated, dried with MgSO_4_ and concentrated in vacuo. The residue was purified by silica gel column chromatography (ethyl acetate/hexanes gradient elution) to afford the corresponding oligosaccharide.

**Typical TMSOTf-promoted glycosylation procedure (Method B)*.*** A mixture of glycosyl donor (0.11 mmol), glycosyl acceptor (0.10 mmol), and freshly activated molecular sieves (4 Å, 150 mg) in 1,2-dichloroethane (1.6 mL) was stirred under argon for 1 h. TMSOTf (0.22 mmol) was added and the reaction mixture was monitored by TLC. Upon completion (Table 1, Table 2), the solid was filtered off and the residue was rinsed with CH_2_Cl_2_. The combined filtrate (30 mL) was washed with 20% aq NaHCO_3_ (10 mL) and water (3 × 10 mL). The organic layer was separated, dried with MgSO_4_ and concentrated in vacuo. The residue was purified by silica gel column chromatography (ethyl acetate/hexanes gradient elution) to afford the corresponding oligosaccharide.

**Typical NIS/TfOH-promoted glycosylation procedure (Method C).** A mixture of glycosyl donor (0.11 mmol), glycosyl acceptor (0.10 mmol), and freshly activated molecular sieves (4 Å, 150 mg) in 1,2-dichloroethane (1.6 mL) was stirred under argon for 1 h. NIS (0.22 mmol) and TfOH (0.022 mmol) were added and the reaction mixture was monitored by TLC. Upon completion, the mixture was diluted with CH_2_Cl_2_, the solid was filtered off, and the residue was rinsed with CH_2_Cl_2_. The combined filtrate (30 mL) was washed with 10% aq Na_2_S_2_O_3_ (10 mL) and water (3 × 10 mL). The organic layer was separated, dried with MgSO_4_ and concentrated in vacuo. The residue was purified by silica gel column chromatography (ethyl acetate/hexanes gradient elution) to afford the corresponding oligosaccharide.

**Typical NIS/TMSOTf-promoted glycosylation procedure (Method D).** A mixture of glycosyl donor (0.11 mmol), glycosyl acceptor (0.10 mmol), and freshly activated molecular sieves (4 Å, 150 mg) in 1,2-dichloroethane (1.6 mL) was stirred under argon for 1 h. NIS (0.22 mmol) and TMSOTf (0.022 mmol) were added and the reaction mixture was monitored by TLC. Upon completion, the mixture was diluted with CH_2_Cl_2_, the solid was filtered off, and the residue was rinsed with CH_2_Cl_2_. The combined filtrate (30 mL) was washed with 10% aq Na_2_S_2_O_3_ (10 mL) and water (3 × 10 mL). The organic layer was separated, dried with MgSO_4_ and concentrated in vacuo. The residue was purified by silica gel column chromatography (ethyl acetate/hexanes gradient elution) to afford the corresponding oligosaccharide.

## Supporting Information

File 1 Experimental procedures, extended experimental data, ^1^H and ^13^C NMR spectra for all new compounds.
