# Supplementary material for: 2-Allylphenyl glycosides as complementary building blocks for oligosaccharide and glycoconjugate synthesis
Source: Beilstein J Org Chem. 2012 Apr 18;8:597–605. doi: 10.3762/bjoc.8.66 (PMC3343285; doi:10.3762/bjoc.8.66)
Supplement: File 1 — Experimental procedures, extended experimental data, 1H and 13C NMR spectra for all new compounds. [file Beilstein_J_Org_Chem-08-597-s001.pdf]

Supporting information

for

2-Allylphenyl glycosides as complementary building blocks for oligosaccharide and glycoconjugate synthesis

Hemali D. Premathilake and Alexei V. Demchenko*

Address: Department of Chemistry and Biochemistry, University of Missouri – St. Louis, One University Boulevard, St. Louis, MO 63121, USA

Email: Alexei V. Demchenko - demchenkoa@umsl.edu

*Corresponding author

Experimental procedures, extended experimental data,
^{1}H and ^{13}C NMR spectra for all new compounds.

Content

Synthesis of glycosyl donors S2
Synthesis of glycosyl acceptors S7
Synthesis of additional building blocks (SI only) S10
Data for di- and trisaccharides S14
NMR spectra S21
References S61

Synthesis of glycosyl donors

2-Allylphenyl 2,3,4,6-tetra-*O*-benzyl-β-D-glucopyranoside (1a). 2-Allylphenyl 2,3,4,6-tetra-*O*-acetyl-β-D-glucopyranoside (see below for the synthesis, 1.00 g, 2.16 mmol) was dissolved in methanol (8 mL), and the pH was adjusted (pH 9) by careful addition of a 1 M solution of $NaOCH_3$ in MeOH (~0.1 mL). The reaction mixture was kept for 1 h at rt, then Dowex (H^+) was added until neutral pH was reached. The resin was filtered off and rinsed with methanol (3 × 5 mL). The combined filtrate (~30 mL) was concentrated in vacuo and dried. The resultant solid was dissolved in DMF (14 mL) and benzyl bromide (1.41 mL, 11.82 mmol) was added. The resulting mixture was cooled to 0 °C and NaH (0.426 g, 17.74 mmol) was added portionwise. The reaction mixture was allowed to gradually warm to rt. After stirring for 1 h at rt, the reaction was quenched by stirring with ice water (50 mL). The organic phase was extracted with ethyl acetate/diethyl ether 1/1 (v/v, 3 × 40 mL) and the combined organic extract was washed with water (3 × 20 mL), the organic phase was separated, dried with $MgSO_4$ and concentrated in vacuo. The residue was purified by column chromatography on silica gel (ethyl acetate/hexane gradient elution) to afford the title compound (1.3 g, 92%) as white crystals. Analytical data for **1a**: R_f 0.50 (ethyl acetate/hexanes 1:5, v/v); mp 94–97 °C (diethyl ether/hexanes); $[\alpha]_D^{23}$ +16.7 (*c* 1.0, $CHCl_3$); ^1H NMR: δ 3.34–3.49 (m, 2H, $PhCH_2CH{=}CH_2$), 3.54–3.55 (m, 1H, H-5), 3.60–3.76 (m, 5H, H-2, H-3, H-4, H-6a, H-6b), 4.43–4.54 (m, 2H, CH_2Ph), 4.76–4.94 (m, 4H, 2×CH_2Ph), 4.94–5.00 (m, 4H, $J_{1,2}$ = 7.8 Hz, H-1, $PhCH_2CH{=}CH_2$, 1/2×CH_2Ph), 5.97–6.10 (m, 1H, $PhCH_2CH{=}CH_2$), 6.94–7.28 (m, 24H, aromatic) ppm; ^{13}C NMR: δ 34.3, 69.0, 73.7, 75.2, 75.3, 75.4, 76.0, 77.4, 78.0, 82.3, 85.1, 101.5, 115.6, 116.1, 122.8, 127.6, 127.7, 127.8, 127.9 (×4), 128.0 (×2),

128.1 (×2), 128.2 (×2), 128.5 (×2), 128.6 (×3), 128.7 (×2), 129.8, 130.1, 136.9, 138.2, 138.3, 138.4, 138.7, 155.1 ppm; HRMS–MS (*m*/*z*): $[M + Na]^+$ calcd for $C_{43}H_{44}O_6Na^+$, 679.3036; found, 679.3058.

2-Allylphenyl 2,3,4,6-tetra-*O*-benzoyl-β-D-glucopyranoside (1b). 2-Allylphenyl 2,3,4,6-tetra-*O*-acetyl-β-D-glucopyranoside (see below for the synthesis, 5.40 g, 11.63 mmol) was dissolved in methanol (44 mL), and the pH was adjusted to pH 9 by careful addition of 1 M solution of $NaOCH_3$ in MeOH (~0.2 mL). The reaction mixture was kept for 1 h at rt, then Dowex (H^+) was added until neutral pH. The resin was filtered off and washed with methanol (3 × 5 mL). The combined filtrate was concentrated in vacuo and dried. The residue was dissolved in dry pyridine (70 mL), the mixture was cooled to 0 °C and benzoyl chloride (7.2 mL, 62.5 mmol) was added dropwise. The reaction mixture was allowed to gradually warm to rt. After stirring for 1 h at rt, the reaction was quenched by the addition of methanol (5 mL). The resulting mixture was evaporated and coevaporated with toluene (3 × 10 mL) under reduced pressure. The residue was diluted with CH_2Cl_2 (20 mL) and washed with water (10 mL), sat. aq. $NaHCO_3$ (10 mL) and water (3 × 10 mL). The organic layer was separated, dried with $MgSO_4$, and concentrated in vacuo. The residue was purified by column chromatography on silica gel (ethyl acetate/hexane gradient elution) to afford the title compound (8.8 g, 90%) as white crystals. Analytical data for **1b**: R_f 0.58 (ethyl acetate/hexanes 4:10 v/v); mp 130–132 °C (diethyl ether/hexanes); $[\alpha]_D^{23}$ +26.7 (*c* 1, $CHCl_3$); ^1H NMR: δ 3.06 (d, 2H, J = 6.6 Hz, $PhCH_2CH{=}CH_2$), 4.18–4.26 (m, 1H, H-5), 4.39 (dd, 1H, $J_{6a,6b}$ = 12.0 Hz, $J_{5,6a}$ = 6.8 Hz, H-6a), 4.54–4.69 (m, 3H, H-6b, $PhCH_2CH{=}CH_2$), 5.26 (d, 1H, $J_{1,2}$ = 6.0 Hz, H-1), 5.50–5.64 (m, 2H, H-4,

$PhCH_2CH{=}CH_2$), 5.77 (dd, 1H, $J_{2,3}$ = 9.0 Hz, H-2), 5.90 (dd, 1H, $J_{3,4}$ = 9.5 Hz, H-3), 6.82–7.92 (m, 24H, aromatic) ppm; ^{13}C NMR: δ 34.0, 63.4, 69.9, 71.7, 72.8, 73.0, 99.9, 115.4, 115.7, 123.4, 128.5 (×2), 128.6 (×2), 128.7 (×3), 128.8 (×2), 128.9, 129.0, 129.3, 129.7, 129.8 (×3), 129.9 (×2), 130.0 (×2), 130.1 (×2), 130.2, 130.3, 133.4, 133.5, 133.8, 136.4, 154.8, 165.2, 165.5, 166.0, 166.2 ppm; HRMS–MS (*m*/*z*): $[M + Na]^+$ calcd for $C_{43}H_{36}O_{10}Na$, 735.2205; found, 735.2194.

2-Allylphenyl 2-*O*-benzoyl-3,4,6-tri-*O*-benzyl-β-D-glucopyranoside (1c). A mixture of 3,4,6-tri-*O*-benzyl-1,2-*O*-methoxybenzylidene-α-D-glucopyranose [1] (0.360 g, 0.63 mmol), molecular sieves (4 Å, 400 mg) and 2-allylphenol (0.84 mL, 6.34 mmol) in dry dichloromethane (3.6 mL) was stirred under argon for 10 min at rt. TMSOTf (0.03 mL, 0.16 mmol) was added, and the resulting mixture was stirred at rt for 4 h. After that, the reaction mixture was filtered through celite, the filtrate was diluted with dichloromethane (30 mL) and then washed with water (10 mL), sat. aq. $NaHCO_3$ (10 mL), and water (3 × 10 mL). The organic phase was separated, dried over $MgSO_4$, and concentrated in vacuo. The residue was purified by column chromatography on silica gel (ethyl acetate/hexane gradient elution) to afford the title compound (0.120 g, 28% yield) as a white solid. Analytical data for **1c**: R_f 0.59 (ethyl acetate/hexanes 3:10, v/v); $[\alpha]_D^{23}$ +26.4 (*c* 1, $CHCl_3$); ^1H NMR: δ 3.31 (d, 1H, *J* = 10.0 Hz, $PhCH_2CH{=}CH_2$), 3.85–4.06 (m, 5H, H-3, H-4, H-5, H-6a, H-6b), 4.71–5.01 (m, 8H, $PhCH_2CH{=}CH_2$, 3 × CH_2Ph), 5.21 (d, 1H, $J_{1,2}$ = 7.9 Hz, H-1), 5.76 (dd, 1H, $J_{2,3}$ = 9.0 Hz, H-2), 5.79–5.89 (m, 1H, $PhCH_2CH{=}CH_2$), 7.04–8.14 (m, 24H, aromatic) ppm; ^{13}C NMR: δ 33.98, 68.99, 73.6, 73.8, 75.3, 75.4, 75.7, 78.1, 82.9, 99.8, 115.2, 115.5, 122.9, 127.5, 127.8, 127.9, 128.0 (×2), 128.1, 128.2 (×4), 128.4 (×3), 128.5 (×2), 128.6 (×2), 128.7 (×2), 130.0 (×2), 130.1

(×2), 133.3, 136.7, 137.9, 138.0, 138.3, 155.2, 165.3 ppm; HRMS–MS (*m*/*z*): $[M + Na]^+$ calcd for $C_{43}H_{42}O_2Na$ 693.2831, found 693.2834.

2-Allylphenyl 2,3,4,6-tetra-*O*-benzyl-β-D-galactopyranoside (1d). 2-Allylphenyl 2,3,4,6-tetra-*O*-acetyl-β-D-galactopyranoside (see below for the synthesis, 2.00 g, 4.32 mmol) was dissolved in methanol (16 mL), and the pH was adjusted (pH 9) by careful addition of a 1 M solution of $NaOCH_3$ in MeOH (~0.1 mL). The reaction mixture was kept for 1 h at rt, then Dowex (H^+) was added until neutral pH was reached. The resin was filtered off and washed with methanol (3 × 10 mL). The combined filtrate was concentrated in vacuo and dried. The resultant solid was dissolved in dry DMF (24 mL) and benzyl bromide (2.4 mL, 20.27 mmol). Then the reaction mixture was cooled down to 0 °C and NaH was added (0.73 g, 30.41 mmol) portionwise. The reaction mixture was allowed to warm up gradually. After stirring for 1 h at rt, the reaction was quenched by stirring with ice water (50 mL). The organic phase was extracted with ethyl acetate/diethyl ether 1:1 (v/v, 3 × 40 mL) and the combined organic extract was washed with water (3 × 20 mL). The organic phase was separated, dried with $MgSO_4$ and concentrated in vacuo. The residue was purified by column chromatography on silica gel (ethyl acetate/hexanes gradient elution) to afford the title compound (2.6 g, 98%) as a white solid. Analytical data for **1d**: R_f 0.80 (ethyl acetate/hexanes 2:3, v/v); $[\alpha]_D^{24}$ −22.3 (*c* 1.0, $CHCl_3$); ^1H NMR: δ 3.39–3.50 (m, 2H, $PhCH_2CH=CH_2$), 3.60–3.71 (m, 4H, H-3, H-4, H-6a, H-6b), 3.97 (m, 1H, H-5), 4.15 (dd, 1H, $J_{2,3}$ =8.3 Hz, H-2), 4.43 (dd, 2H, *J* = 11.6 Hz, CH_2Ph), 4.66 (d, 1H, ^2J = 8.4 Hz, 1/2 × CH_2Ph), 4.72–4.80 (s, 2H, CH_2Ph), 4.88–5.05 (m, 6H, $J_{1,2}$ = 7.8 Hz, H-1, $PhCH_2CH=CH_2$, 1.5×CH_2Ph), 5.96–6.01 (m, 1H, $PhCH_2CH=CH_2$), 6.96–7.34 (m, 24H, aromatic) ppm; ^{13}C NMR: δ 34.2, 69.1, 73.1, 73.2,

73.3, 73.8, 74.1, 74.5, 74.7, 75.7, 79.2, 82.7, 101.8, 115.1, 115.4, 116.0, 116.2, 127.7, 127.8, 127.9, 128.1, 128.2 (×2), 128.3, 128.4, 128.5 (×2), 128.6 (×3), 128.7 (×2), 129.7, 130.0, 136.9, 138.1, 138.5, 138.6, 138.7, 155.2 ppm; HRMS–MS (*m*/*z*): $[M + Na]^+$ calcd for $C_{43}H_{44}O_6Na^+$ 679.3036, found 679.3019.

Ethyl 2,3,4,6-tetra-*O*-benzyl-1-thio-β-D-glucopyranoside (24). Analytical data for the title compound was essentially the same as previously described [2].

Thiazolinyl 2,3,4,6-tetra-*O*-benzyl-1-thio-β-D-glucopyranoside (27). Analytical data for the title compound was essentially the same as previously described [3].

Tolyl 2,3,4,6-tetra-*O*-benzyl-1-thio-β-D-glucopyranoside (28). Analytical data for the title compound was essentially the same as previously described [4].

Phenyl 2,3,4,6-tetra-*O*-benzyl-1-thio-β-D-glucopyranoside (29). Analytical data for the title compound was essentially the same as previously described [5].

Synthesis of glycosyl acceptors

Methyl 2,3,4-tri-*O*-benzyl-α-D-glucopyranoside (2). Analytical data for the title compound was essentially the same as previously described [1,6].

Methyl 2,3,6-tri-*O*-benzyl-α-D-glucopyranoside (3). Analytical data for the title compound was essentially the same as previously described [1,7].

Methyl 2,4,6-tri-*O*-benzyl-α-D-glucopyranoside (4). Analytical data for the title compound was essentially the same as previously described [7,8].

Methyl 3,4,6-tri-*O*-benzyl-α-D-glucopyranoside (5). Analytical data for the title compound was essentially the same as previously described [7,8].

Allylphenyl 2,3,4-tri-*O*-benzoyl-β-D-glucopyranoside (13). To a stirred solution of 2-allylphenyl 2,3,4-tri-*O*-benzoyl-6-*O*-triphenylmethyl-β-D-glucopyranoside (see below for the synthesis, 1.1 g, 1.22 mmol) in CH_2Cl_2 (20 mL), water (0.2 mL) followed by trifluoroacetic acid (1.8 mL) were added until a persistent yellow color was obtained. The resultant mixture was stirred at rt for 45 min, diluted with CH_2Cl_2 (20 mL), and washed with water (10 mL), sat. aq. $NaHCO_3$ (10 mL), and water (3 × 10 mL). The organic layer was separated, dried with $MgSO_4$, and concentrated in vacuo. The residue was purified by column chromatography on silica gel (ethyl acetate/hexanes gradient elution) to afford the title compound (0.7 g, 90%) as a white solid. Analytical data for **13**: R_f 0.56 (ethyl acetate/hexanes 2:5, v/v); $[\alpha]_D^{23}$ +15.6 (*c* 1.0, $CHCl_3$); ^1H NMR: δ 3.13 (t, *J* =

6.2 Hz, 1H, OH), 3.35 (d, 2H, J = 6.5 Hz, PhCH_2CH=CH_2), 3.87–4.02 (m, 2H, H-6a, H-6b), 4.08–4.13 (m, 1H, H-5), 4.89 (m, 2H, PhCH_2CH=CH_2), 5.57 (d, 1H, $J_{1,2}$ = 7.9 Hz, H-1), 5.73 (dd, 1H, $J_{3,4}$= 9.7 Hz, H-4), 5.77–5.90 (m, 1H, PhCH_2CH=CH_2), 5.99 (dd, 1H, $J_{2,3}$ = 9.8 Hz, H-2), 6.20 (dd, 1H, $J_{3,4}$ = 9.7 Hz, H-3), 7.08–7.56 (m, 13H, aromatic), 7.95–8.09 (m, 6H, aromatic) ppm; ^{13}C NMR: δ 33.9, 61.6, 69.6, 71.7, 72.9, 75.1, 77.6, 99.6, 114.9, 115.7, 123.3, 127.6, 128.5 (×3), 128.7 (×3), 128.9 (×2), 129.3, 129.9 (×2), 129.9 (×2), 130.1 (×2), 130.4, 133.5 (x2), 133.9, 136.5, 154.7, 165.2, 165.9, 166.1 ppm; HRMS–MS (*m*/*z*): [M + Na]$^+$ calcd for $C_{36}H_{32}O_9Na$ 631.1944, found 631.1937

Ethyl 2,3,4-tri-*O*-benzyl-1-thio-β-D-glucopyranoside (15). Analytical data for the title compound was essentially the same as previously described [9].

Tolyl 2,3,4-tri-*O*-benzoyl-1-thio-β-D-glucopyranoside (17). Analytical data for the title compound was essentially the same as previously described [10].

Phenyl 2,3,4-tri-*O*-benzyl-1-thio-β-D-glucopyranoside (19). Analytical data for the title compound was essentially the same as previously described [11].

Phenyl 2,3,4-tri-*O*-benzoyl-1-thio-β-D-glucopyranoside (21). Analytical data for the title compound was essentially the same as previously described [12].

2-Allylphenyl 2,3,4-tri-*O*-benzyl-β-D-glucopyranoside (25). A solution of 2-allylphenyl 6-*O*-acetyl-2,3,4-tri-*O*-benzyl-β-D-glucopyranoside (see below for the synthesis, 0.42 g, 0.69 mmol) was dissolved in methanol (3.2 mL) and the pH was adjusted to pH 9 by

careful addition of a 1 M solution of $NaOCH_3$ in MeOH (~0.1 mL). The reaction mixture was kept for 1 h at rt, then Dowex (H^+) was added until a neutral pH was reached. The resin was filtered off and washed with methanol (3 × 5 mL). The combined filtrate was concentrated in vacuo and dried. The residue was purified by column chromatography on silica gel (ethyl acetate/hexanes gradient elution) to afford the title compound (0.270 g, 69%) as a white solid. Analytical data for **25**: R_f 0.56 (ethyl acetate/hexanes 2:5, v/v); $[\alpha]_D^{23}$ −15.1 (*c* 1.0, $CHCl_3$); ^{1}H NMR: δ 1.82 (s, 1H, OH), 3.35–3.44 (m, 2H, H-4, $PhCH_2CH{=}CH_2$), 3.55–3.75 (m, 4H, $J_{2,3}$ = 8.7 Hz, H-2, H-3, H-5, H-6a), 3.80 (dd, 1H, $J_{6a,6b}$ = 12.1 Hz, $J_{5,6b}$ = 2.6 Hz, H-6b), 4.59 (d, ^2J = 10.9 Hz, 1H, 1/2 CH_2Ph), 4.74–4.98 (m, 7H, $PhCH_2CH{=}CH_2$, 2.5 × CH_2Ph), 5.04 (d, 1H, $J_{1,2}$ = 7.2 Hz, H-1), 5.83–5.96 (m, 1H, $PhCH_2CH{=}CH_2$), 6.91–7.24 (m, 19H, aromatic) ppm; ^{13}C NMR: δ 34.2, 62.2, 75.3, 75.5, 75.9, 82.3, 84.9, 101.1, 114.9, 116.2, 122.9, 127.7, 127.9 (×3), 128.0 (×2), 128.1 (×3), 128.3 (×3), 128.6 (×3), 128.7 (×3), 129.7, 130.4, 136.9, 138.0, 138.3, 138.6, 154.8 ppm; HRMS–MS (*m*/*z*): $[M + Na]^+$ calcd for $C_{36}H_{38}O_6Na$ 589.2668, found 589.2676.

Synthesis of additional building blocks

2-Allylphenyl 2,3,4,6-tetra-*O*-acetyl-β-D-glucopyranoside (S1).

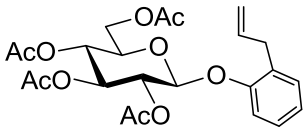

2-Allylphenol (1.37 mL, 10.25 mmol), $BF_3{\cdot}OEt_2$ (1.6 mL, 12.8 mmol), and triethylamine (0.36 mL, 2.56 mmol) were added to a stirred solution of 1,2,3,4,6-penta-*O*-acetyl-β-D-glucopyranoside (2.00 g, 5.13 mmol) in CH_2Cl_2 (40 mL). The reaction mixture was kept for 16 h at rt, then it was diluted with CH_2Cl_2 (30 mL) and washed with water (10 mL), sat. aq. $NaHCO_3$ (10 mL), and water (3 × 10 mL). The organic phase was separated, dried over $MgSO_4$, and concentrated in vacuo. The residue was purified by column chromatography on silica gel (ethyl acetate/hexane gradient elution) to afford the title compound (1.9 g, 90% yield) as white crystals. Analytical data for **S1**: R_f 0.50 (ethyl acetate/hexane 1:5, v/v); mp 145–148 °C (diethyl ether/hexanes); $[\alpha]_D^{23}$ −25.8 (*c* 1.0, $CHCl_3$); ^1H NMR: δ 2.02 (s, 3H, OCH_3), 2.03 (s, 3H, OCH_3), 2.04 (s, 3H, OCH_3), 2.06 (s, 3H, OCH_3), 3.23–3.38 (m, 2H, $PhCH_2CH{=}CH_2$), 3.81–3.87 (m, 1H, H-5), 4.16 (dd, 1H, $J_{6a,6b}$ = 12.2 Hz, $J_{5,6b}$ = 2.5 Hz, H-6b), 4.27 (dd, 1H, $J_{5,6a}$ = 5.5 Hz, H-6a), 4.95–5.05 (m, 3H, H-2, $PhCH_2CH{=}CH_2$), 5.12–5.19 (m, 1H; H-4), 5.24–5.35 (m, 2H, $J_{1,2}$ = 8.7 Hz, H-1, H-3), 5.83–5.96 (m, 1H, $PhCH_2CH{=}CH_2$), 6.97–7.18 (m, 4H, aromatic) ppm; ^{13}C NMR: δ 20.7, 20.8, 20.9, 21.0, 33.9, 62.1, 68.5, 71.2, 72.1, 72.9, 99.3, 115.4, 115.9, 123.5, 127.5, 130.0, 130.5, 136.6, 154.6, 169.3, 169.5, 170.4, 170.7 ppm; HRMS–MS (*m*/*z*): $[M + Na]^+$ calcd for $C_{23}H_{28}O_{10}Na^+$, 487.1580; found, 487.1562.

2-Allylphenyl 2,3,4,6-tetra-*O*-acetyl-β-D-galactopyranoside (S2).

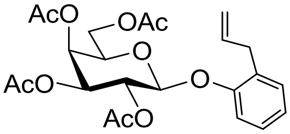

2-Allylphenol (1.37 mL, 10.25 mmol), $BF_3{\cdot}OEt_2$ (1.6 mL, 12.8 mmol), and triethylamine (0.36 mL, 2.56 mmol) were added to a stirred solution of 1,2,3,4,6-penta-*O*-acetyl-β-D-galactopyranoside (2.00 g, 5.13 mmol) in CH_2Cl_2 (40 mL). The reaction mixture was kept for 16 h at rt, then it was diluted with CH_2Cl_2 (30 mL) and washed with water (10 mL), sat. aq. $NaHCO_3$ (10 mL), and water (3 × 10 mL). The organic phase was separated, dried over $MgSO_4$, and concentrated in vacuo. The residue was purified by column chromatography on silica gel (ethyl acetate/hexane gradient elution) to afford the title compound (1.8 g, 89% yield) as white crystals. Analytical data for **S2**: R_f 0.51 (ethyl acetate/hexane 2:3, v/v); mp 98–101 (diethyl ether/hexanes); $[\alpha]_D^{24}$ −13.0 (*c* 1.0, $CHCl_3$); ^{1}H NMR: δ, 2.23–2.42 (m, 12H, 4 × OCH_3), 3.55–3.63 (m, 2H, PhCH_2CH=CH_2), 4.33 (m, 1H, H-5), 4.40 (dd, 1H, $J_{6a,6b}$ = 11.3 Hz, $J_{5,6a}$ = 6.1 Hz, H-6a), 4.48 (dd, 1H, $J_{5,6b}$ = 7.2 Hz, H-6b), 5.23 (d, 1H, $J_{1,2}$ = 8.0 Hz, H-1), 5.28 (t, 2H, PhCH_2CH=CH_2), 5.36 (dd, 1H, $J_{3,4}$ = 3.4 Hz, H-3), 5.70 (dd, 1H, H-4), 5.78 (dd, 1H, $J_{2,3}$ = 10.4 Hz, H-2), 6.12–6.24 (m, 1H, PhCH_2C*H*=CH_2), 7.10–7.29 (m, 4H; aromatic) ppm; ^{13}C NMR: δ 20.6, 20.7, 20.8, 20.9, 33.8, 61.5, 67.0, 68.6, 70.9, 71.0, 99.6, 115.1, 115.7, 123.3, 127.4, 129.8, 130.3, 136.6, 154.6, 169.3, 170.1, 170.3, 170.5 ppm; HRMS–MS (*m*/*z*): $[M + Na]^+$ calcd for $C_{23}H_{28}O_{10}Na^+$, 487.1580; found, 487.1573.

2-Allylphenyl 2,3,4-tri-*O*-benzoyl-6-*O*-triphenylmethyl-β-D-glucopyranoside (S3).

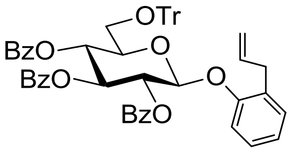

S3

Compound **S1** (1.00 g, 2.16 mmol) was dissolved in methanol (8 mL) and the pH was adjusted to pH 9 by careful addition of a 1 M solution of $NaOCH_3$ in MeOH (~0.1 mL). The reaction mixture was kept for 1 h at rt, then Dowex (H^+) was added until neutral pH was reached. The resin was filtered off and rinsed with methanol (3 × 5 mL). The combined filtrate (~30 mL) was concentrated in vacuo and dried. The resultant solid was dissolved in pyridine (5.3 mL), and triphenylmethyl chloride (1.9 g, 6.76 mmol) was added and the resulting reaction mixture was stirred for 16 h. After that, the reaction mixture was cooled to 0 °C and benzoyl chloride (1.6 mL, 13.5 mmol) was added dropwise. The reaction mixture was allowed to gradually warm to rt and stirred for an additional 3 h at rt. The reaction was quenched by addition of methanol (10 mL), evaporated under reduced pressure and coevaporated with toluene (3 × 20 mL). The residue was diluted with CH_2Cl_2 (20 mL) and washed with water (10 mL), sat. aq. $NaHCO_3$ (10 mL) and water (3 × 10 mL). The organic layer was separated, dried with $MgSO_4$, and concentrated in vacuo. The residue was purified by column chromatography on silica gel (ethyl acetate/hexanes gradient elution) to afford the title compound (1.5 g, 86%) as a white solid. Analytical data for **S3**: R_f 0.50 (ethyl acetate/hexanes 2:5, v/v); ^{1}H NMR: δ 3.33 (d, 2H, J = 6.5 Hz, PhCH_2CH=CH$_2$), 3.43 (dd, 1H, $J_{5,6a}$ = 2.2 Hz, $J_{6b,6a}$ = 10.7 Hz, H-6a), 3.52 (dd, 1H, $J_{5,6b}$ = 6.1Hz, H-6b), 4.03–4.09 (m, 1H, H-5), 4.80–4.92 (m, 2H, PhCH$_2$CH=CH_2), 5.40–5.43 (d, 1H, $J_{1,2}$ = 7.6 Hz, H-1), 5.65–5.72 (m, 1H, H-4), 5.76–5.89 (m, 1H, PhCH$_2$CH=CH$_2$), 5.93–6.01 (m, 2H, H-2, H-

3), 7.09–8.04 (m, 34H, aromatic) ppm; ^{13}C NMR: δ 34.1, 60.6, 62.6, 69.6, 71.9, 73.3, 74.5, 74.6, 87.2 (×2), 100.0, 115.6, 115.7, 115.9, 123.4 (×2), 127.2, 127.6 (×5), 127.9, 128.4, 128.5, 128.7 (×6), 129.0, 129.1, 129.2, 129.4, 129.9, 129.9, 130.0 (×2), 130.1, 130.2, 133.4, 133.4, 136.6, 143.7 (×5), 155.1, 165.1 (×2), 165.3 (×2), 166.0 (×2) ppm; HRMS–MS (*m*/*z*): $[M + Na]^+$ calcd for $C_{55}H_{46}O_9Na$ 873.3072, found 873.3064.

2-Allylphenyl 6-*O*-acetyl-2,3,4-tri-*O*-benzyl-β-D-glucopyranoside (S4).

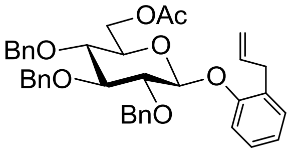

S4

To a stirred solution of a **1a** (0.1 g, 0.15 mmol) in Ac_2O/AcOH 2:1 (v/v, 0.9 mL) was added freshly prepared $ZnCl_2$ (166 mg, 1.22 mmol) solution in Ac_2O/AcOH 2:1 (v/v, 0.86 mL). The reaction mixture was stirred under argon for 4 h at rt. Upon completion, the reaction was quenched with H_2O, diluted with ethyl acetate (50 mL), and washed with water (20 mL), sat. aq. $NaHCO_3$ (20 mL) and water (3 × 20 mL). The organic phase was separated, dried over $MgSO_4$, and concentrated in vacuo. The residue was purified by column chromatography on silica gel (ethyl acetate/toluene gradient elution) to afford the title compound (98 mg, 99%) as a clear form. Analytical data for **S4**: R_f 0.51 (ethyl acetate/toluene 1:10, v/v); $[\alpha]_D^{23}$ −5.8 (*c* 1.0, $CHCl_3$); ^{1}H NMR: δ 1.92 (s, 3H, OCH_3), 3.29–3.45 (m, 2H, $PhCH_2CH{=}CH_2$), 3.48–3.65 (m, 2H, H-4, H-5), 3.67–3.71 (m, 2H, H-2, H-3), 4.14 (dd, 1H, $J_{6a,6b}$ = 11.8 Hz, $J_{5,6a}$ = 5.1 Hz, H-6a), 4.24 (dd, 1H, $J_{5,6b}$ = 1.7 Hz, H-6b), 4.51 (d, ^2J = 10.9 Hz, 1/2 CH_2Ph), 4.74–5.01 (m, 8H, H-1, $PhCH_2CH{=}CH_2$, 2.5 × CH_2Ph), 5.83–5.93 (m, 1H, $PhCH_2CH{=}CH_2$), 6.87–7.27 (m, 19H, aromatic) ppm; ^{13}C NMR: δ 20.9, 34.2, 63.3, 73.1, 75.2, 75.3, 75.9, 82.1, 85.0, 101.3, 115.5, 116.1,

123.0, 127.5, 127.9, 128.0, 128.1, 128.2, 128.3, 128.4 (×3), 128.5, 128.6 (×3), 128.6, 128.7 (×3), 129.9, 130.2, 136.8, 137.7, 138.2, 138.4, 154.9, 170.8 ppm; HRMS–MS (*m*/*z*): $[M + Na]^+$ calcd for $C_{38}H_{40}O_7Na$, 631.2674; found, 631.2665.

Data for di- and trisaccharides

Methyl 6-*O*-(2,3,4,6-tetra-*O*-benzyl-α/β-D-glucopyranosyl)-2,3,4-tri-*O*-benzyl-α-D-glucopyranoside (6a). Analytical data for the title compound was similar to that previously described [13].

Methyl 6-*O*-(2,3,4,6-tetra-*O*-benzoyl-β-D-glucopyranosyl)-2,3,4-tri-*O*-benzyl-α-D-glucopyranoside (6b). Analytical data for the title compound was essentially the same as previously described [13].

Methyl 6-*O*-(2-*O*-benzoyl-3,4,6-tri-*O*-benzyl-β-D-glucopyranosyl)-2,3,4-tri-*O*-benzyl-α-D-glucopyranoside (6c). Analytical data for the title compound was essentially the same as previously described [14].

Methyl 6-*O*-(2,3,4,6-tetra-*O*-benzyl-D-galactopyranosyl)-2,3,4-tri-*O*-benzyl-α-D-glucopyranoside (6d). Analytical data for the title compound was essentially the same as previously described [15].

Methyl 4-*O*-(2,3,4,6-tetra-*O*-benzyl-α/β-D-glucopyranosyl)-2,3,6-tri-*O*-benzyl-α-D-glucopyranoside (7a). Analytical data for the title compound was similar to that previously described [13].

Methyl 4-*O*-(2,3,4,6-tetra-*O*-benzyl-D-galactopyranosyl)-2,3,6-tri-*O*-benzyl-α-D-glucopyranoside (7d). Analytical data for the title compound was similar to that previously described [16].

Methyl 3-*O*-(2,3,4,6-tetra-*O*-benzyl-α/β-D-glucopyranosyl)-2,4,6-tri-*O*-benzyl-α-D-glucopyranoside (8a). Analytical data for the title compound was similar to that previously described [17].

Methyl 3-*O*-(2,3,4,6-tetra-*O*-benzyl-D-galactopyranosyl)-2,4,6-tri-*O*-benzyl-α-D-glucopyranoside (8d). Analytical data for the title compound was similar to that previously described [18].

Methyl 2-*O*-(2,3,4,6-tetra-*O*-benzyl-α/β-D-glucopyranosyl)-3,4,6-tri-*O*-benzyl-α-D-glucopyranoside (9a). Analytical data for the title compound was similar to that previously described [19].

Methyl 2-*O*-(2,3,4,6-tetra-*O*-benzoyl-β-D-glucopyranosyl)-3,4,6-tri-*O*-benzyl-α-D-glucopyranoside (9b). Analytical data for the title compound was essentially the same as previously described [19].

Methyl 2-*O*-(2,3,4,6-tetra-*O*-benzyl-D-galactopyranosyl)-3,4,6-tri-*O*-benzyl-α-D-glucopyranoside (9d). The title compound was obtained as a clear film from **1d** and **5** by Method C in 80% yield (α/β = 3.0/1). Selected analytical data for α-**9d**: ^{1}H NMR: δ 3.91 (dd, 1H, $J_{2,3}$ = 7.6 Hz, H-2), 4.02 (dd, 1H, $J_{2',3'}$ = 9.6 Hz, H'-2), 4.93 (d, 1H, $J_{1,2}$ = 3.4 Hz, H-1), 4.97 (d, 1H, $J_{1'2'}$ = 3.5 Hz, H'-1) ppm; ^{13}C NMR: δ 94.9 (C-1), 96.7 (C'-1) ppm; HRMS–MS (*m*/*z*): [M + Na]$^+$ calcd for $C_{62}H_{66}O_{11}Na^+$, 1009.4503; found, 1009.4510.

2-[3-Iodo-2-(methyl 2,3,4-tri-*O*-benzyl-α-D-glucopyranosid-6-yl)propyl]oxyphenyl 2,3,4,6-tetra-*O*-benzyl-β-D-glucopyranoside (12). The title compound was isolated as a by-product from the synthesis of **6a** from **1a** and **2** by Method B in 15% yield. Selected analytical data for **12**: ^{1}H NMR: δ 3.74 (dd, 1H, $J_{2',3'}$ = 7.9 Hz, H'-2), 4.53 (d, $J_{1,2}$ = 4.1 Hz, H-1), 5.02 (d, 1H, $J_{1',2'}$ = 8.0 Hz, H'-1) ppm; ^{13}C NMR: δ 10.6, 35.9, 55.3, 68.0, 68.8, 70.4, 73.5, 73.6, 73.7, 74.8, 75.2, 75.3, 75.4, 75.5, 75.8, 75.9, 77.4, 77.8, 77.9, 79.4, 79.9, 80.1, 82.2, 82.3, 82.4, 85.1, 85.2, 98.2, 101.8, 115.8, 122.8, 122.9, 127.1, 127.6, 127.7, 127.8, 127.9 (×2), 128.0 (×2), 128.1 (×2), 128.1 (×2), 128.2 (×2), 128.3, 128.4 (×2), 128.5 (×2), 128.6 (×4), 128.7 (×4), 128.9, 129.2, 132.2, 138.1, 138.2, 138.3, 138.4, 138.5, 138.6, 138.7, 138.8, 155.5 ppm; HRMS–MS (*m*/*z*): [M + Na]$^+$ calcd for $C_{71}H_{75}IO_{12}Na^+$, 1269.4201; found, 1269.4214.

2-Allylphenyl 6-*O*-(2,3,4,6-tetra-*O*-benzyl-α/β-D-glucopyranosyl)-2,3,4-tri-*O*-benzoyl-β-D-glucopyranoside (14). The title compound was obtained as a clear film from **1a** and **13** by Method B in 78% yield (α/β = 1.0/1). Selected analytical data for α-**14**: ^{1}H NMR: δ 4.58 (d, 1H, $J_{1',2'}$ = 4.6 Hz, H'-1), 5.28 (d, $J_{1,2}$ = 7.8 Hz, H-1) ppm; ^{13}C NMR: δ 80.4 (C-1), 98.4 (C'-1) ppm; Selected analytical data for β-**14**: ^{1}H NMR: δ 4.42 (d, 1H, $J_{1',2'}$ = 11.0 Hz, H'-1) ppm; ^{13}C NMR: δ 82.2 (C-1), 100.1 (C'-1) ppm; HRMS–MS (*m*/*z*): $[M + Na]^+$ calcd for $C_{70}H_{68}O_{14}Na^+$, 1155.4609; found, 1155.4623.

Ethyl 6-*O*-(2,3,4,6-tetra-*O*-benzyl-α/β-D-glucopyranosyl)-2,3,4-tri-*O*-benzyl-1-thio-β-D-glucopyranoside (16). Analytical data for the title compound was essentially the same as previously described [2].

Tolyl 6-*O*-(2,3,4,6-tetra-*O*-benzyl-α/β-D-glucopyranosyl)-2,3,4-tri-*O*-benzoyl-1-thio-β-D-glucopyranoside (18). The title compound was obtained as a clear film from **6** and **15** by Method A in 75% yield (α/β = 2.4/1). Selected analytical data for α-**18**: ^{1}H NMR: δ 4.67 (d, 1H, $J_{1',2'}$ = 3.7 Hz, H'-1), 4.85 (d, 1H, $J_{1,2}$ = 9.3 Hz, H-1) ppm; ^{13}C NMR: δ 86.1 (C-1), 97.6 (C'-1) ppm. Selected analytical data for β-**18**: ^{1}H NMR: δ 4.52 (d, 1H, $J_{1',2'}$ = 10.8 Hz, H'-1) ppm; ^{13}C NMR: δ 87.2 (C-1), 103.9 (C'-1) ppm; HRMS–MS (*m*/*z*): $[M + Na]^+$ calcd for $C_{68}H_{64}O_{13}SNa^+$, 1143.3965; found, 1143.3970.

Phenyl 6-*O*-(2,3,4,6-tetra-*O*-benzyl-α/β-D-glucopyranosyl)-2,3,4-tri-*O*-benzyl-1-thio-β-D-glucopyranoside (20). The title compound was obtained as a clear film from **1a** and **19** by Method B in 90% yield (α/β = 1.0/1). Selected analytical data for α-**20**: ^{1}H NMR: δ 3.95 (t, 1H, $J_{2,3}$ = 9.0 Hz, H-3), 4.57 (d, 1H, $J_{1,2}$ = 9.3 Hz, H-1), 5.00 (d, 1H,

$J_{1',2'}$ = 3.5 Hz, H'-1) ppm; ^{13}C NMR: δ 89.2 (C-1), 96.6 (C'-1) ppm. Selected analytical data for β-**20**: ^{1}H NMR: δ 4.37 (d, 1H, $J_{1',2'}$ = 7.8 Hz, H'-1); ^{13}C NMR: δ 95.2 (C-1), 103.4 (C'-1) ppm. HRMS–MS (*m*/*z*): [M + Na]$^+$ calcd for $C_{67}H_{68}O_{10}SNa^+$, 1087.4433; found, 1087.4406.

Phenyl 6-*O*-(2,3,4,6-tetra-*O*-benzyl-α/β-D-glucopyranosyl)-2,3,4-tri-*O*-benzoyl-1-thio-β-D-glucopyranoside (22). Analytical data for the title compound was essentially the same as previously described [19].

Phenyl 6-*O*-(2-benzoyl-3,4,6-tri-*O*-benzyl-β-D-glucopyranosyl)-2,3,4-tri-*O*-benzoyl-1-thio-β-D-glucopyranoside (23). The title compound was obtained as a clear film from donors **1c** and acceptor **21** by Method B in 75% yield. Selected analytical data for **23**: ^{1}H NMR: δ 3.65 (dd, 1H, $J_{6a,6b}$ = 9.3 Hz, H-6a), 3.81 (dd, 1H, $J_{5,6b}$ = 3.2 Hz, H-6b), 3.87–3.99 (m, 4H, H-5, H'-5, H'-6a, H'-6b), 4.08–4.14 (m, 2H, H'3, H'-4), 4.59 (d, 1H, ^{2}J = 12.2 Hz, 1/2 CH_2Ph), 4.70 (d, 1H, ^{2}J = 11.6 Hz, 1/2 CH_2Ph), 4.78–4.88 (m, 4H, 2 × CH_2Ph), 4.93 (d, 1H, $J_{1',2'}$ = 6.2 Hz, H'-1), 4.96 (d, 1H, $H_{1,2}$ = 5.2 Hz, H-1), 5.39–5.49 (m, 3H, H-2, H-4, H'-2), 5.88 (dd, 1H, $J_{2,3}$ = 9.4 Hz, H-3), 7.27–8.12 (m, 40H, aromatic) ppm; ^{13}C NMR: δ 68.8, 69.5, 70.5, 71.2, 73.7, 74.6, 74.9, 75.3, 77.6, 78.1, 78.5, 83.2, 101.3, 127.7, 127.9, 128.1 (×3), 128.3 (×3), 128.4 (×3), 128.5 (×3), 128.6 (×4), 128.9, 129.0, 129.1 (×3), 129.2 (×3), 129.3 (×2), 129.4 (×3), 129.5 (×2), 129.9 (×2), 130.0 (×3), 130.1 (×4), 130.2, 131.8, 133.2, 133.4, 133.5, 138.0, 138.1, 138.2, 165.2, 165.5, 165.6, 165.9 ppm; HRMS–MS (*m*/*z*): [M + Na]$^+$ calcd for $C_{67}H_{60}O_{14}SNa$, 1143.3604; found, 1143.3636

2-Allylphenyl 6-*O*-(2,3,4,6-tetra-*O*-benzyl-α/β-D-glucopyranosyl)-2,3,4-tri-*O*-benzyl-β-D-glucopyranoside (26). The title compound was obtained as a clear film from donors **24**, **27**–**29** and acceptor **25** by Method A in 82–90% yield. Selected analytical data for α-**26**: ^{1}H NMR: δ 4.37 (d, 1H, $J_{1',2'}$ = 2.7 Hz, H'-1) ppm; ^{13}C NMR: δ 97.6 (C-1), 100.6 (C'-1) ppm. Selected analytical data for β-**26**: ^{1}H NMR: δ 4.34 (d, 1H, $J_{1',2'}$ = 5.6 Hz, H'-1) ppm; ^{13}C NMR: δ 101.6 (C-1), 104.2 (C'-1) ppm; HRMS–MS (*m*/*z*): [M + Na]$^+$ calcd for $C_{70}H_{72}O_{11}Na^+$, 1111.5057; found, 1111.4980.

Methyl O-(2,3,4,6-tetra-*O*-benzyl-α/β-D-glucopyranosyl)-(1→6)-*O*-(2,3,4-tri-*O*-benzyl-α/β-D-glucopyranosyl)-(1→6)-2,3,4-tri-*O*-benzyl-β-D-glucopyranoside (S5). The title compound was obtained from **2** and **26** by Method C in 80% yield and from **2** and **16** by Method C in 89% yield. Analytical data for the title compound was in accordance with that previously described [20].

Phenyl O-(2,3,4,6-tetra-*O*-benzyl-α/β-D-glucopyranosyl)-(1→6)-*O*-(2,3,4-tri-*O*-benzyl-α/β-D-glucopyranosyl)-(1→6)-2,3,4-tri-*O*-benzoyl-1-thio-β-D-glucopyranoside (30). The title compound was obtained as a clear film from **26** and **21** by method B in 90% yield. Selected analytical data for α-**30**: ^{1}H NMR: δ 4.69 (d, 1H, $J_{1',2'}$ = 3.1 Hz, H'-1), 4.96 (d, 1H, $J_{1'',2''}$ = 2.3 Hz, H''-1) ppm; ^{13}C NMR: δ 97.4 (C-1), 97.5 (C'-1), 97.6 (C"-1) ppm. Selected analytical data for β-**31**: ^{1}H NMR: δ 5.27 (d, 1H, $J_{1,2}$ = 7.9 Hz, H-1) ppm; ^{13}C NMR: δ 99.7 (C-1), 99.8 (C'-1), 103.8 (C''-1) ppm; HRMS–MS (*m*/*z*): [M + Na]$^+$ calcd for $C_{94}H_{90}O_{18}SNa^+$, 1561.5745; found, 1561.5731.

2-Allylphenyl O-(2,3,4,6-tetra-*O*-benzyl-α/β-D-glucopyranosyl)-(1→6)-*O*-(2,3,4-tri-*O*-benzyl-α/β-D-glucopyranosyl)-(1→6)-2,3,4-tri-*O*-benzyl-β-D-glucopyranoside (31). The title compound was obtained as a colorless syrup from **16** and **25** by method A in 50% yield. Selected analytical data for β-**31**: ^{1}H NMR: δ 4.27 (d, 1H, $J_{1',2'}$ = 9.0 Hz, H'-1), 4.37 (d, 1H, $J_{1'',2''}$ = 7.1 Hz, H''-1), 5.25 (d, 1H, $J_{1,2}$ = 6.9 Hz, H-1) ppm; ^{13}C NMR: δ 98.2 (C-1), 102.0 (C'-1), 103.8 (C''-1) ppm; HRMS–MS (*m*/*z*): [M + Na]$^+$ calcd for $C_{97}H_{100}O_{16}Na^+$, 1543.6909; found, 1543.6936.

2-Allylphenyl O-(2,3,4,6-tetra-*O*-benzyl-α/β-D-glucopyranosyl)-(1→6)-*O*-(2,3,4-tri-*O*-benzyl-α/β-D-glucopyranosyl)-(1→6)-2,3,4-tri-*O*-benzoyl-β-D-glucopyranoside (32). The title compound was obtained as a colorless syrup from **16** and **13** by method A in 80% yield. Selected analytical data for α-**32**: ^{1}H NMR: δ 4.67 (d, 1H, $J_{1',2'}$ = 3.2 Hz, H'-1), 4.90 (d, 1H, $J_{1'',2''}$ = 2.5 Hz, H''-1) ppm; ^{13}C NMR: δ 97.4 (C-1), 97.5 (C'-1), 97.6 (C"-1) ppm; Selected analytical data for β-**33**: ^{1}H NMR: δ 5.27 (d, 1H, $J_{1,2}$ = 7.8 Hz, H-1) ppm; ^{13}C NMR: δ 99.7 (C-1), 99.8 (C'-1), 103.8 (C''-1) ppm; HRMS–MS (*m*/*z*): [M + Na]$^+$ calcd for $C_{97}H_{94}O_{19}Na^+$, 1585.7729; found, 1585.7762.

NMR spectra

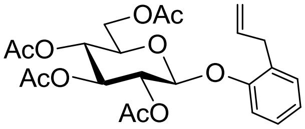

S1

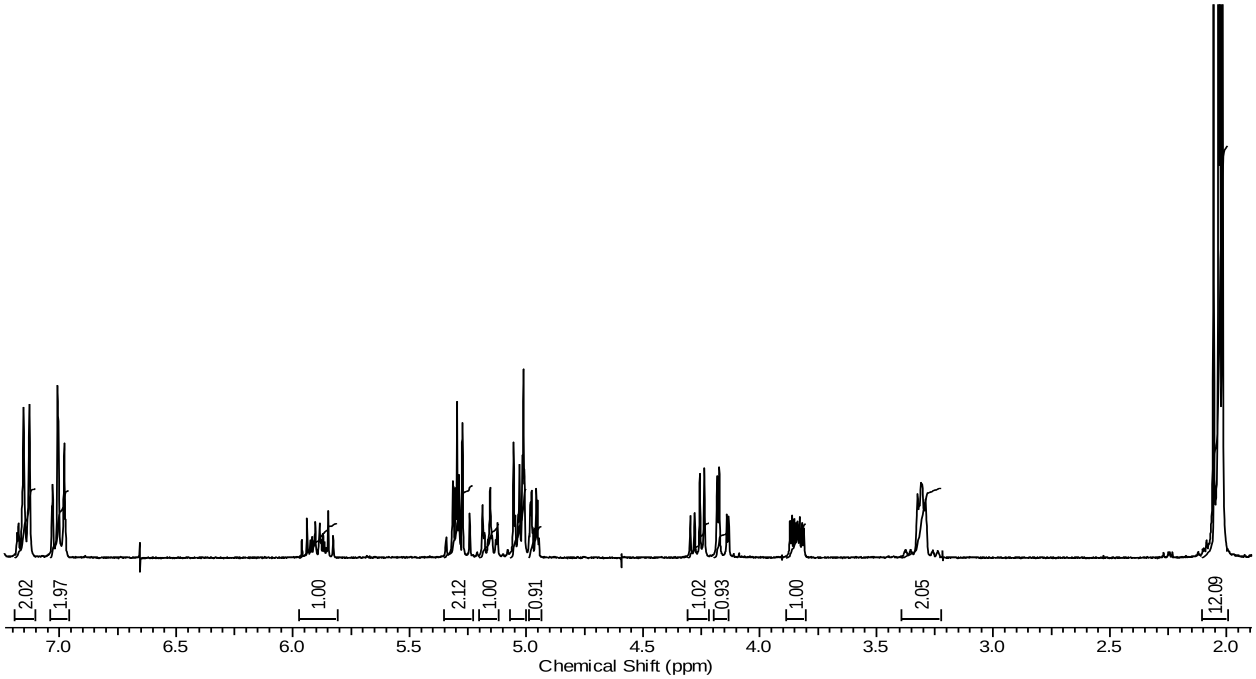

$CDCl_3$ at 300 MHz

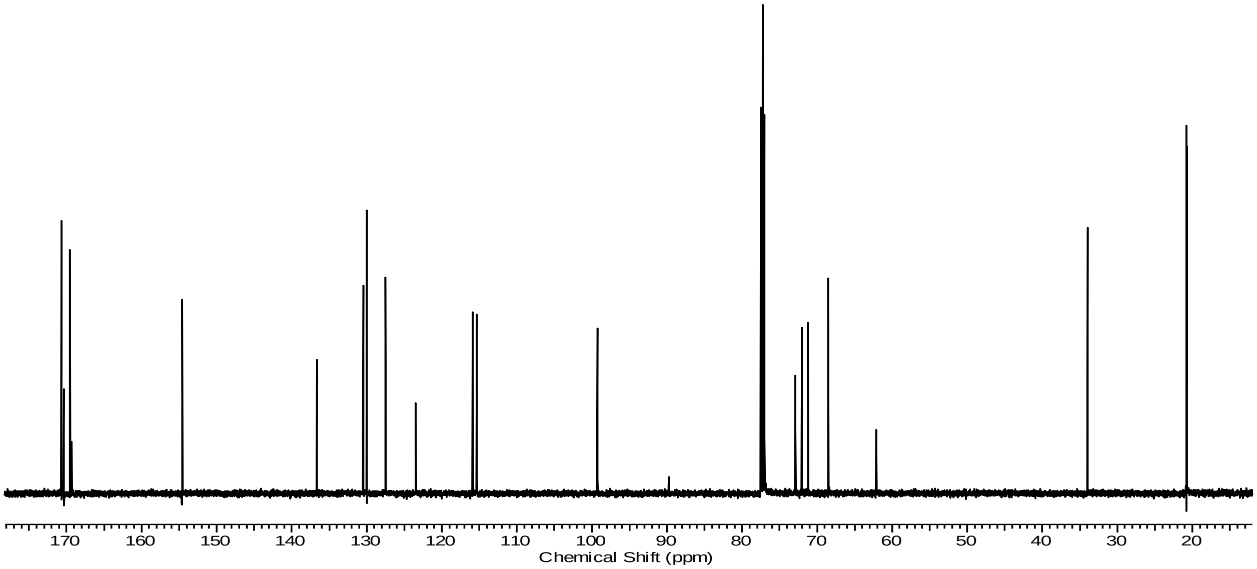

$CDCl_3$ at 75 MHz

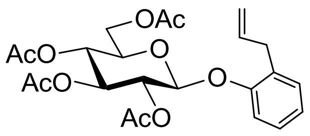

S1

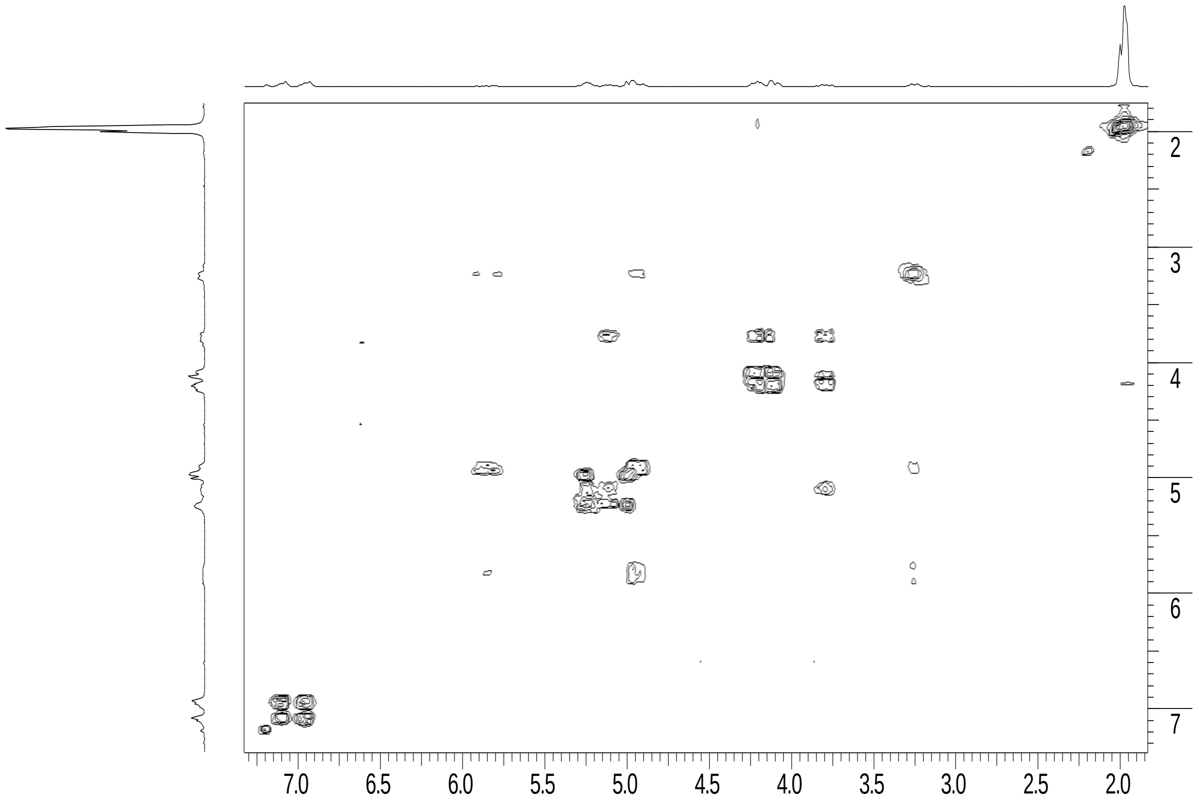

$CDCl_3$ at 300 MHz

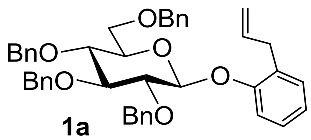

1a

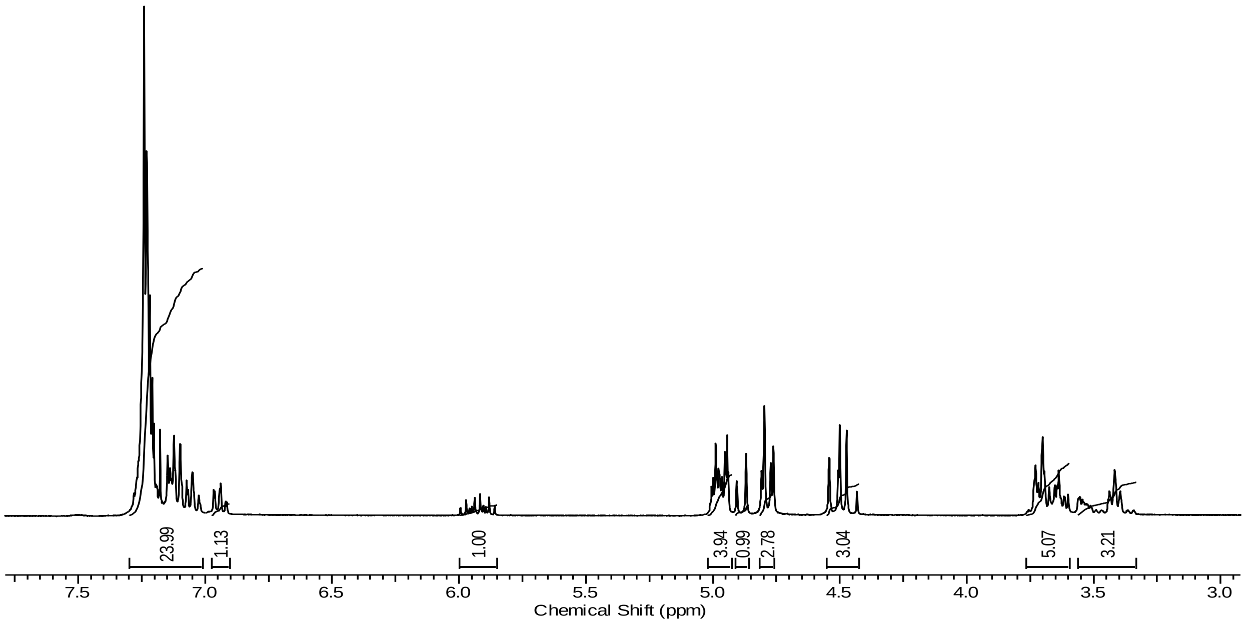

$CDCl_3$ at 300 MHz

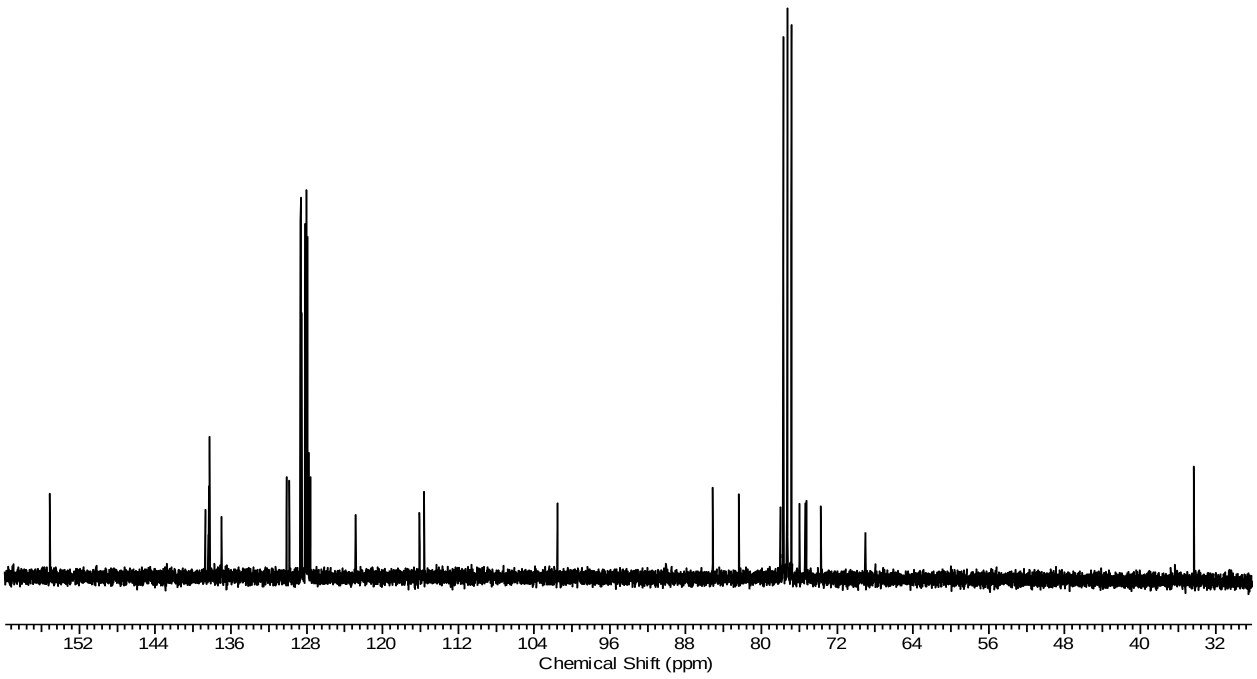

$CDCl_3$ at 75 MHz

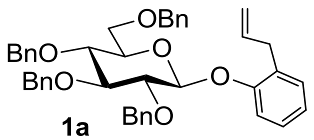

1a

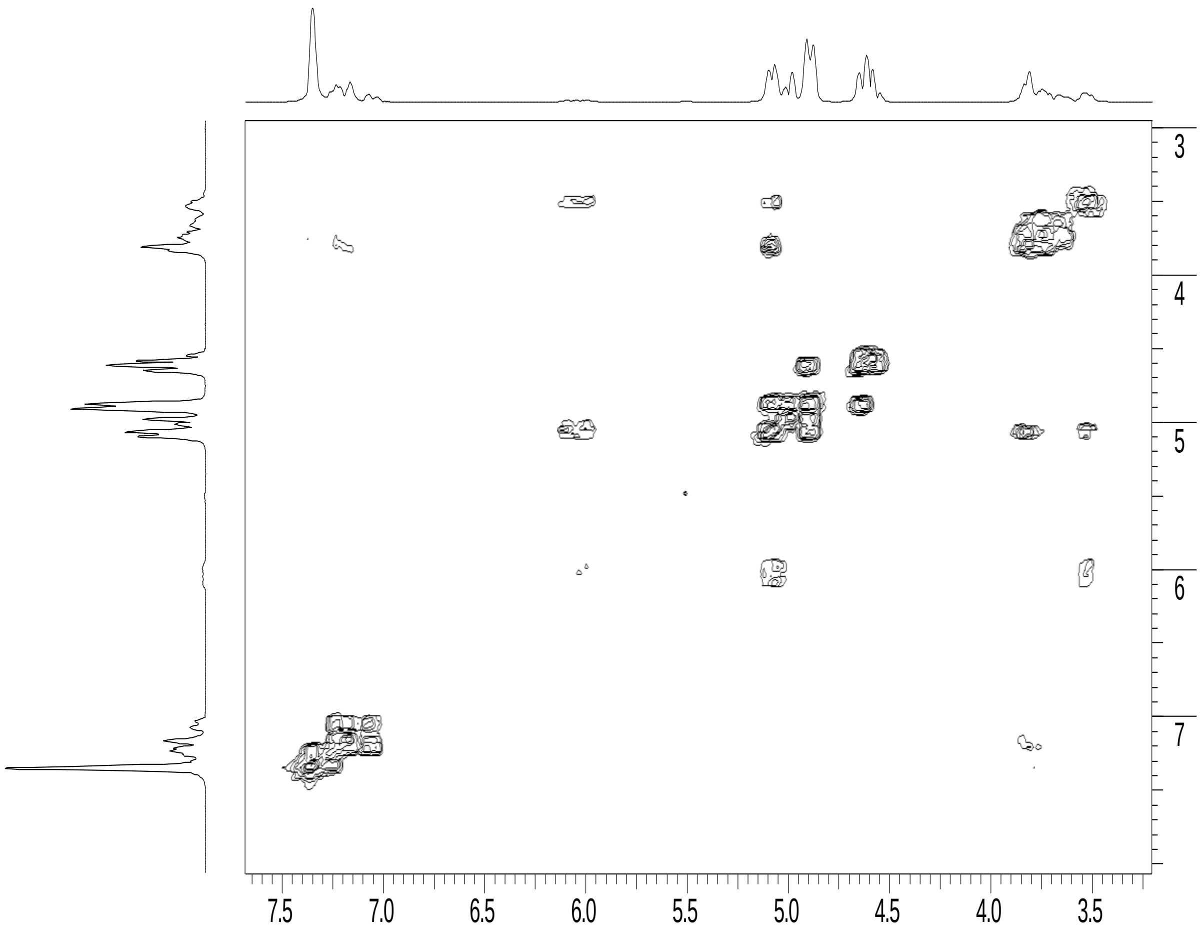

$CDCl_3$ at 300 MHz

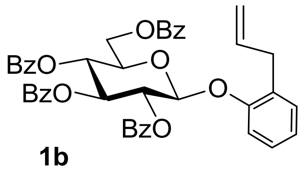

1b

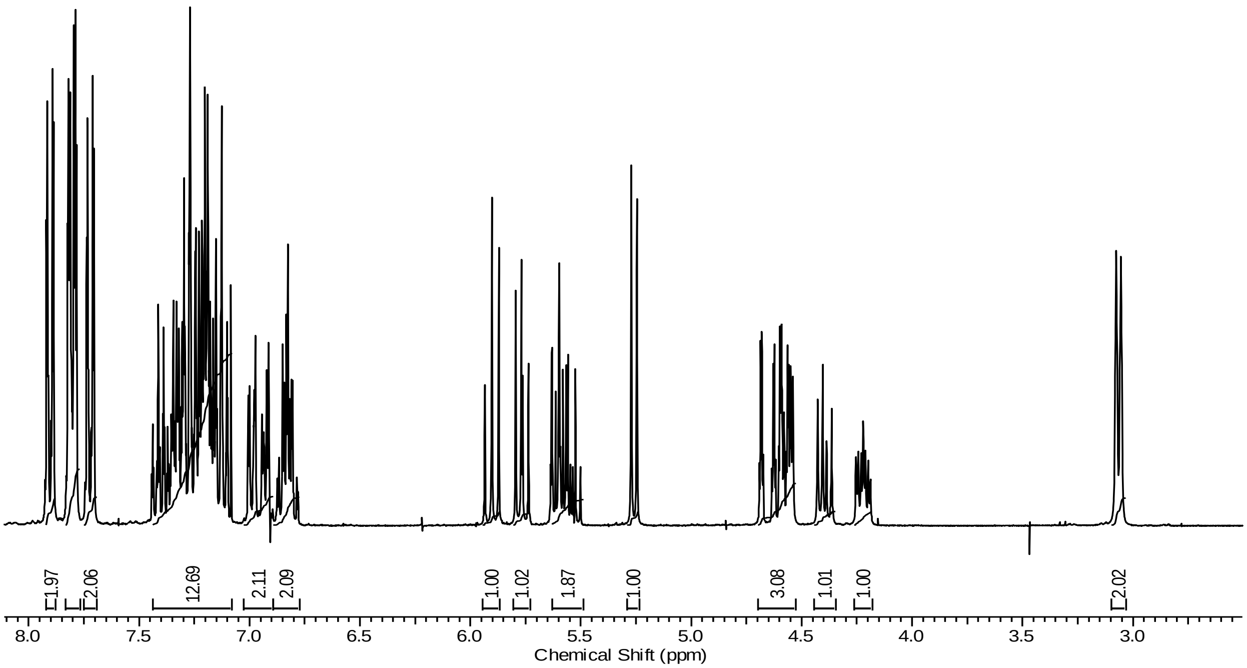

CDCl$_3$ at 300 MHz

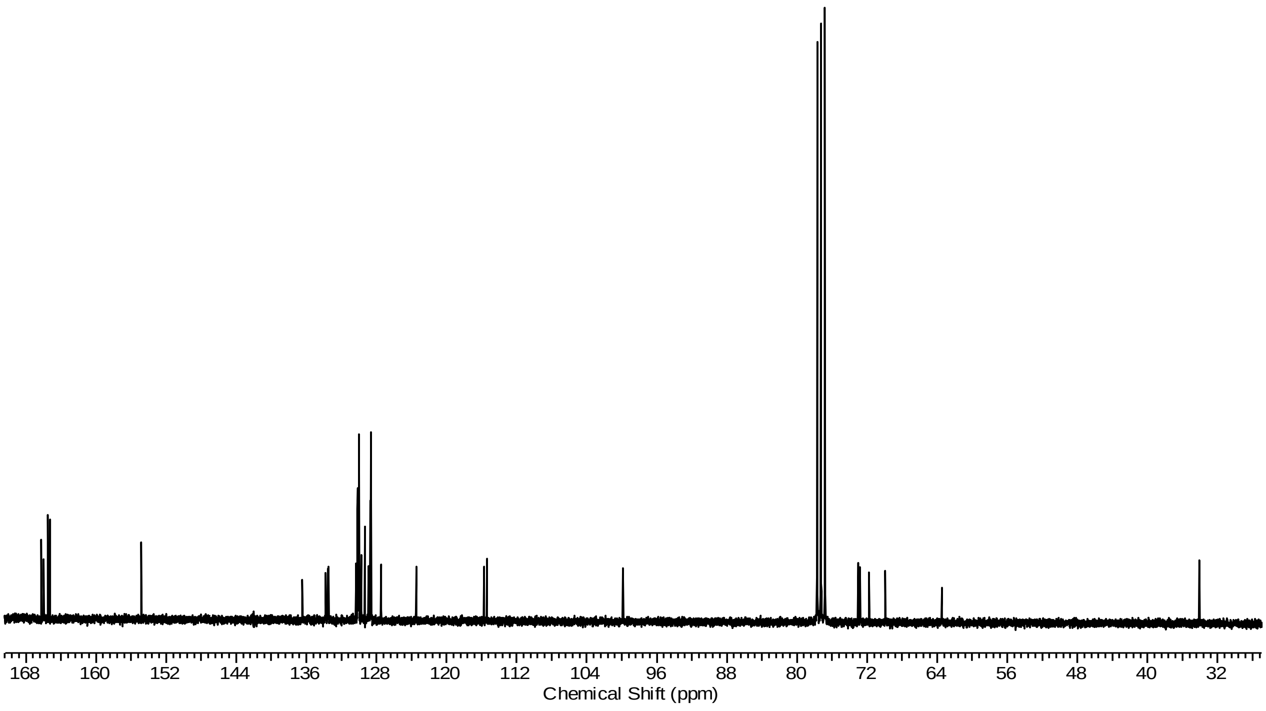

CDCl$_3$ at 75 MHz

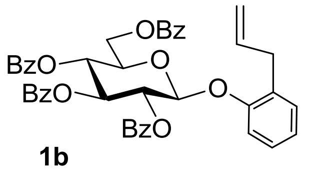

1b

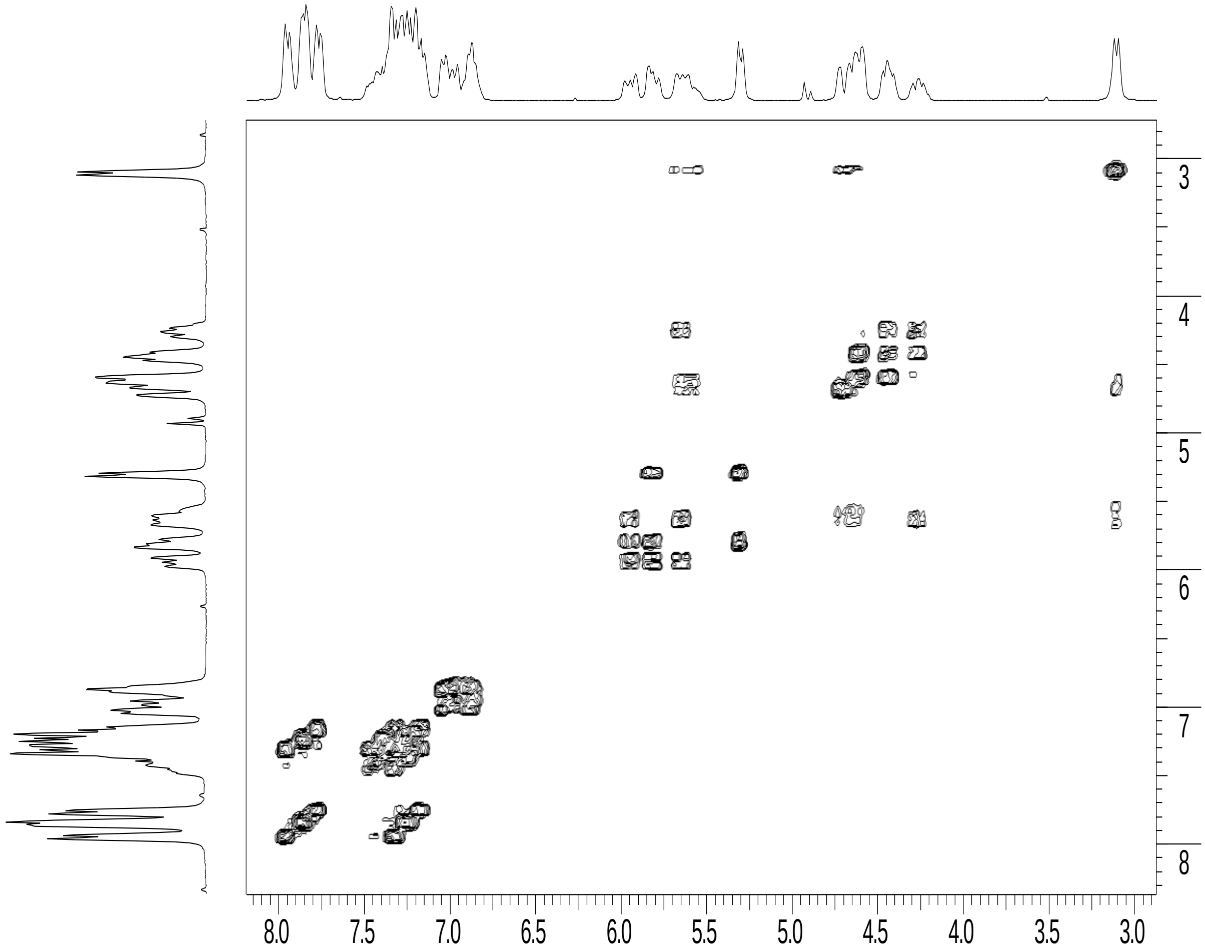

CDCl$_3$ at 300 MHz

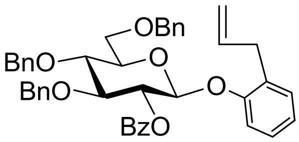

1c

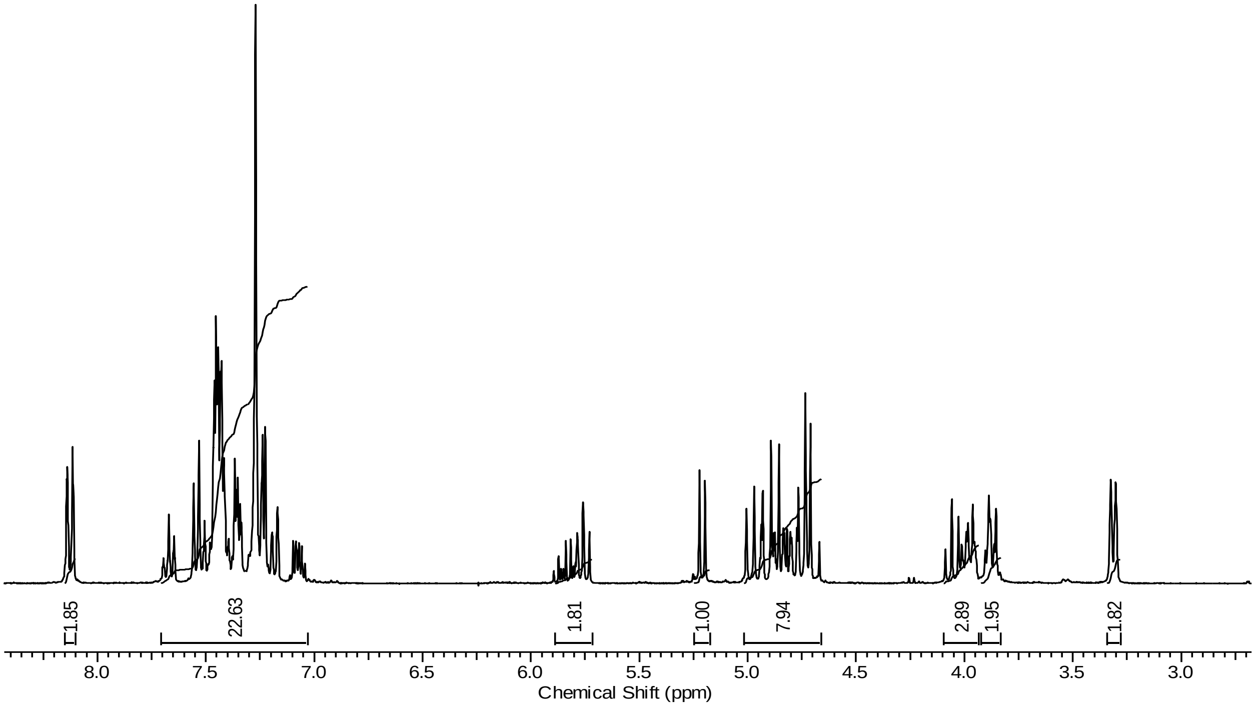

CDCl$_3$ at 300 MHz

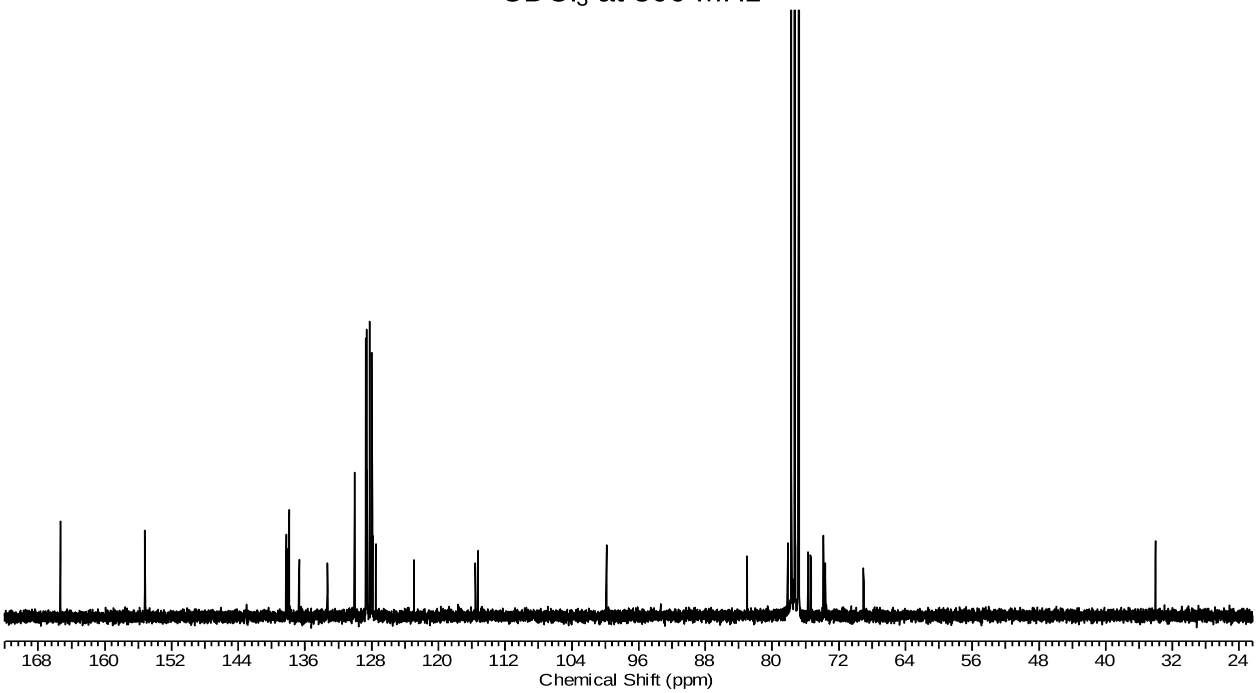

CDCl$_3$ at 75 MHz

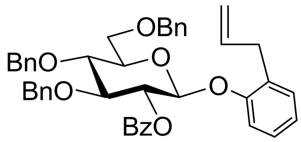

1c

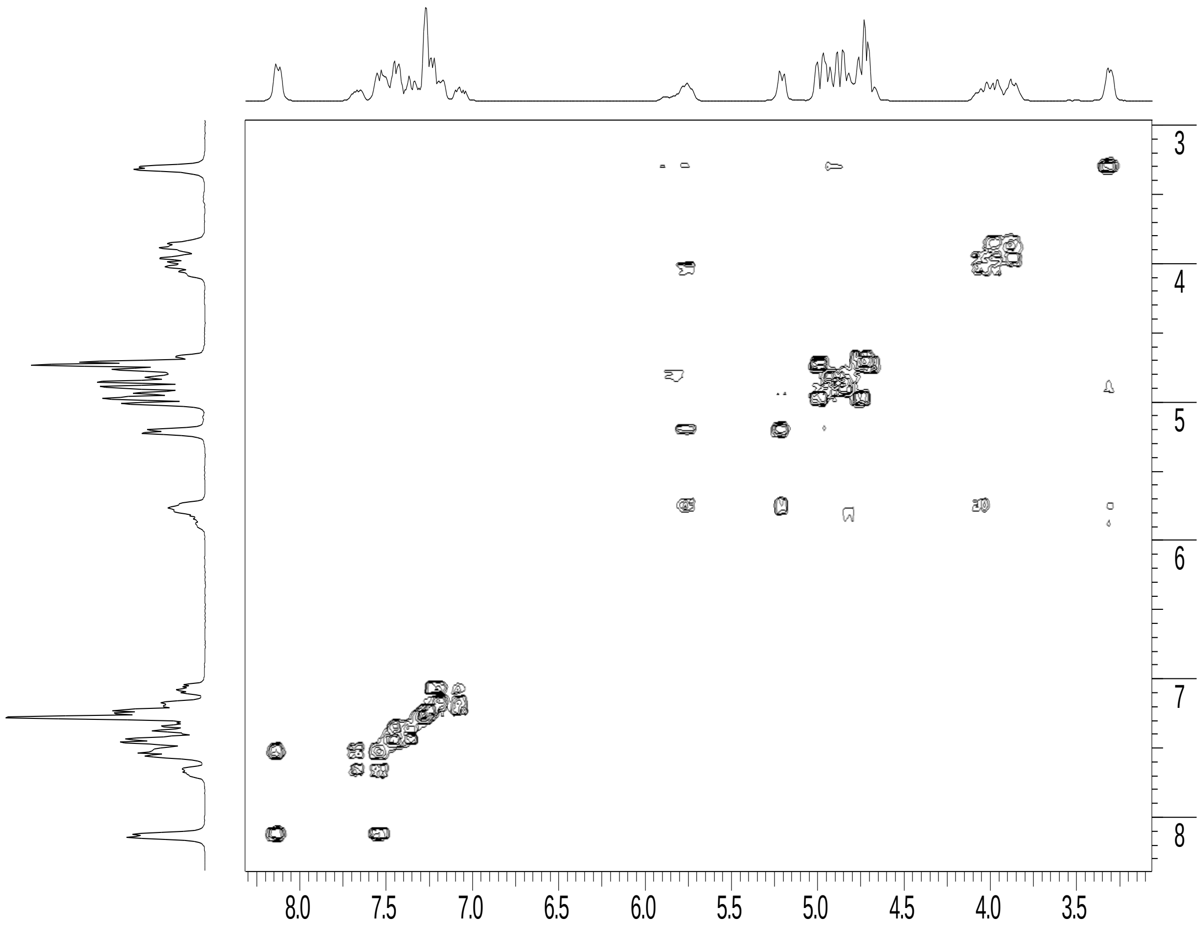

$CDCl_3$ at 300 MHz

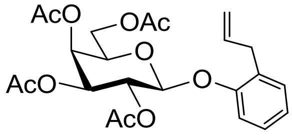

S2

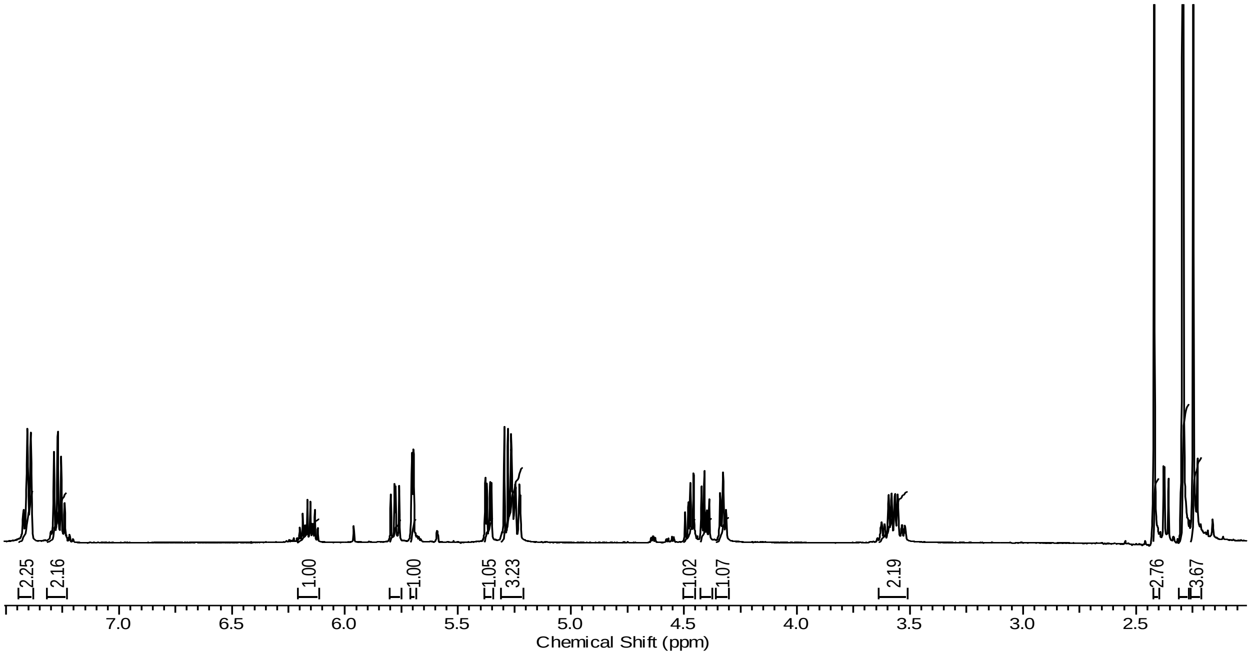

$CDCl_3$ at 300 MHz

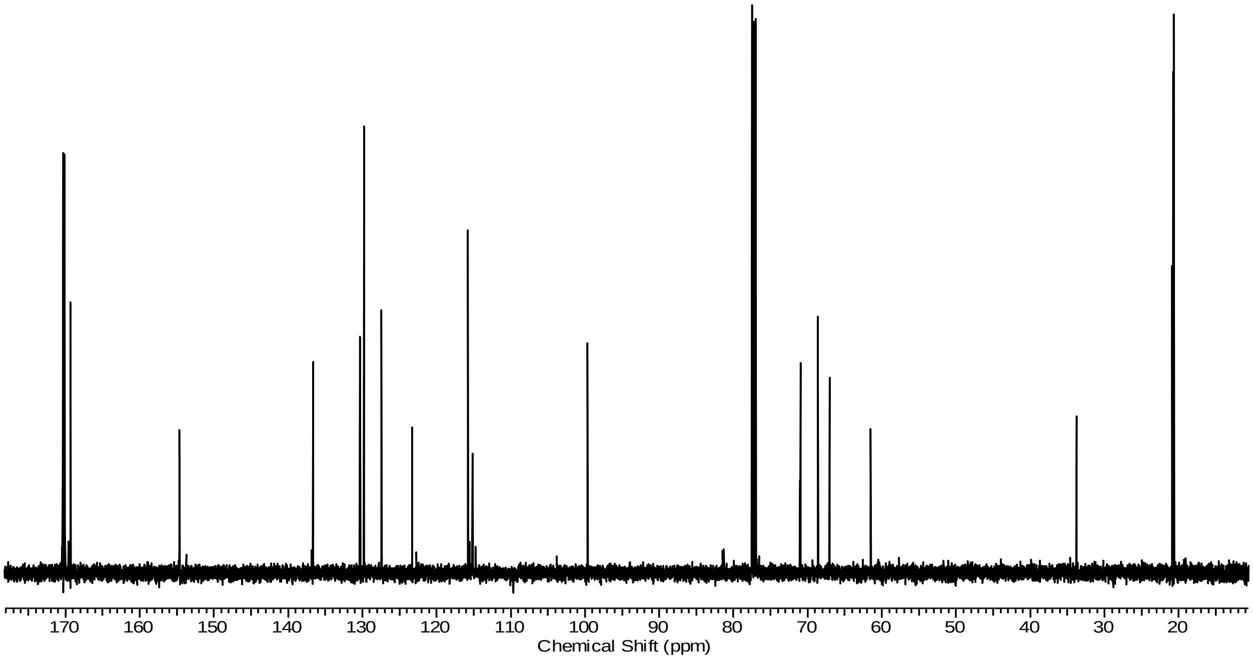

$CDCl_3$ at 75 MHz

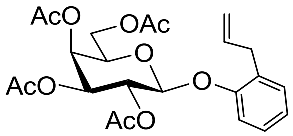

S2

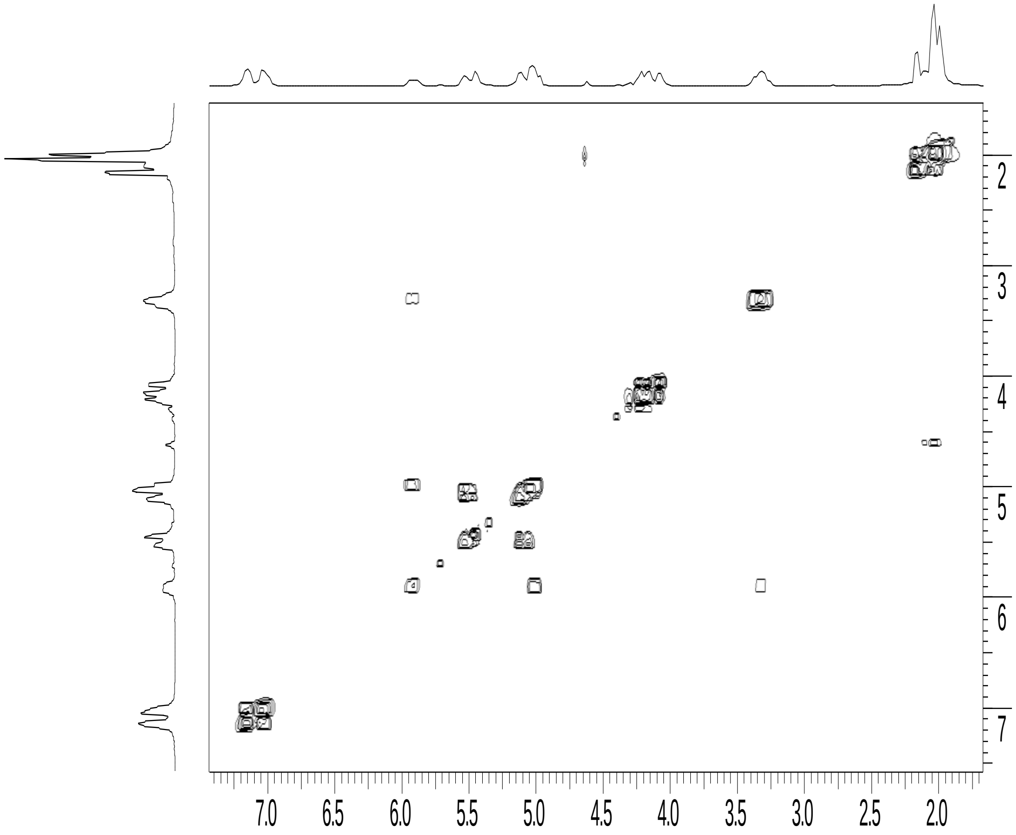

CDCl$_3$ at 300 MHz

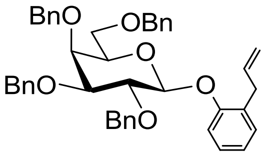

1d

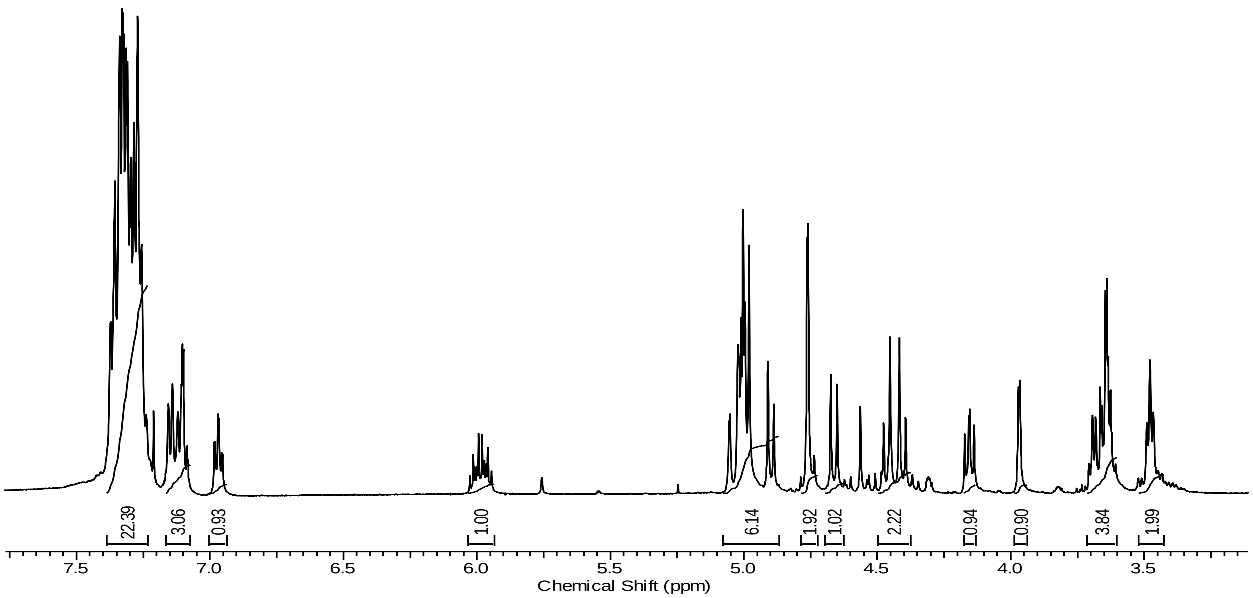

CDCl$_3$ at 300 MHz

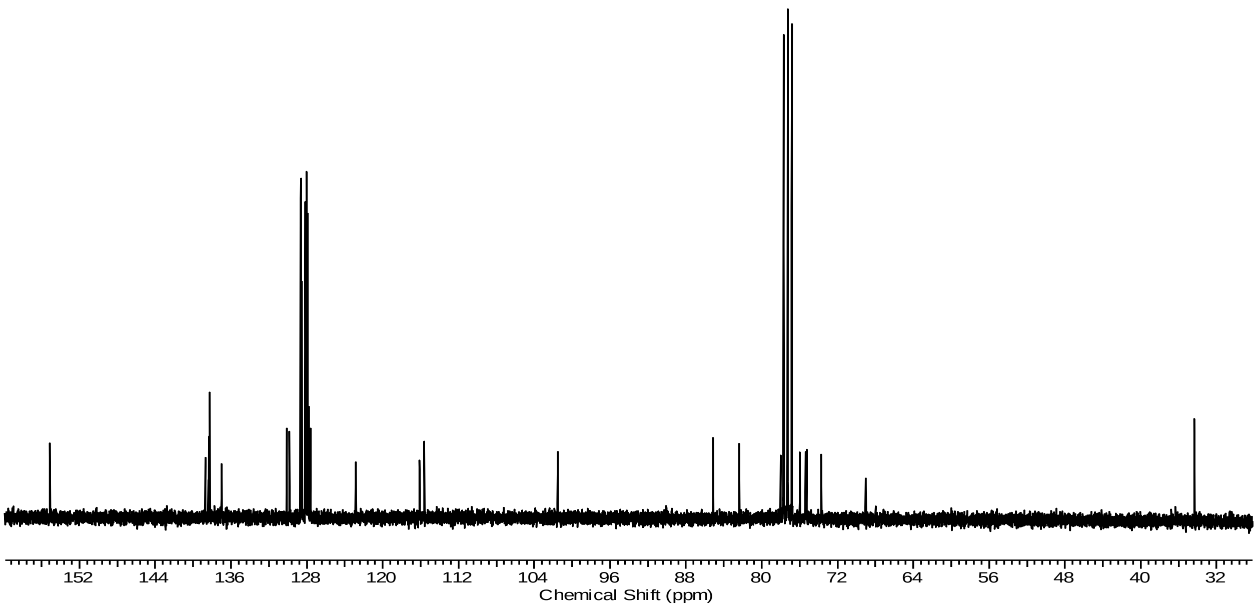

CDCl$_3$ at 75 MHz

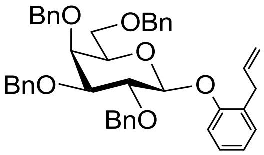

1d

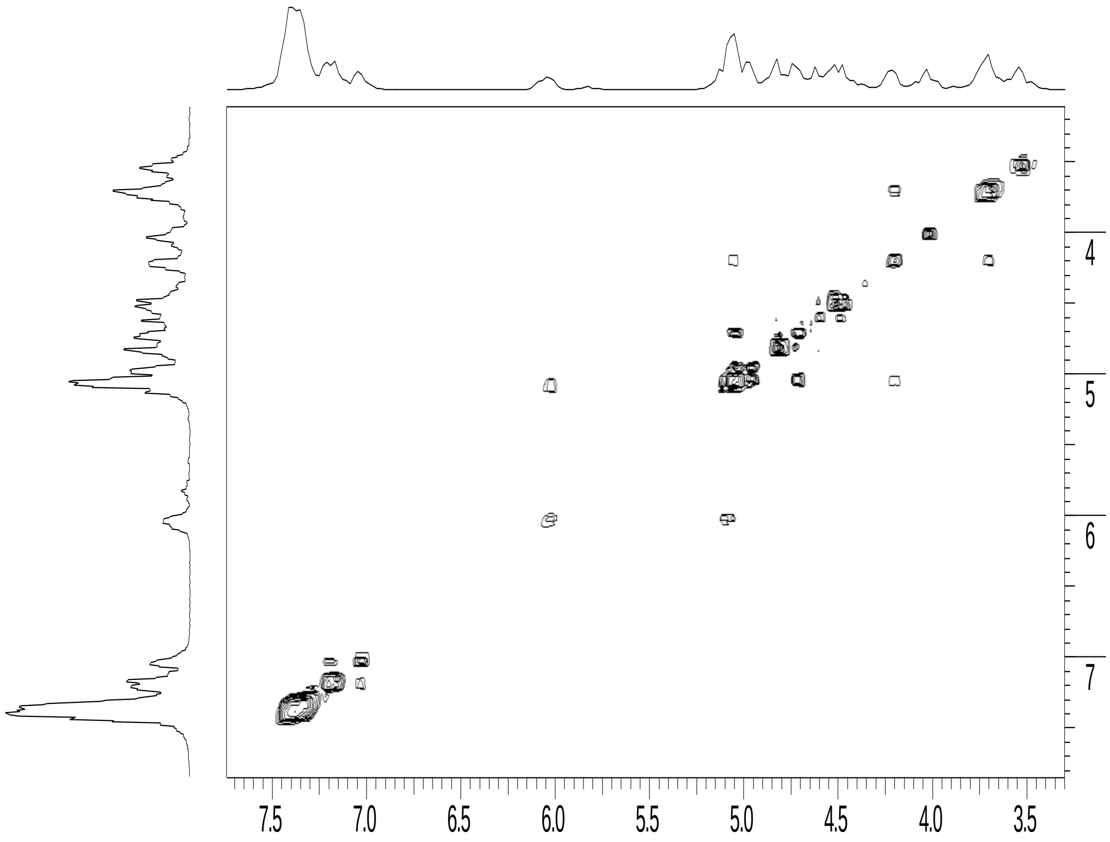

CDCl$_3$ at 300 MHz

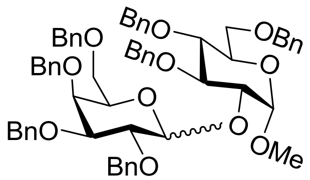

9d

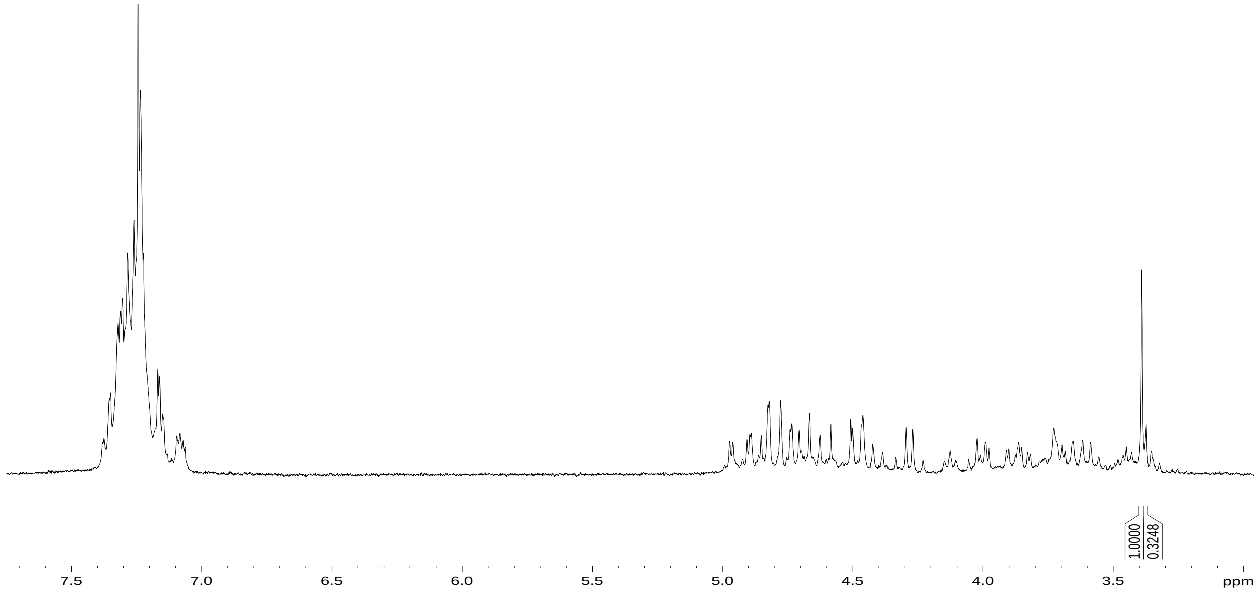

$CDCl_3$ at 300 MHz

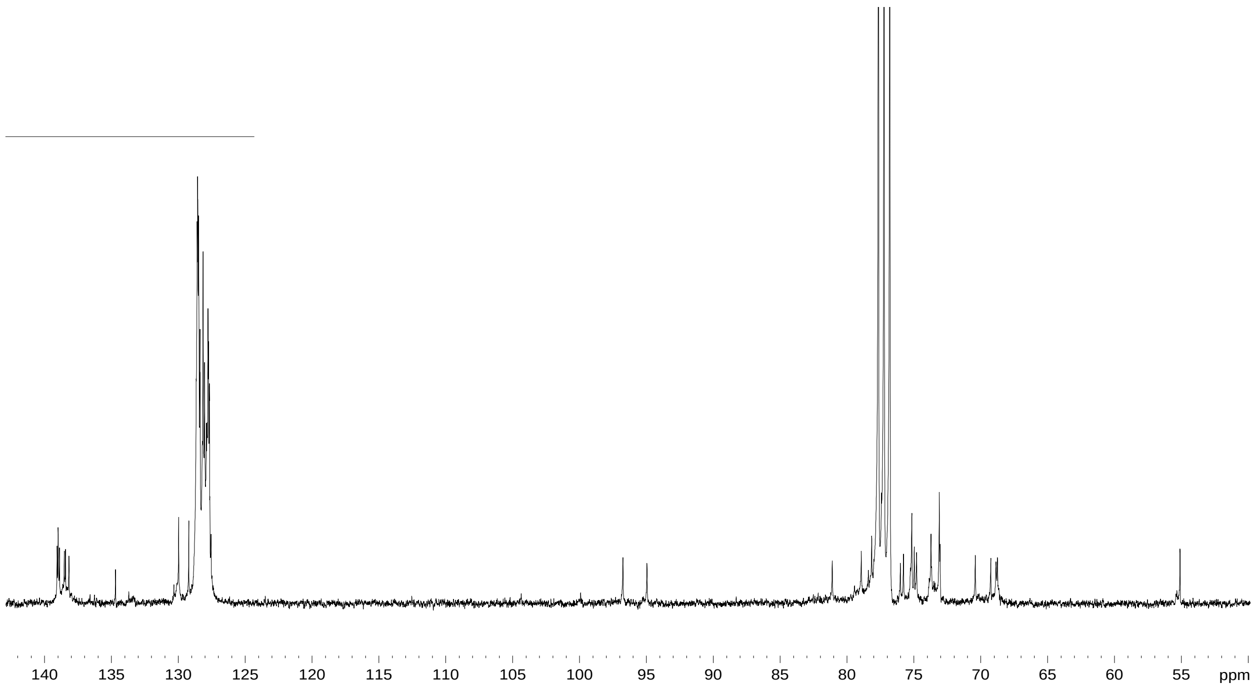

$CDCl_3$ at 75 MHz

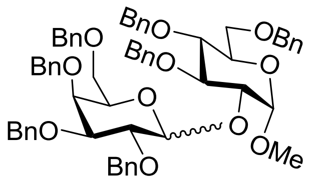

9d

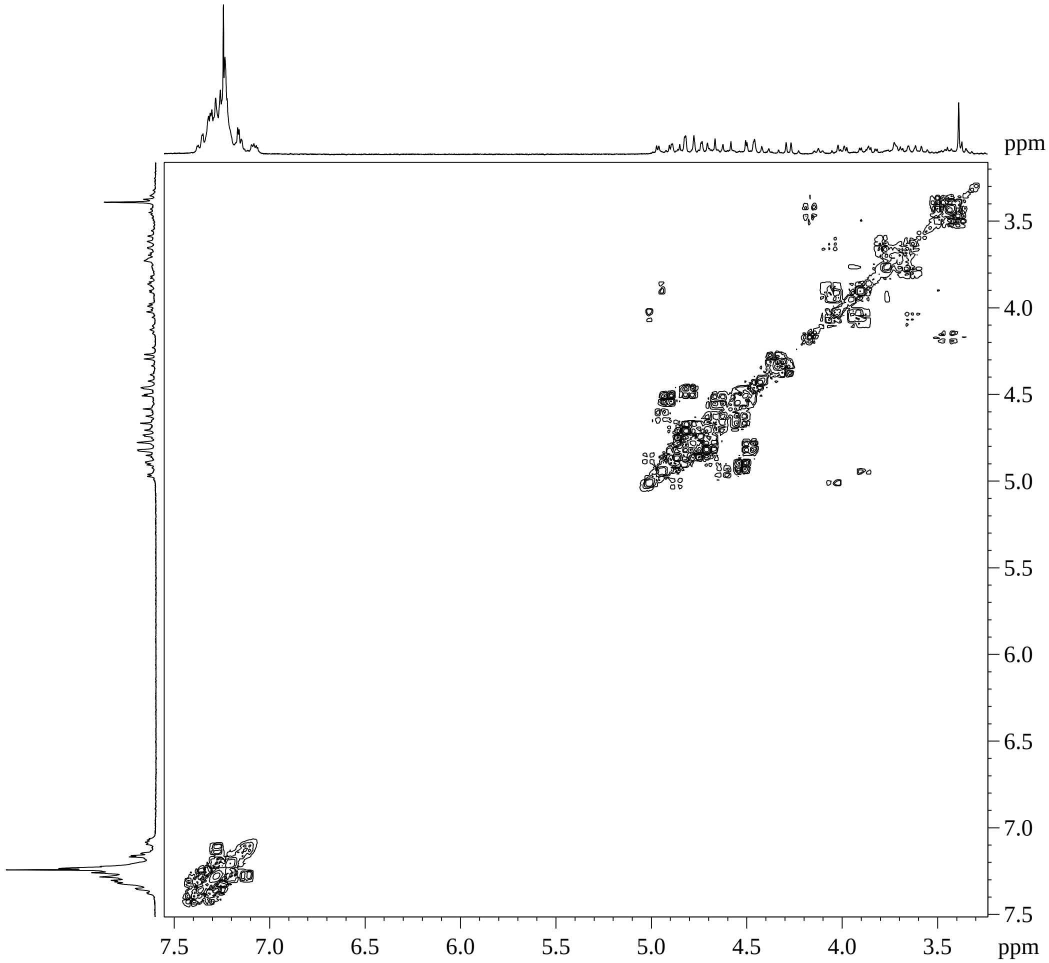

CDCl$_3$ at 300 MHz

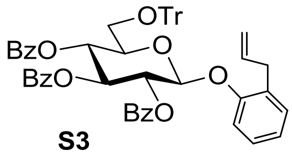

S3

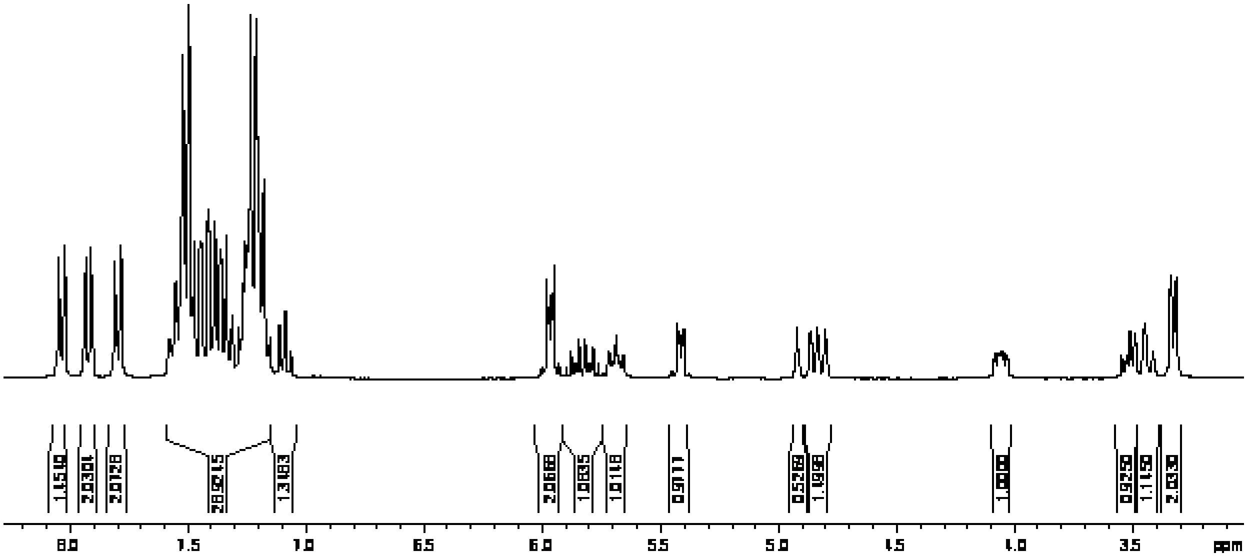

CDCl$_3$ at 300 MHz

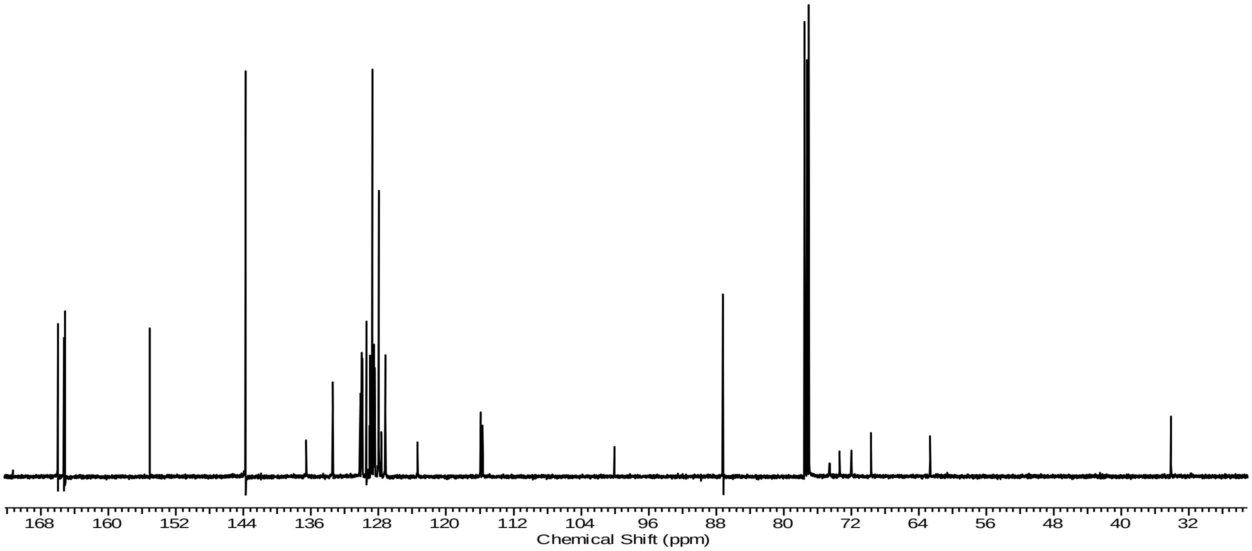

CDCl$_3$ at 75 MHz

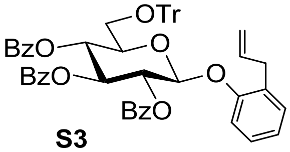

S3

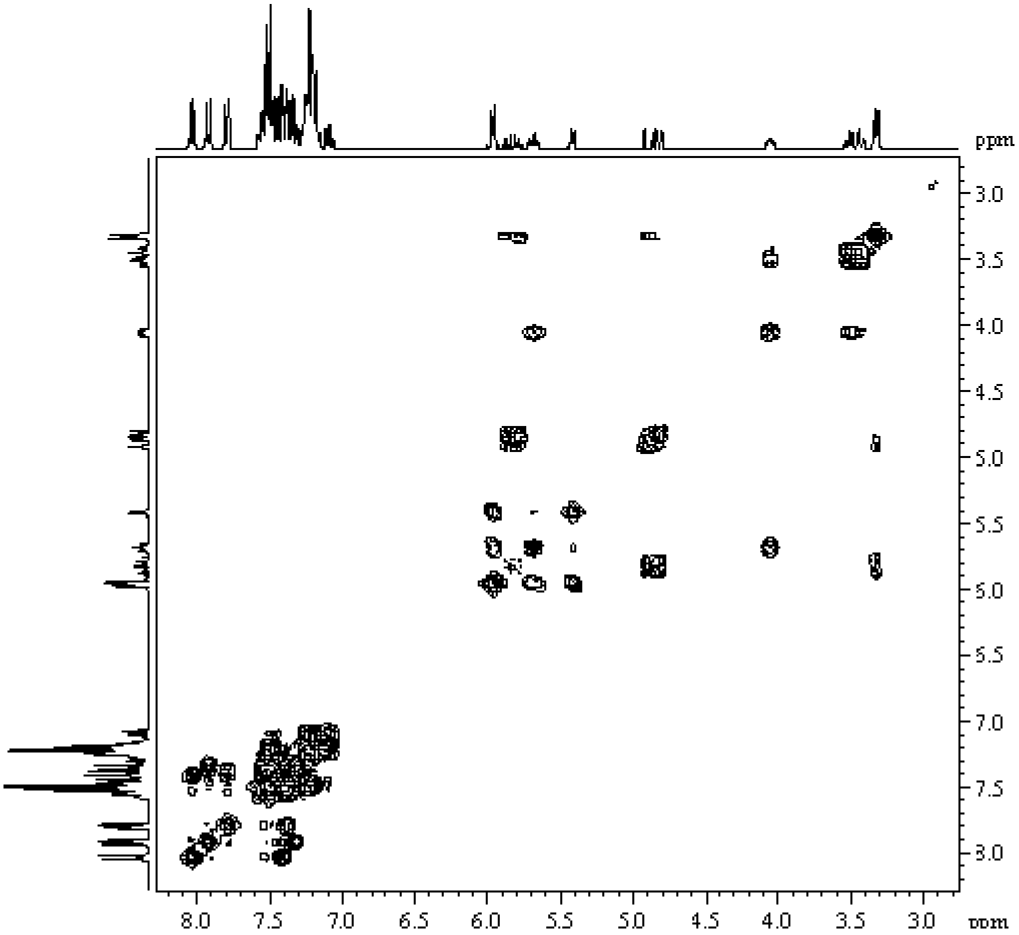

CDCl$_3$ at 300 MHz

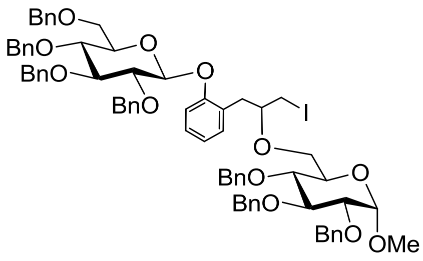

12

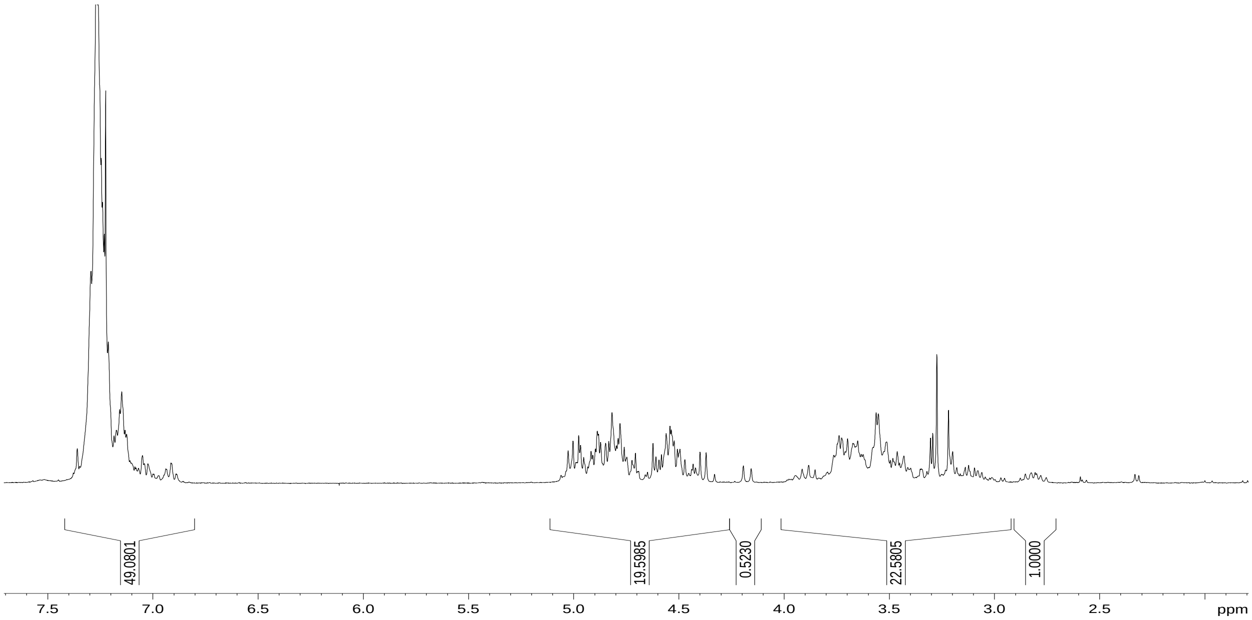

$CDCl_3$ at 300 MHz

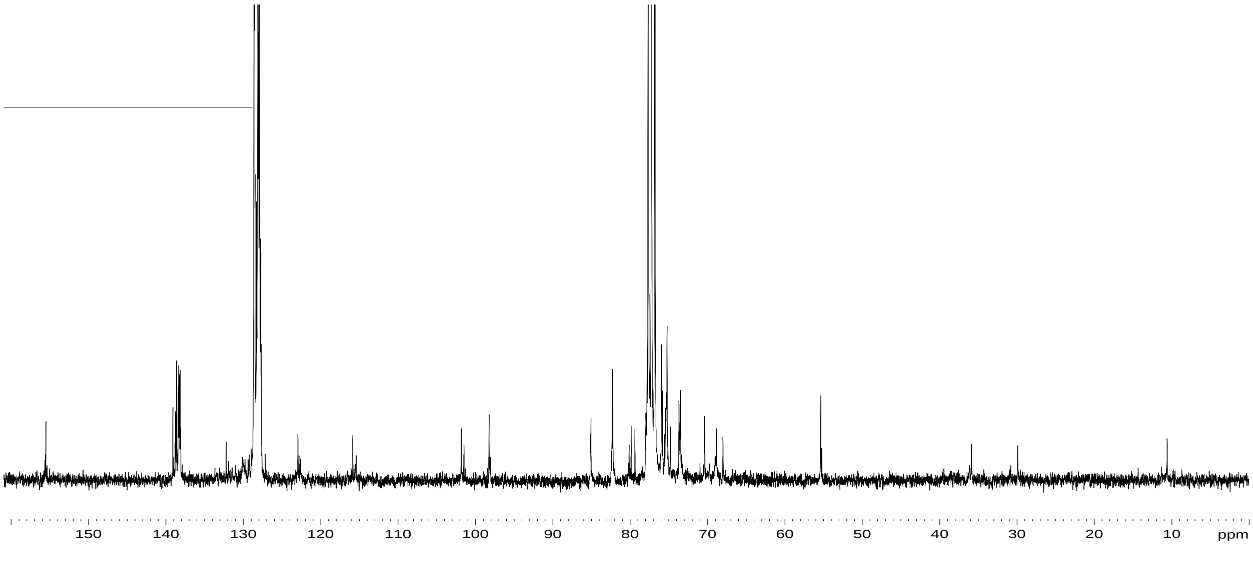

$CDCl_3$ at 75 MHz

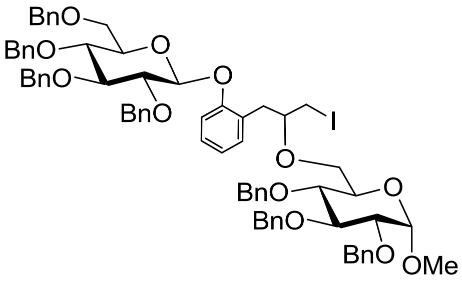

12

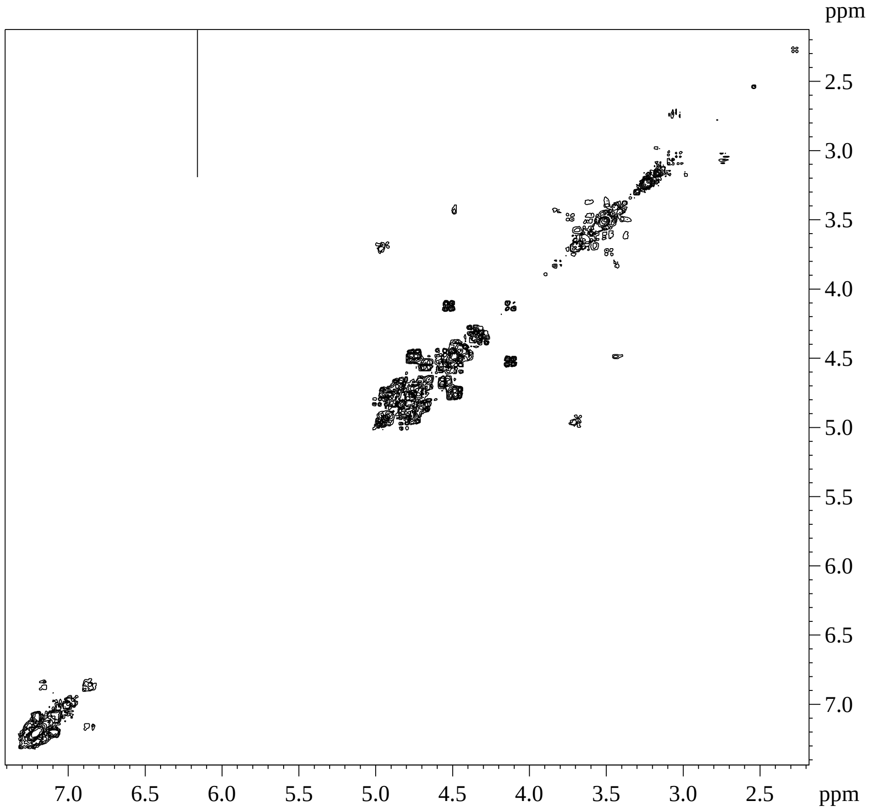

CDCl$_3$ at 300 MHz

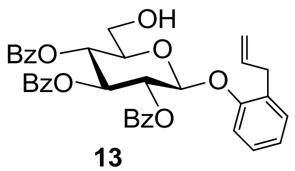

13

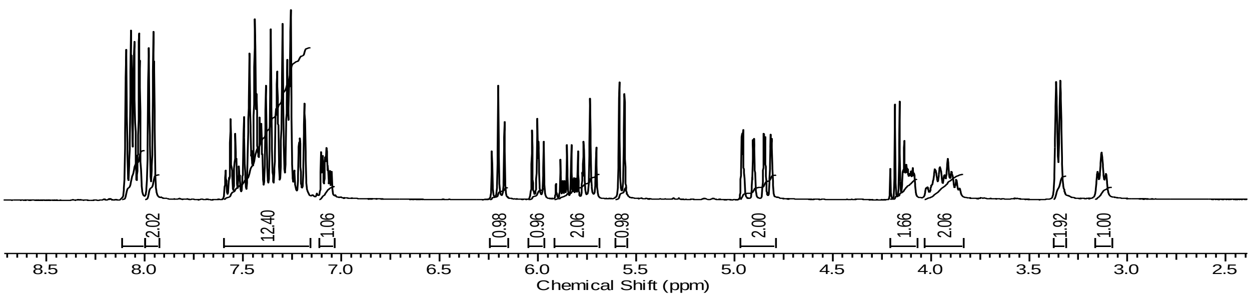

$CDCl_3$ at 300 MHz

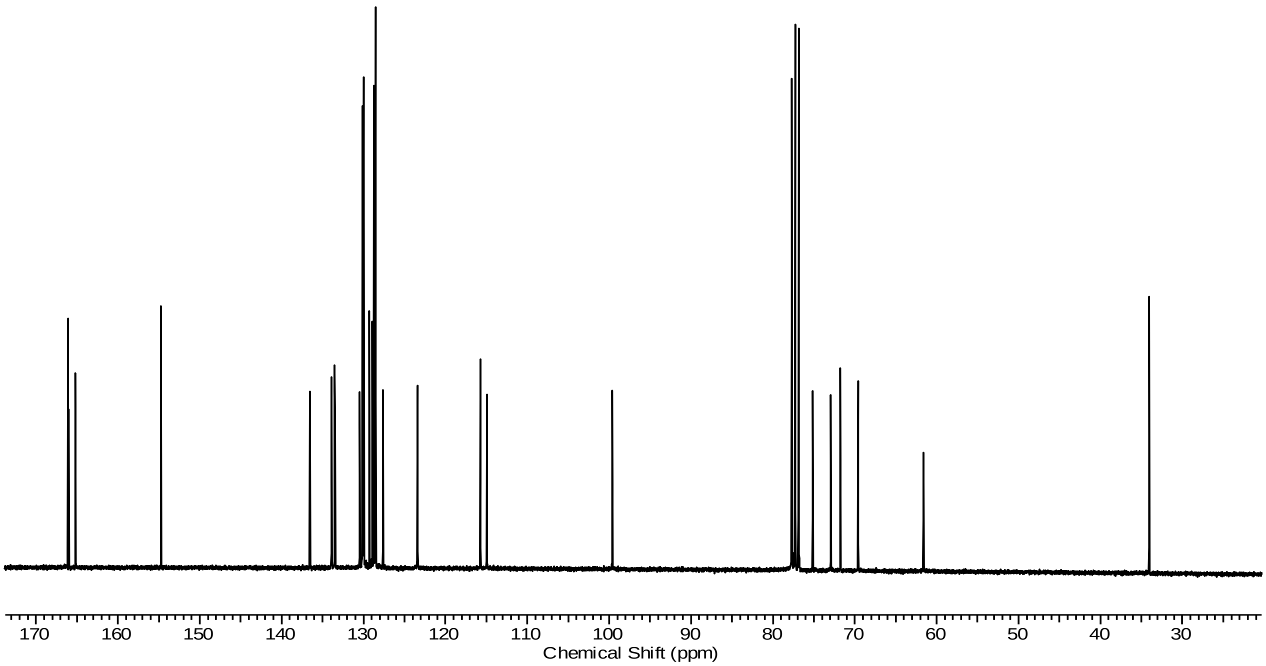

$CDCl_3$ at 75 MHz

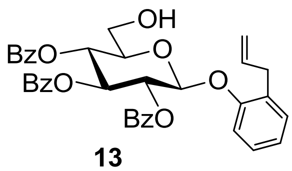

13

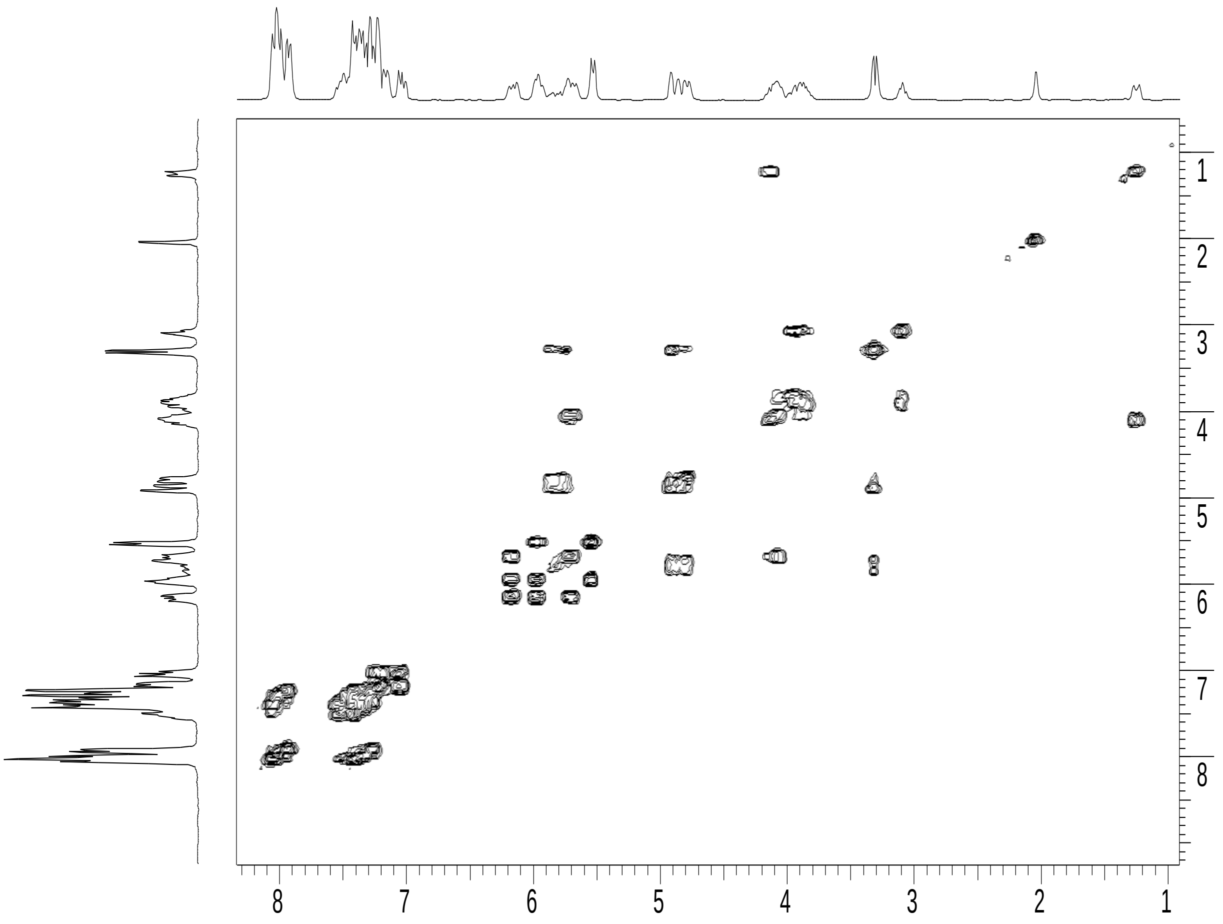

CDCl$_3$ at 300 MHz

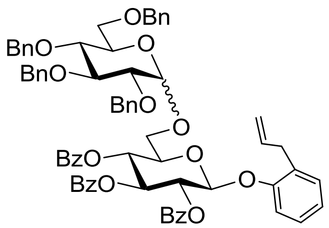

14

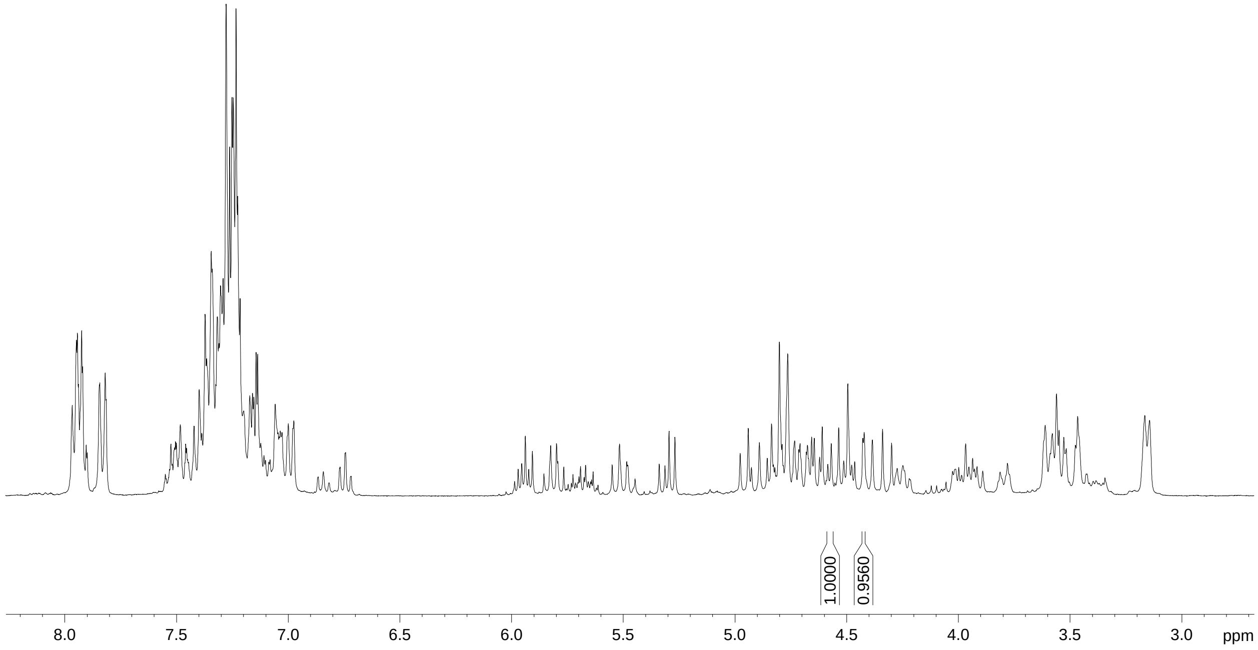

$CDCl_3$ at 300 MHz

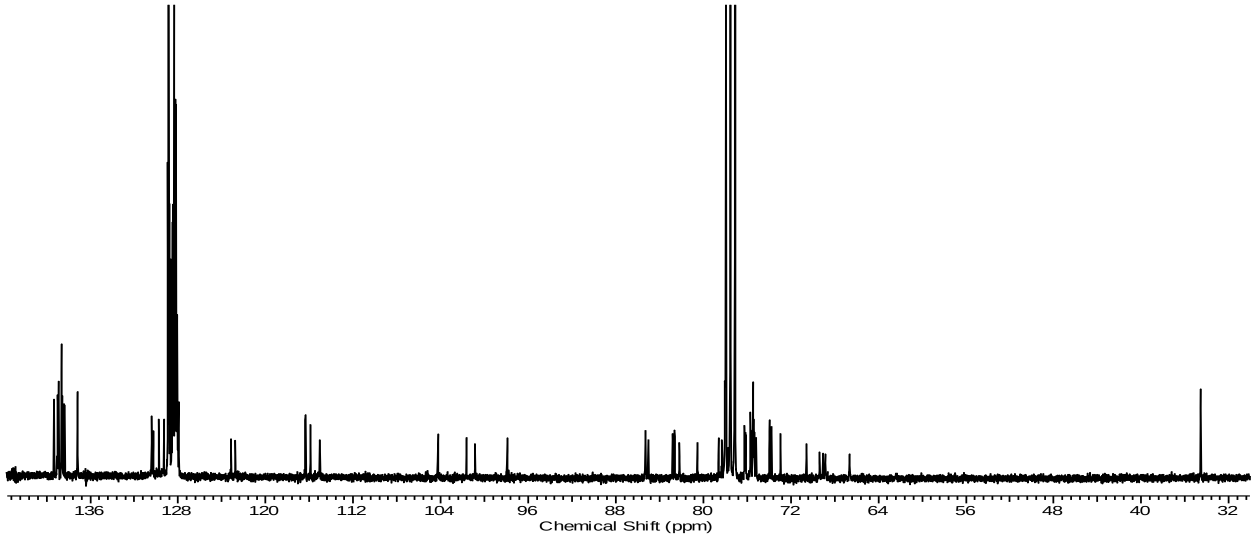

$CDCl_3$ at 75 MHz

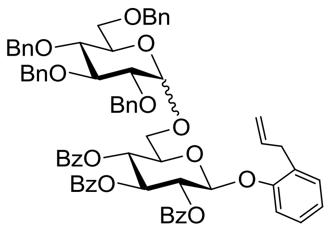

14

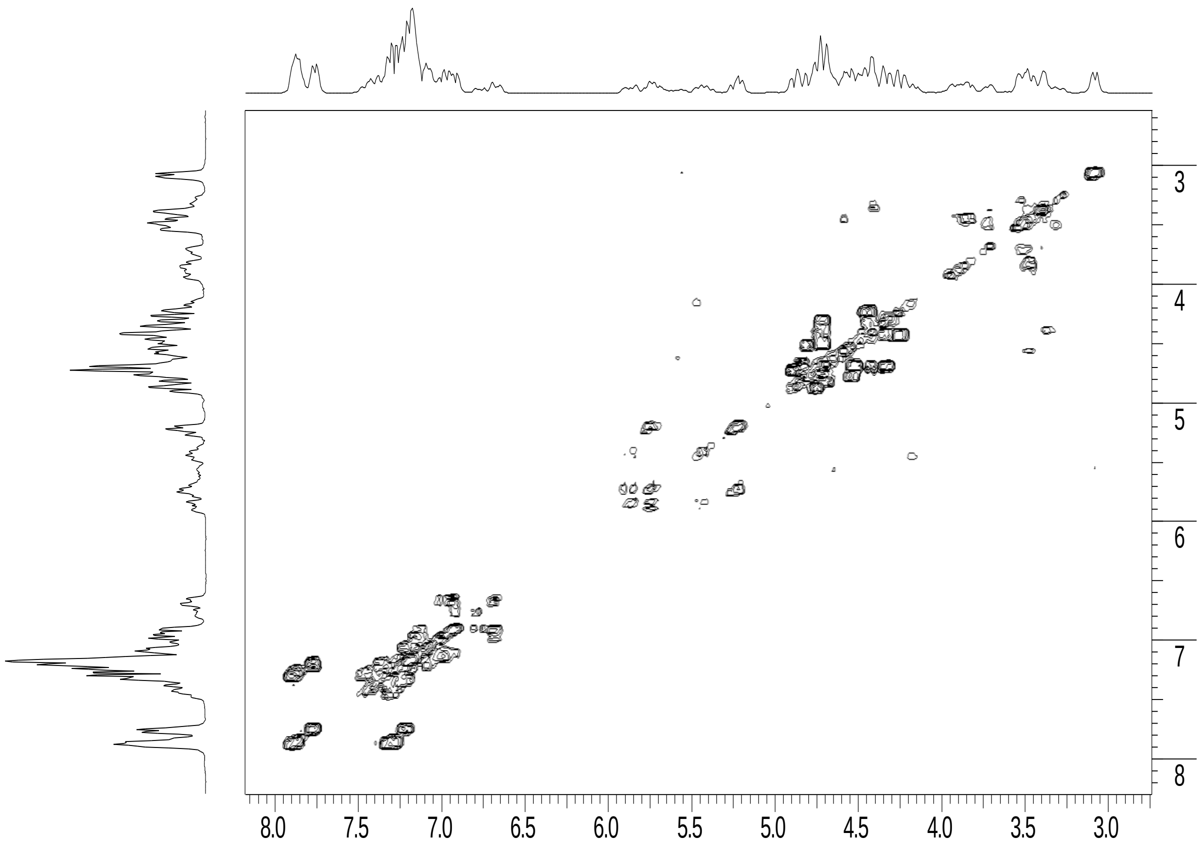

$CDCl_3$ at 300 MHz

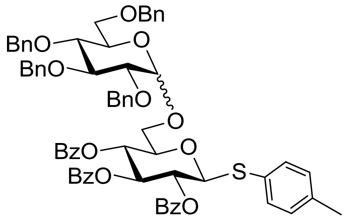

18

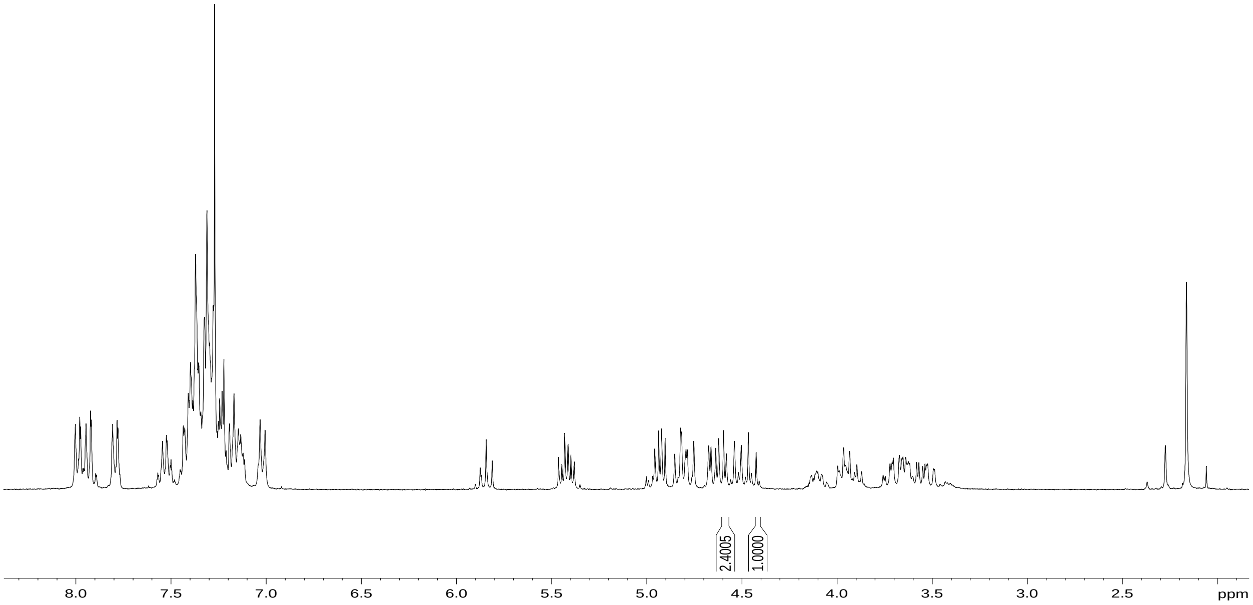

CDCl$_3$ at 300 MHz

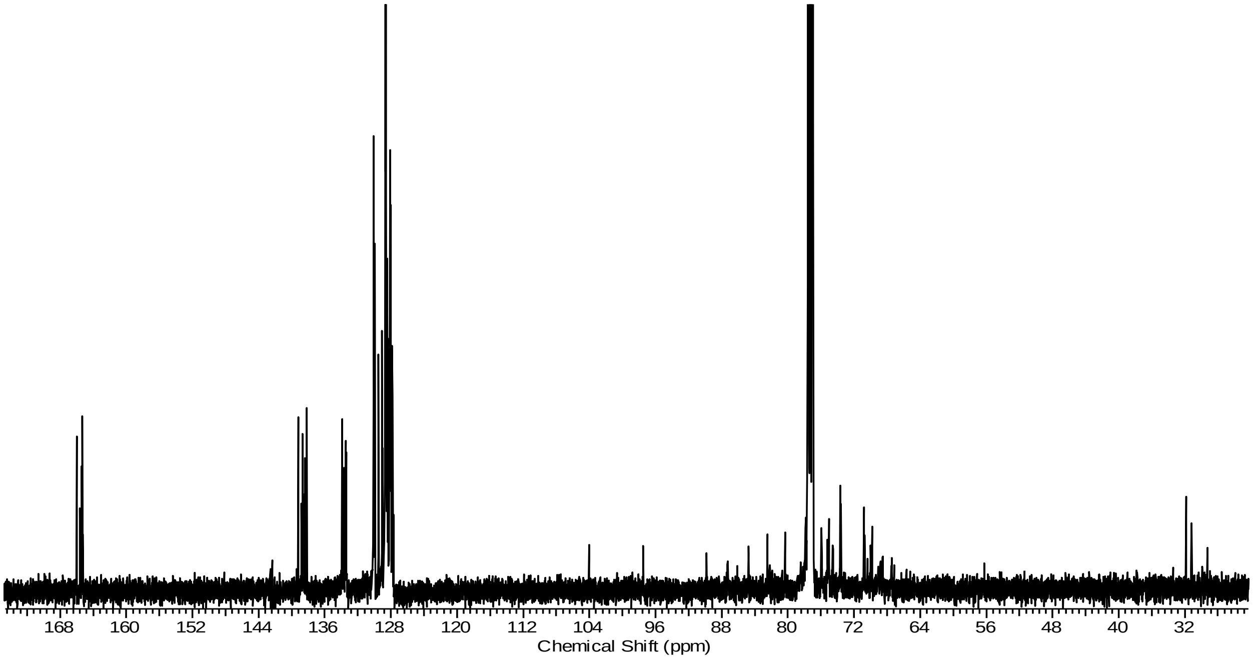

CDCl$_3$ at 75 MHz

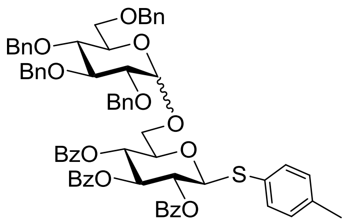

18

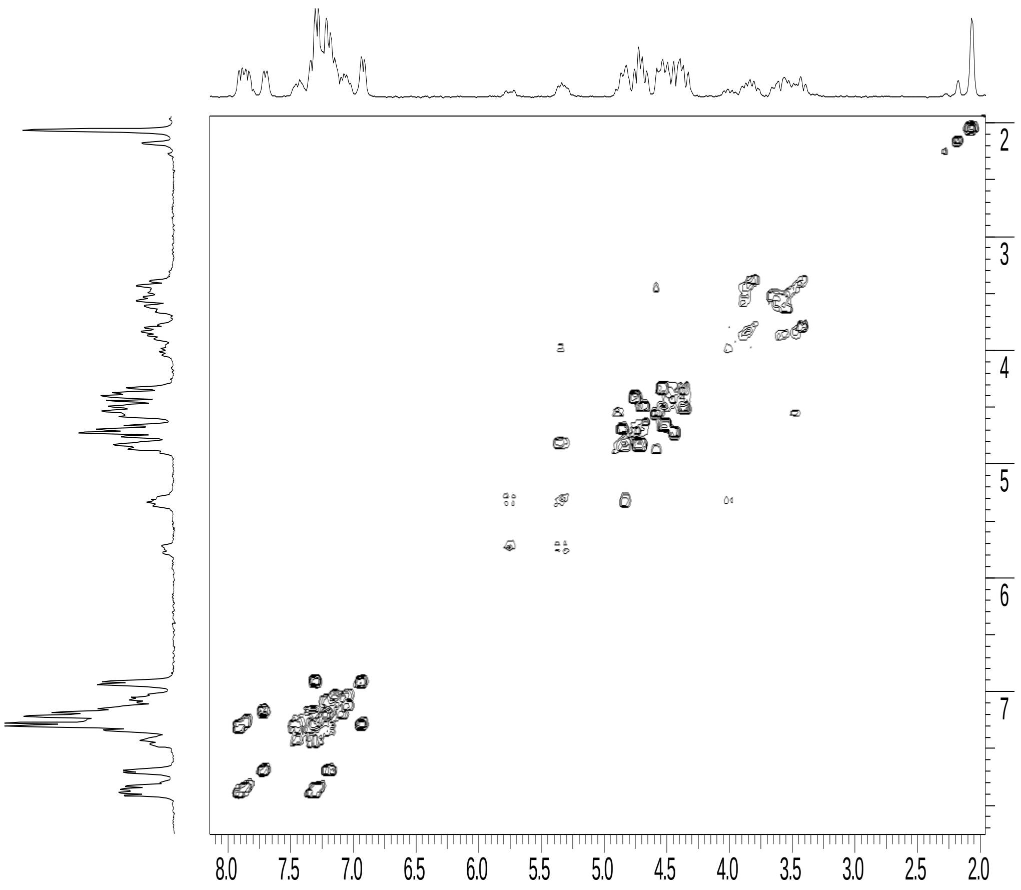

$CDCl_3$ at 300 MHz

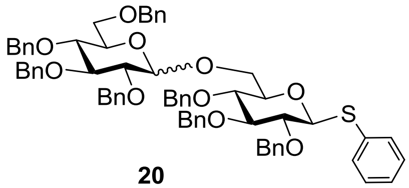

20

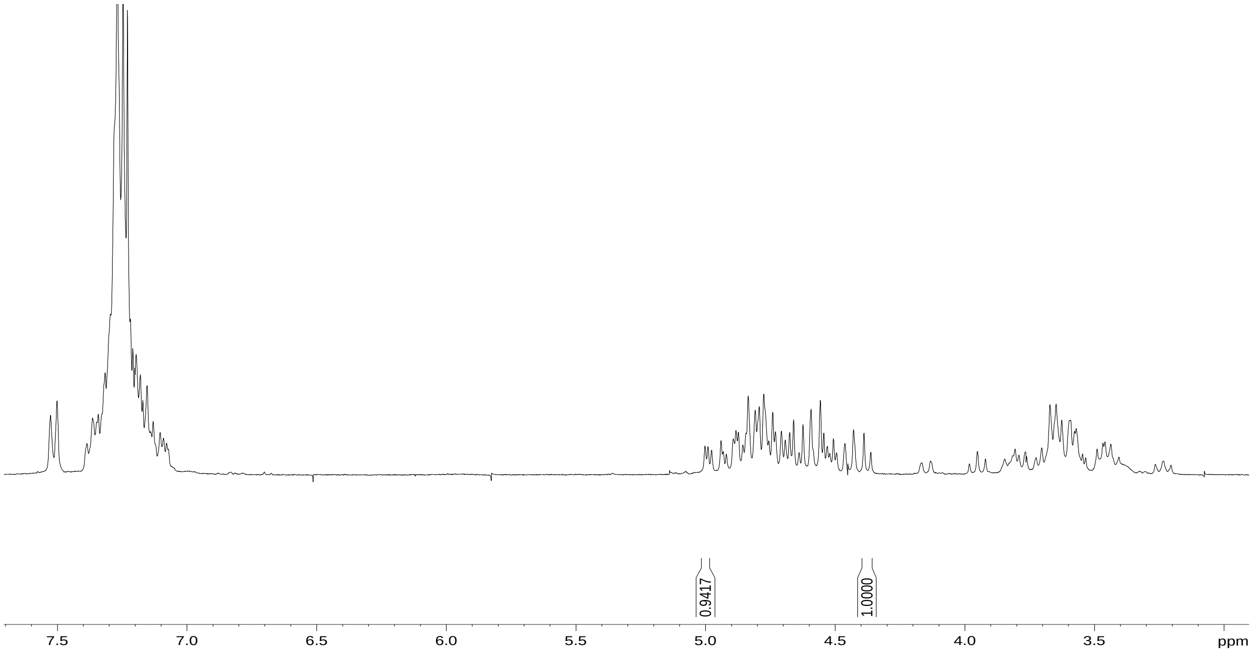

CDCl$_3$ at 300 MHz

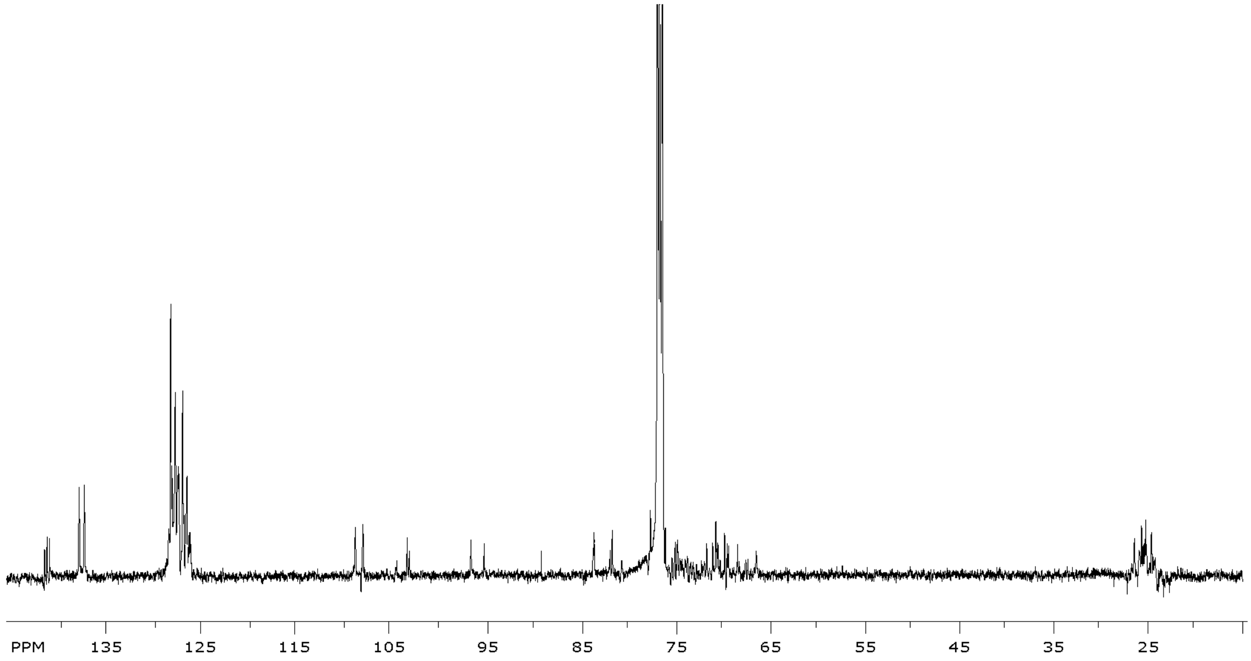

CDCl$_3$ at 125 MHz

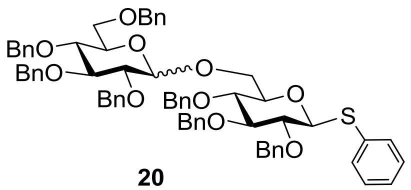

20

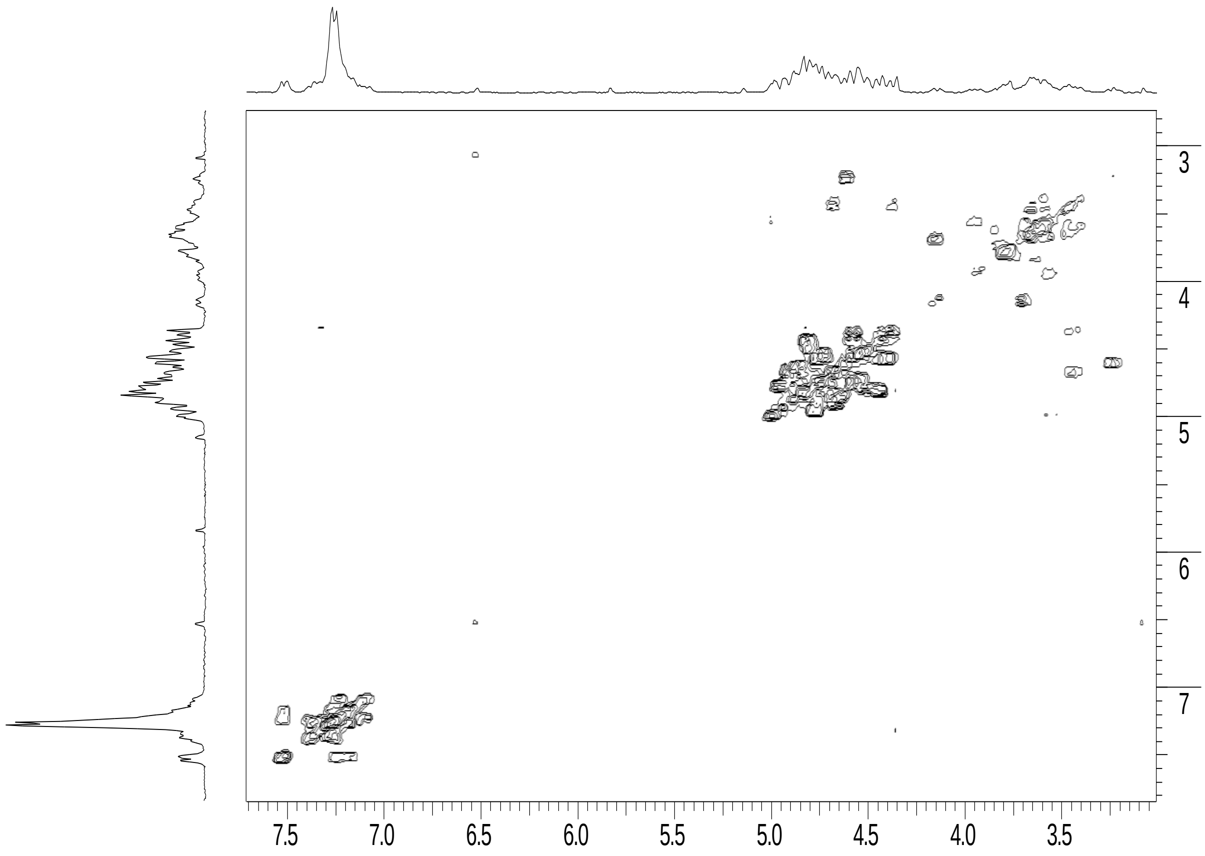

$CDCl_3$ at 300 MHz

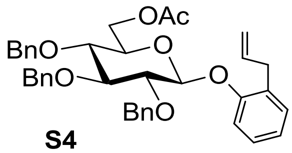

S4

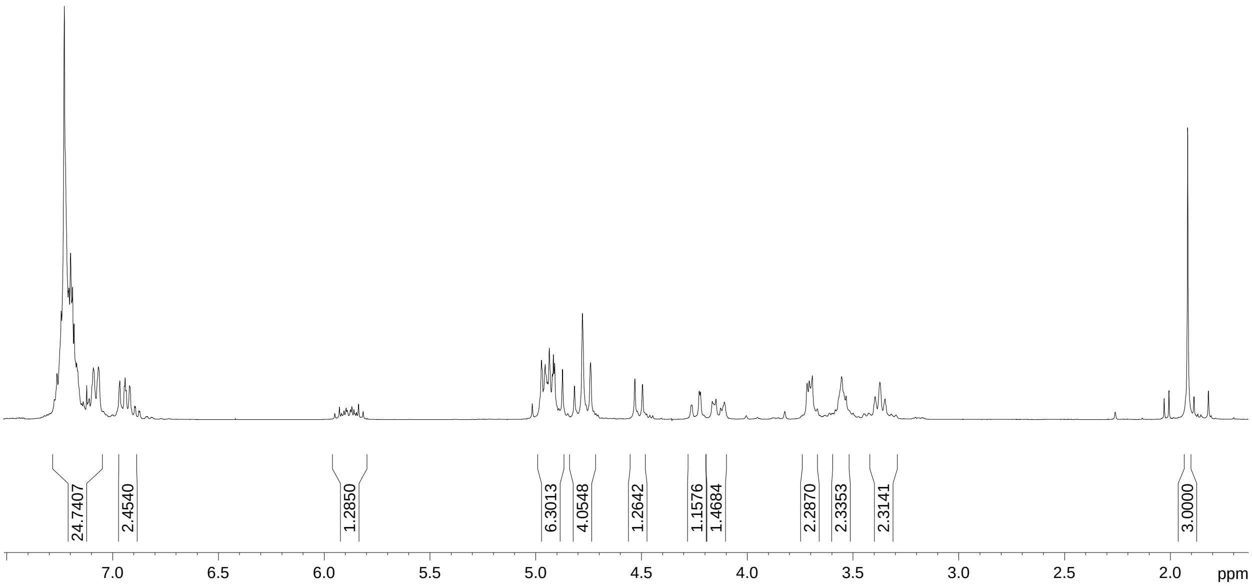

$CDCl_3$ at 300 MHz

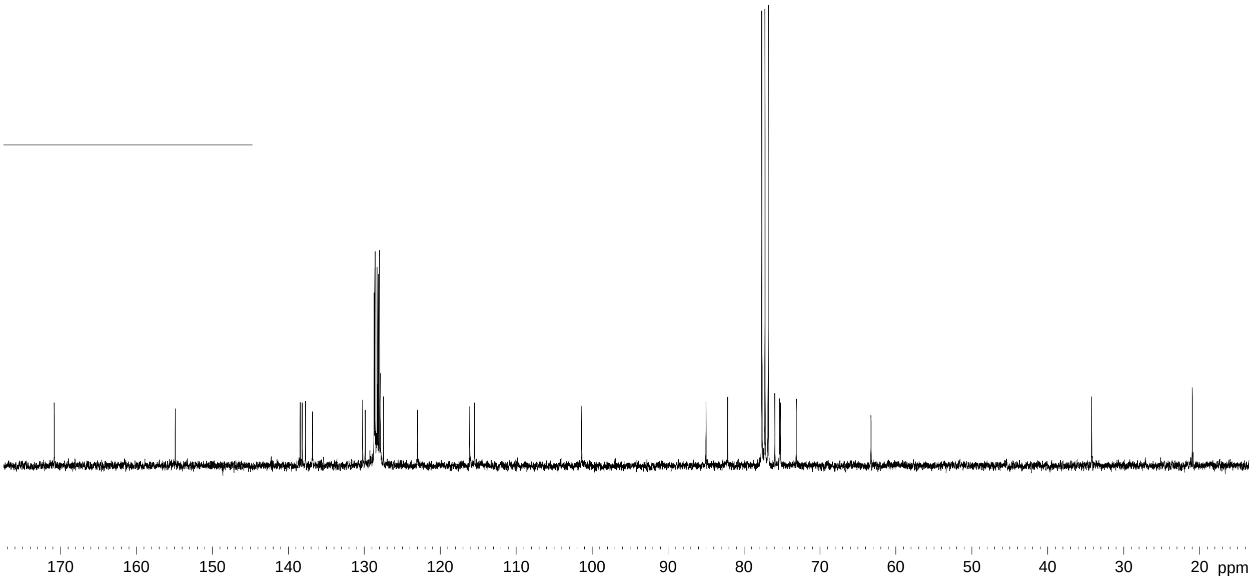

$CDCl_3$ at 75 MHz

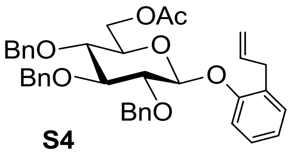

S4

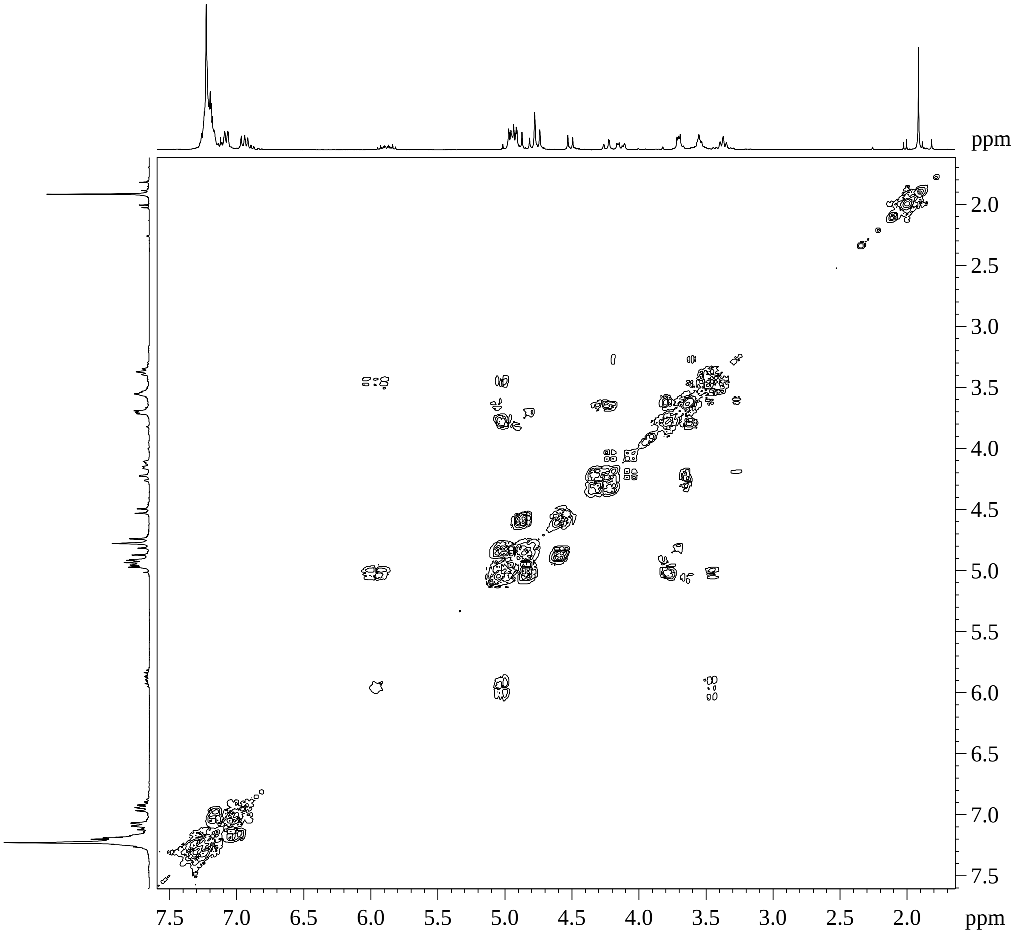

$CDCl_3$ at 300 MHz

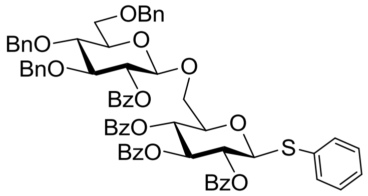

23

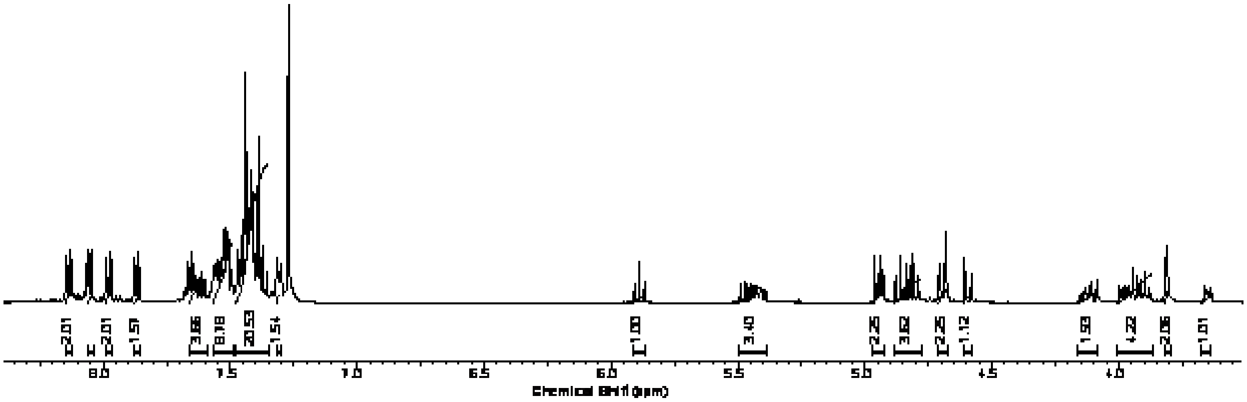

CDCl$_3$ at 300 MHz

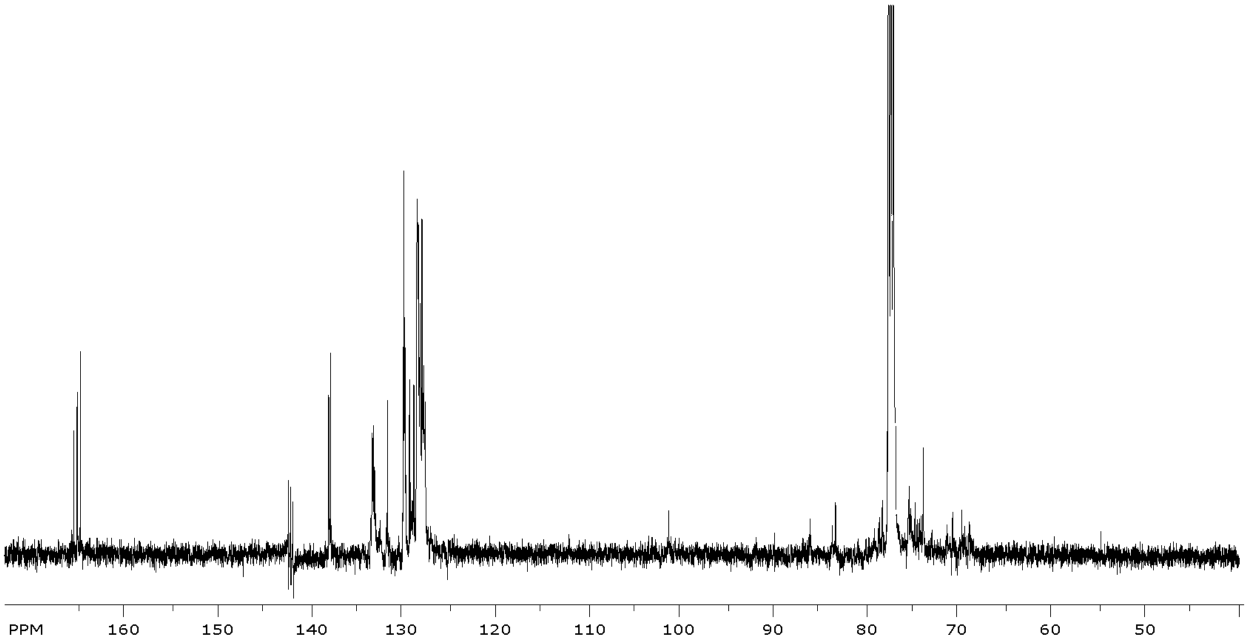

CDCl$_3$ at 125 MHz

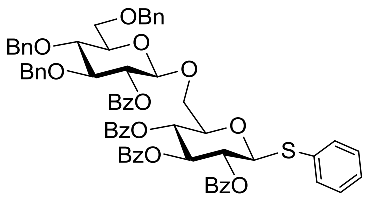

23

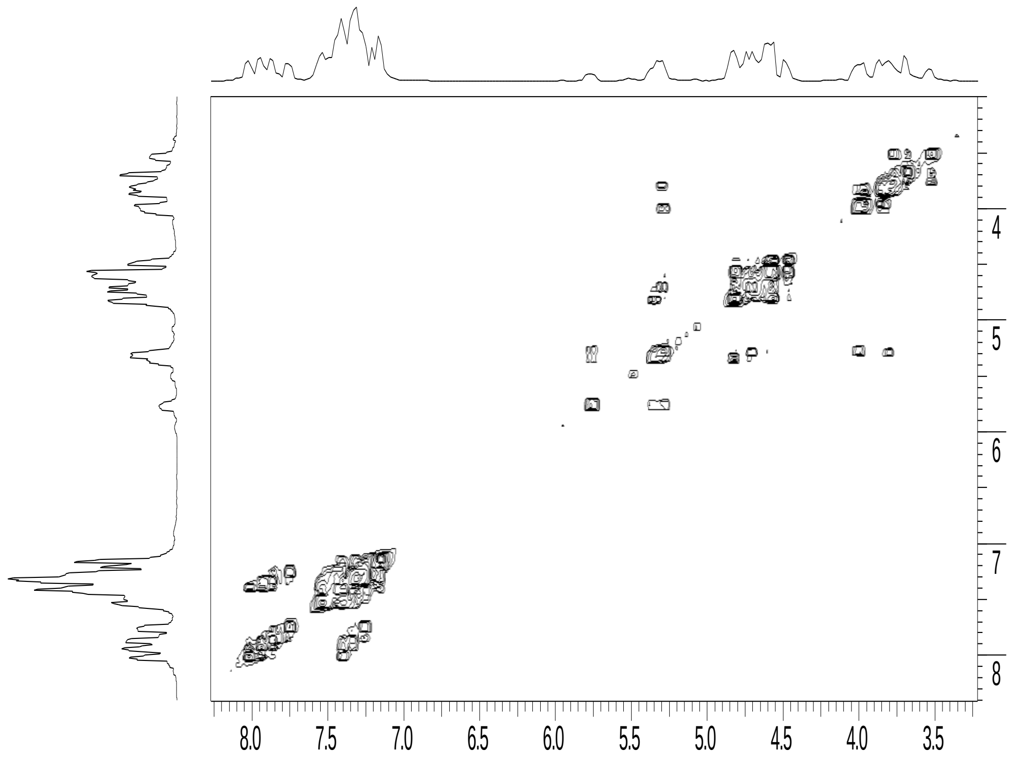

$CDCl_3$ at 300 MHz

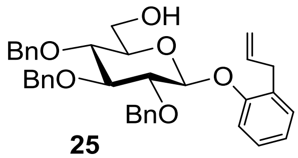

25

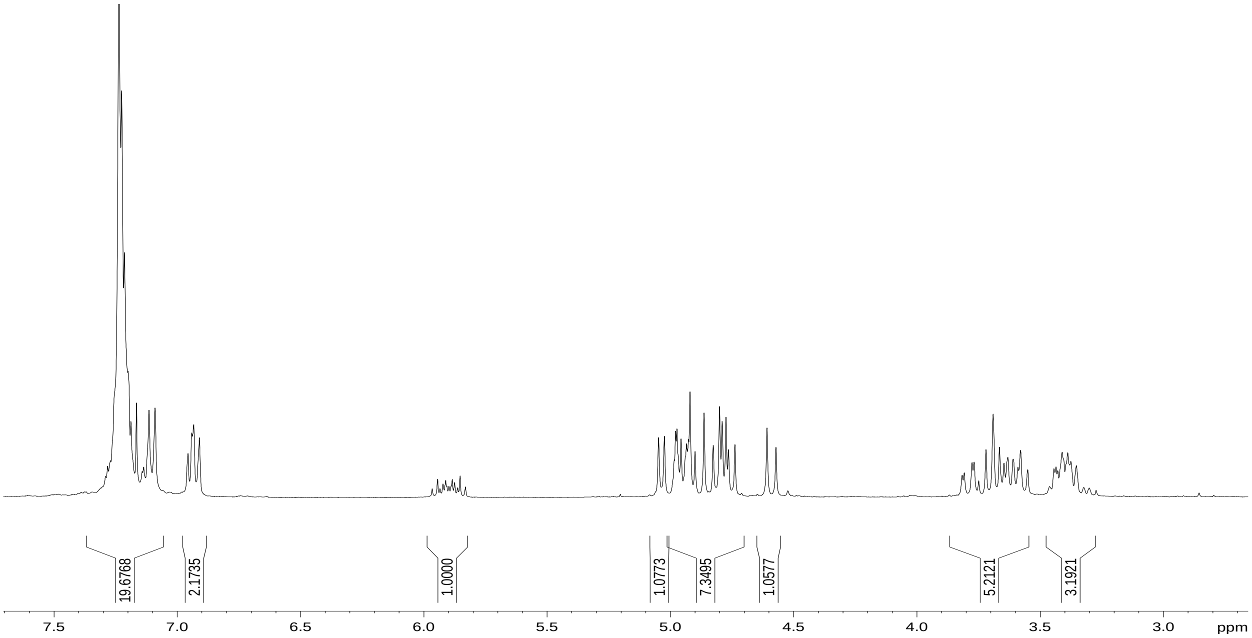

$CDCl_3$ at 300 MHz

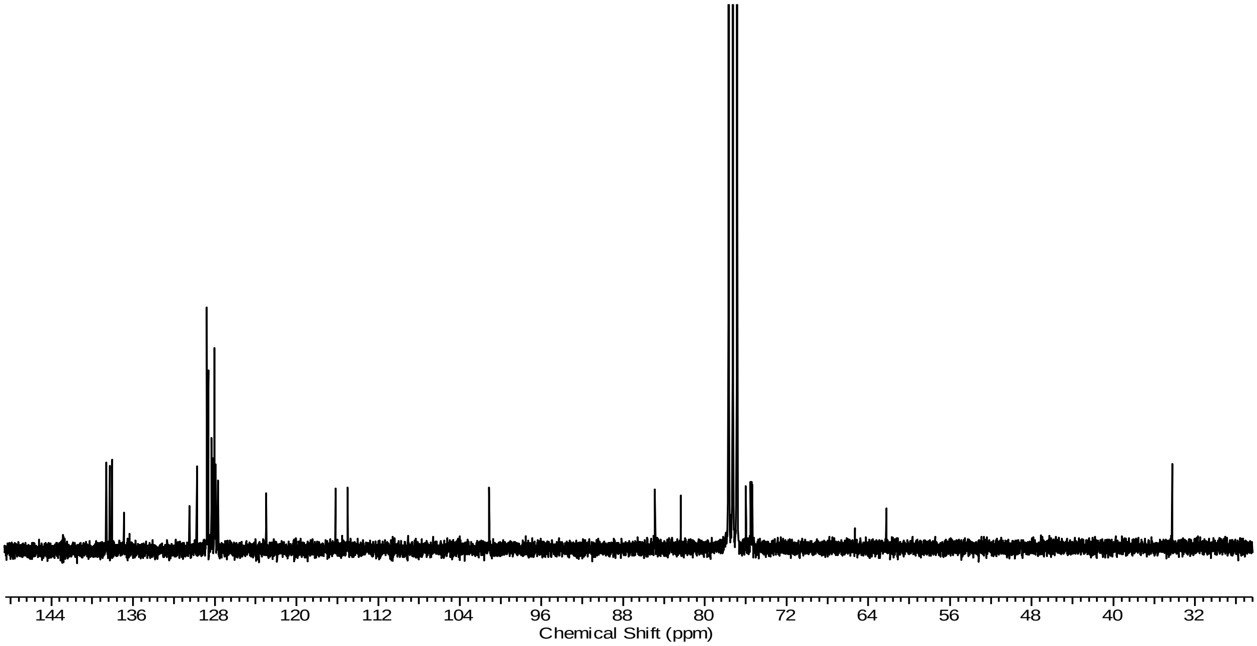

$CDCl_3$ at 75 MHz

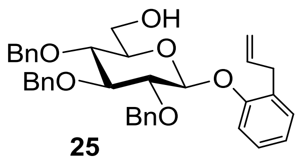

25

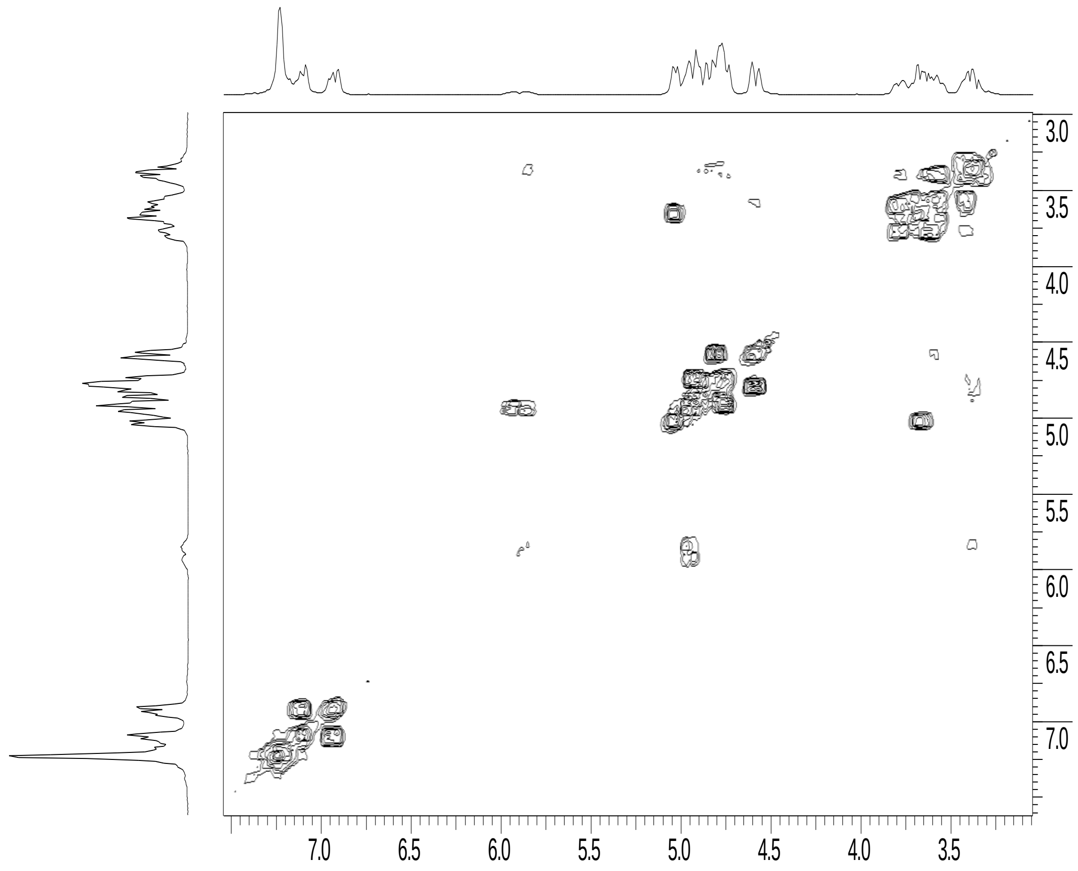

CDCl$_3$ at 300 MHz

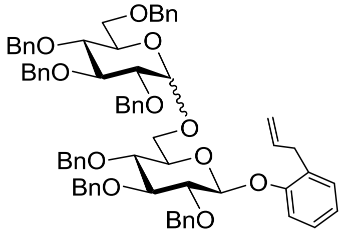

26

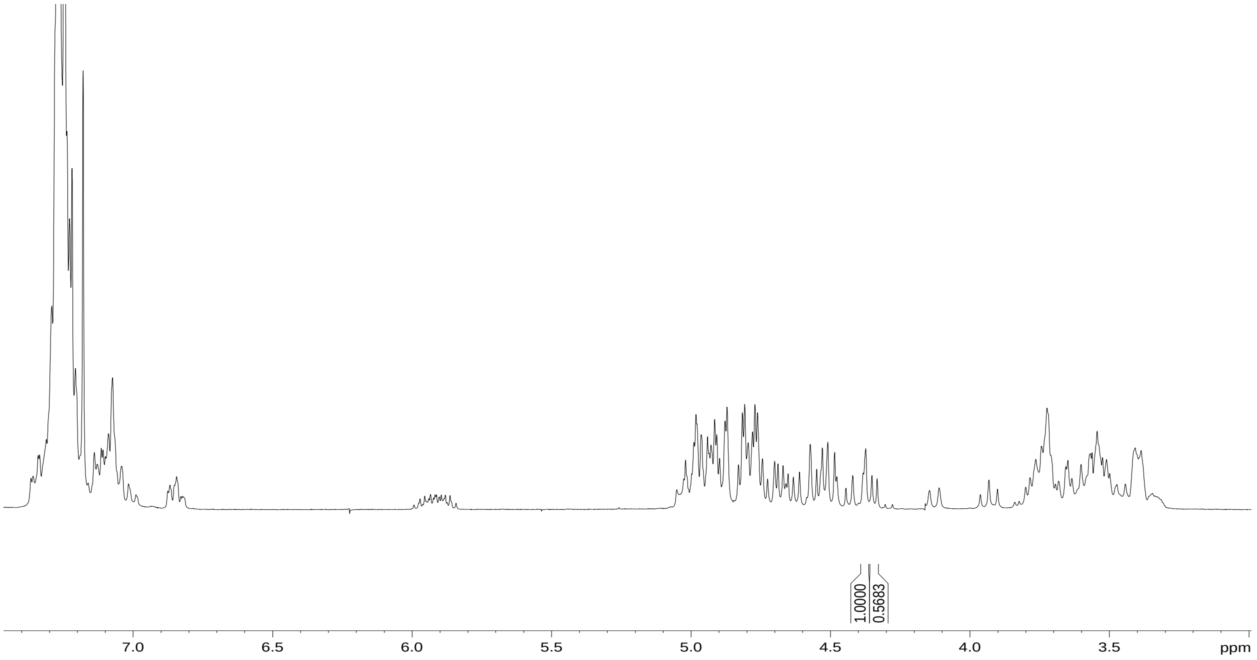

$CDCl_3$ at 300 MHz

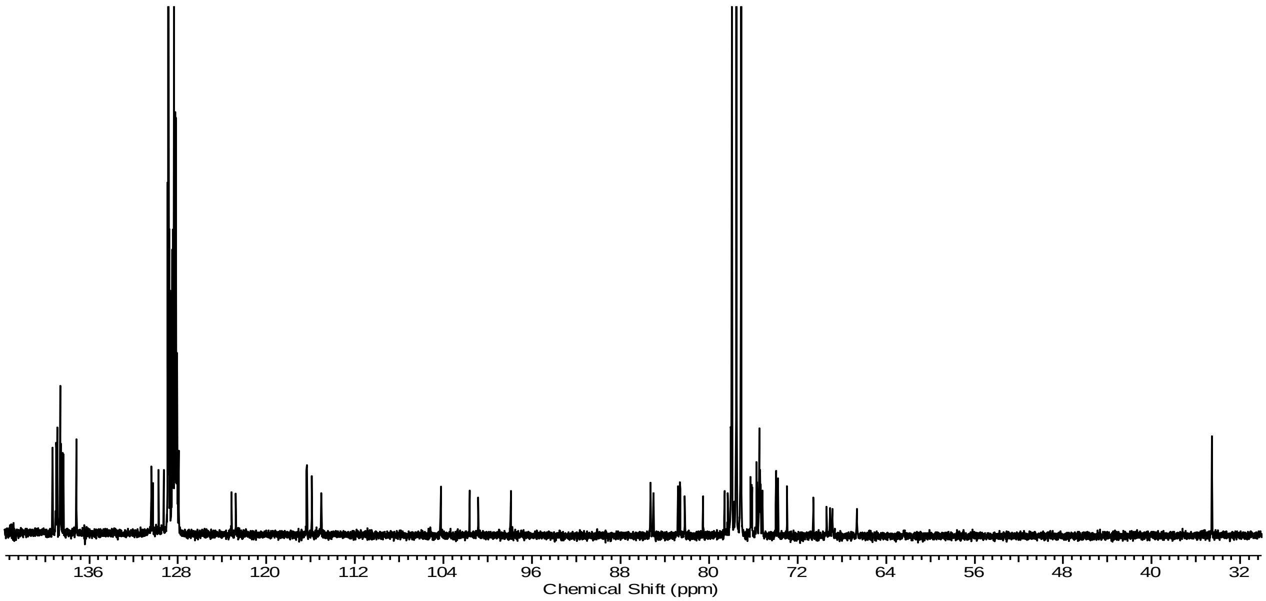

$CDCl_3$ at 75 MHz

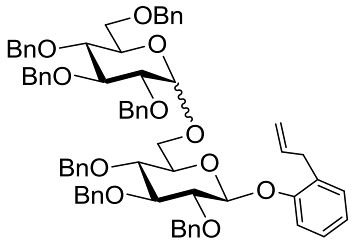

26

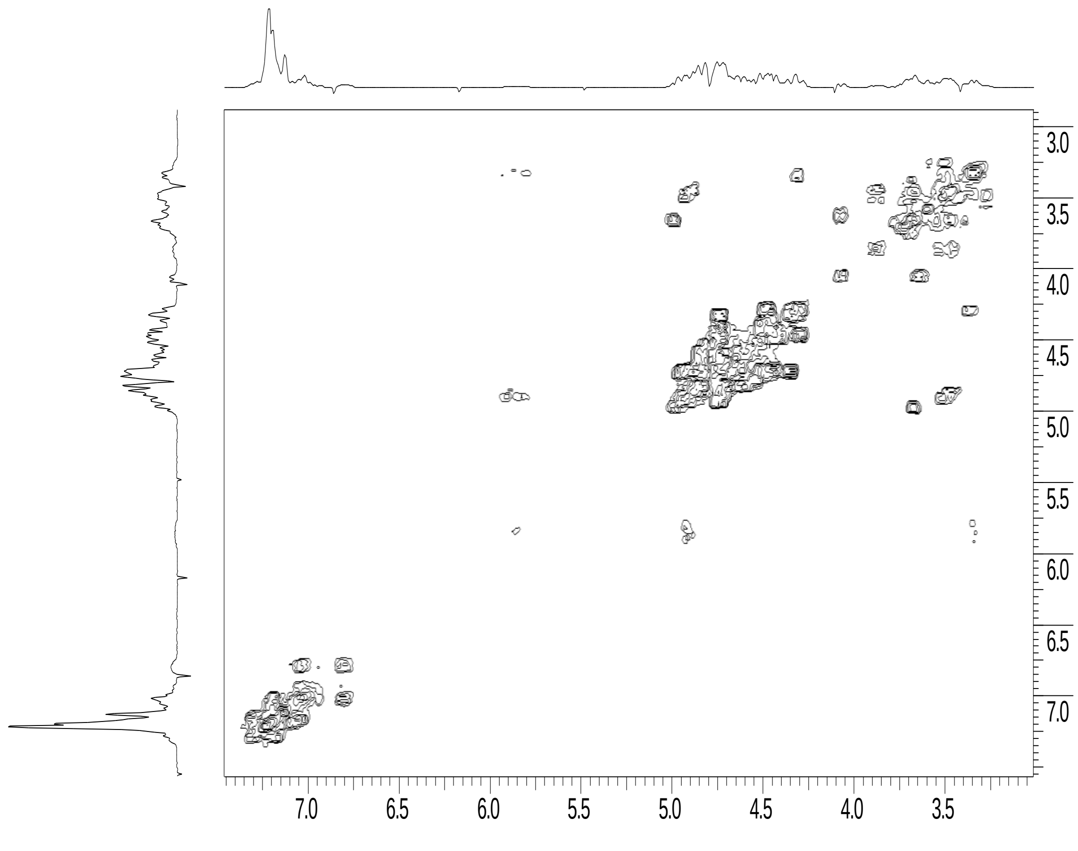

$CDCl_3$ at 300 MHz

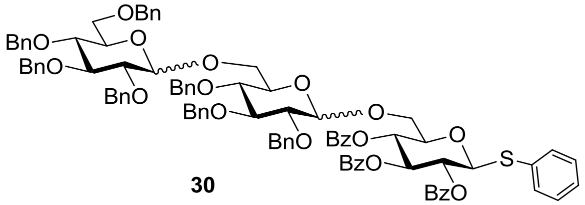

30

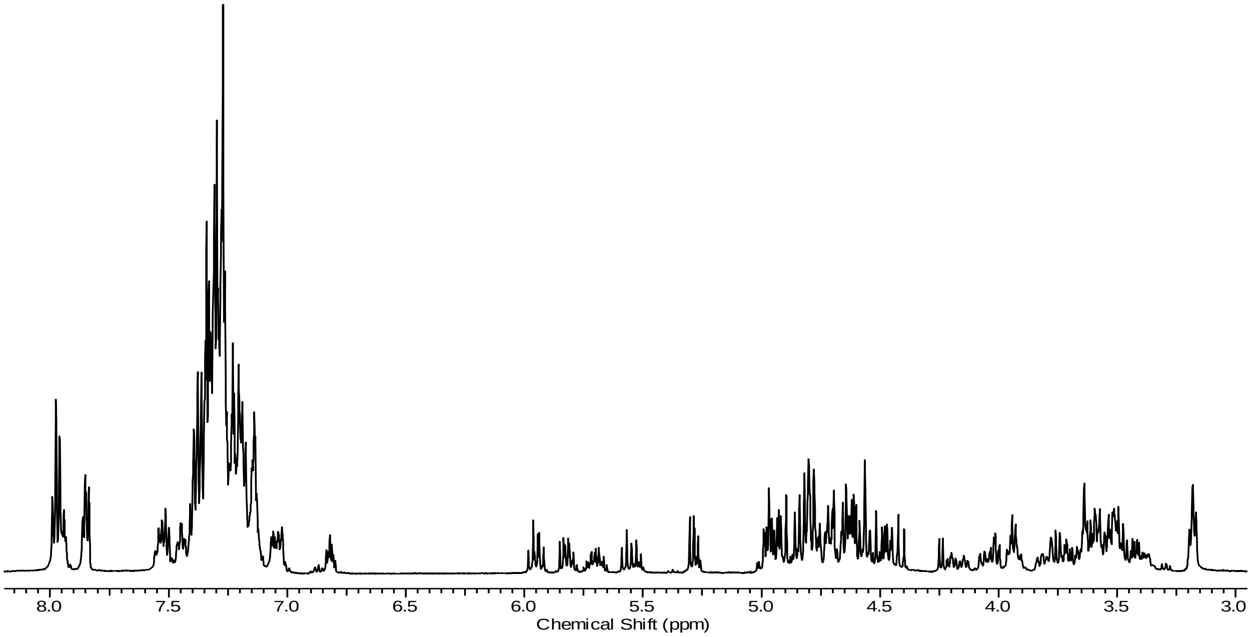

$CDCl_3$ at 300 MHz

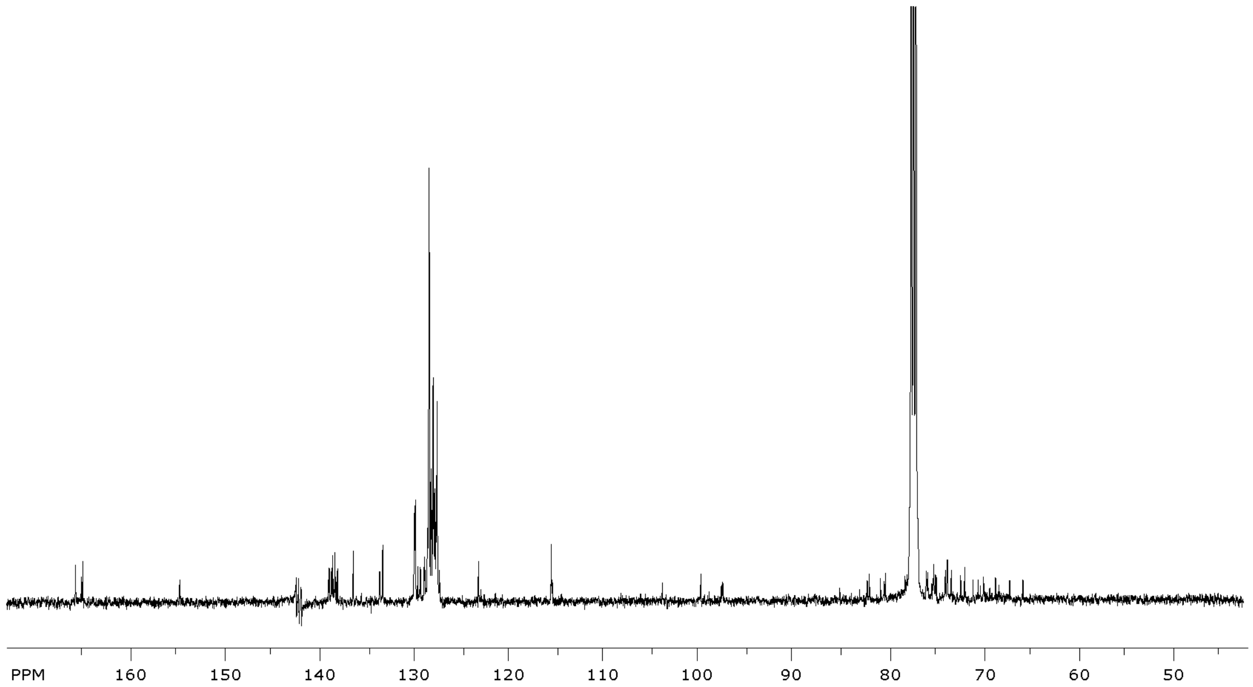

$CDCl_3$ at 125 MHz

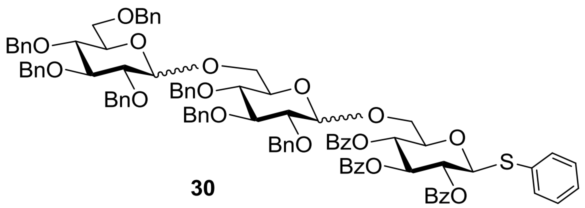

30

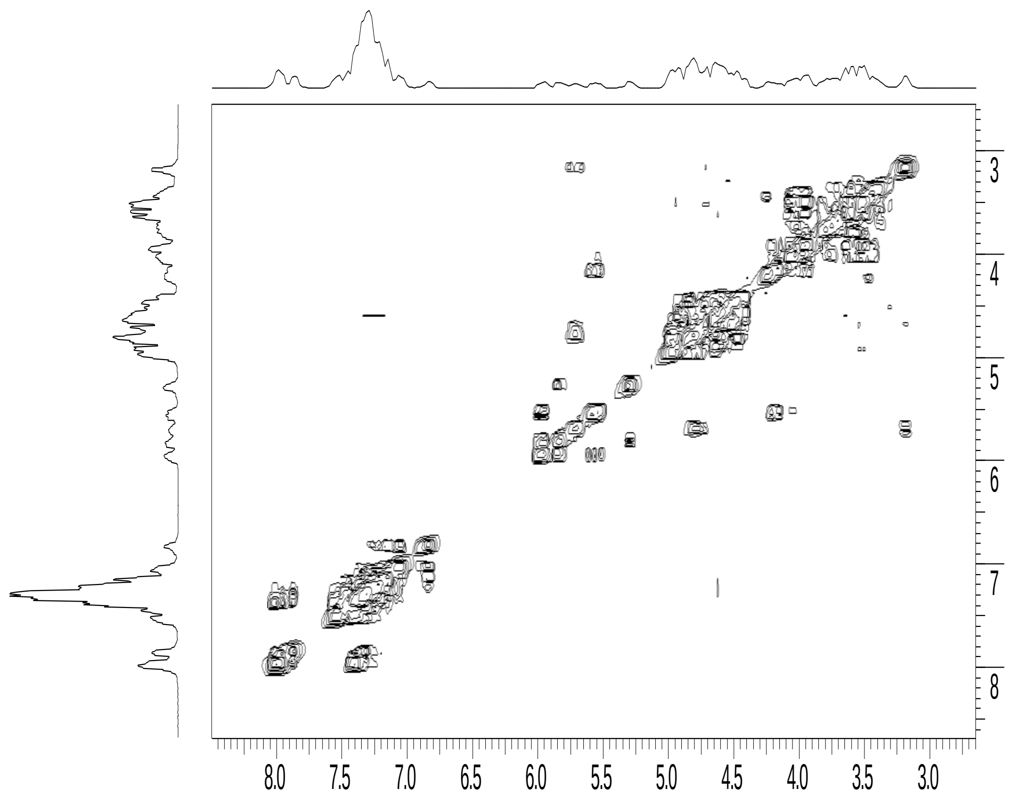

$CDCl_3$ at 300 MHz

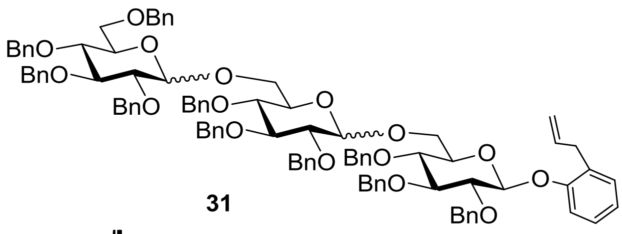

31

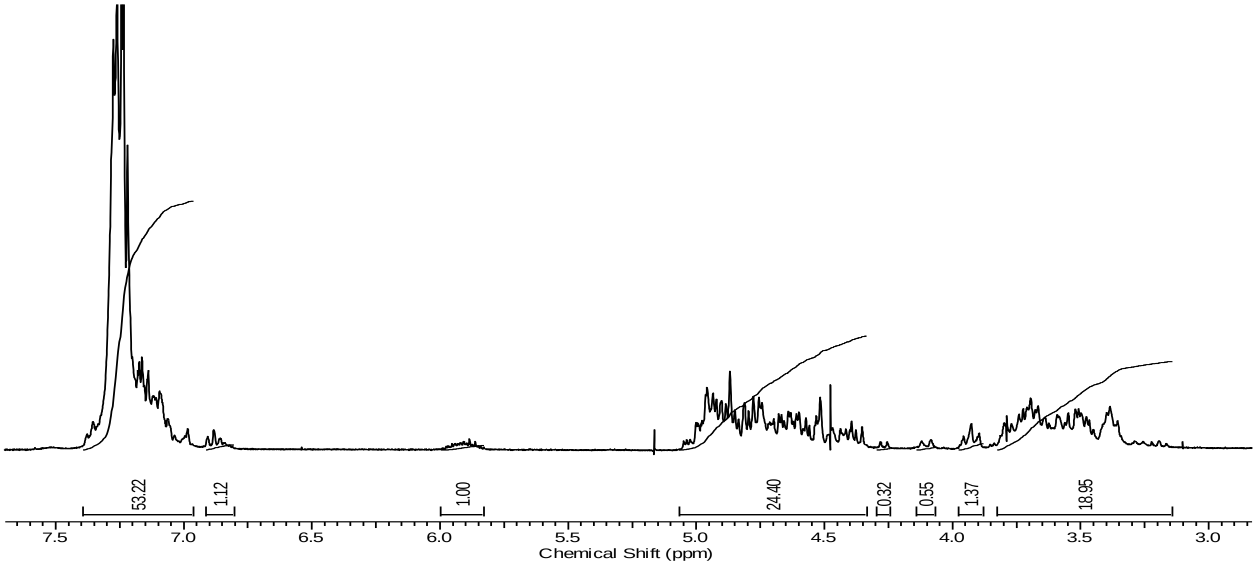

CDCl$_3$ at 300 MHz

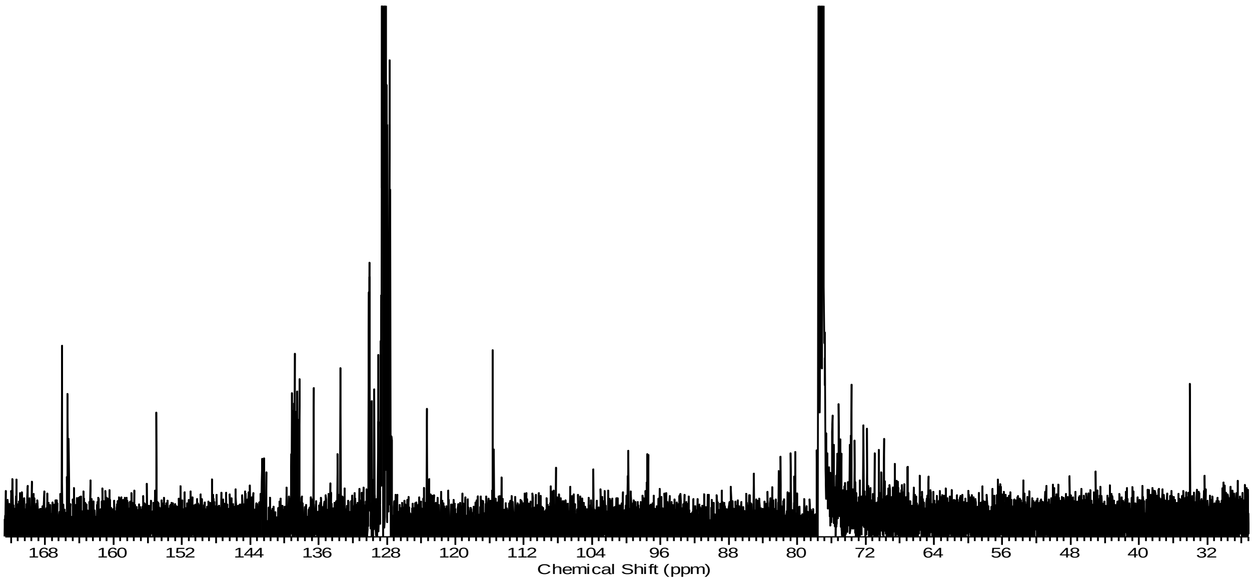

CDCl$_3$ at 75 MHz

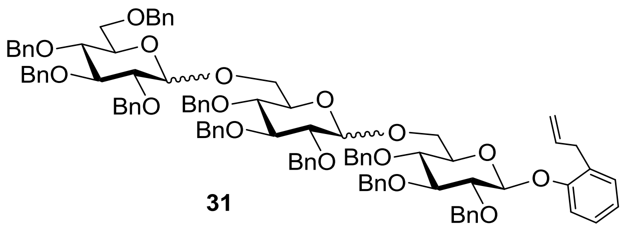

31

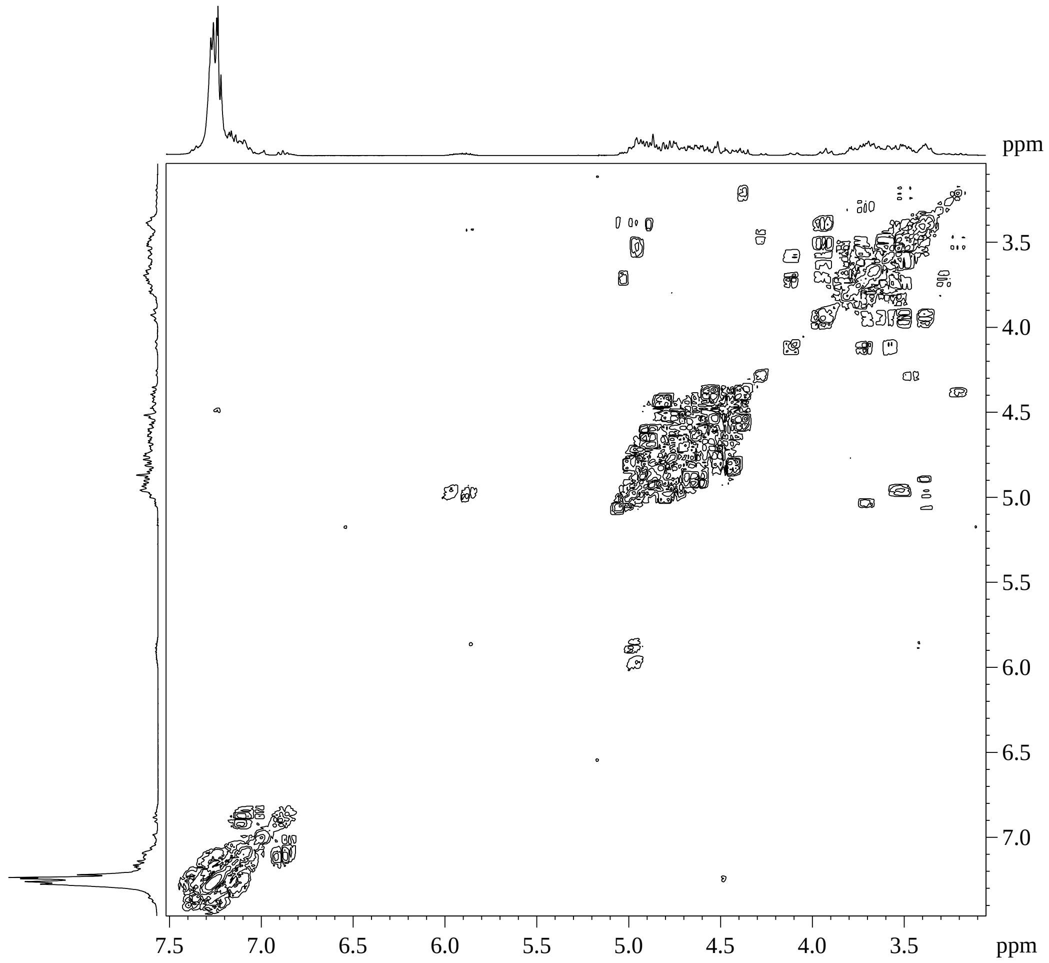

CDCl$_3$ at 300 MHz

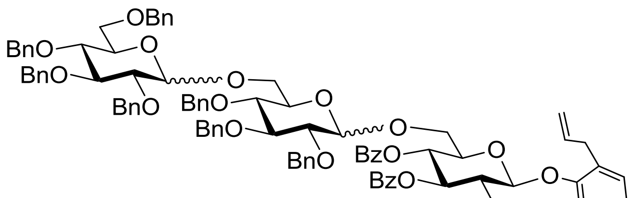

32

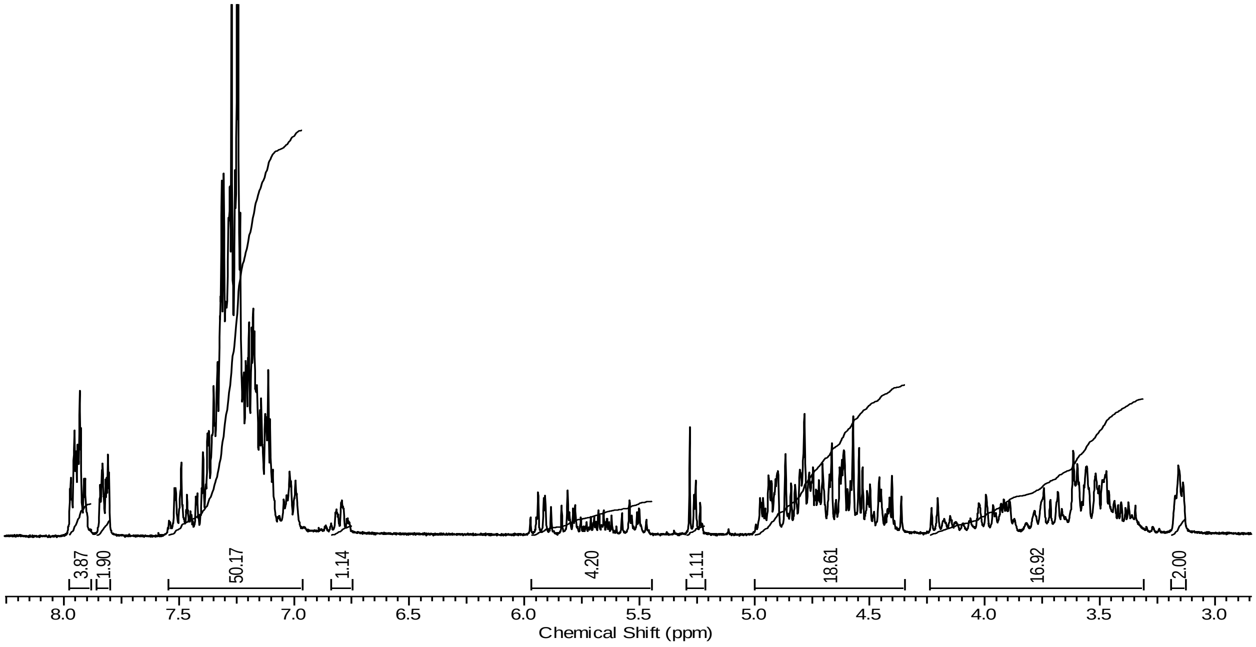

CDCl$_3$ at 300 MHz

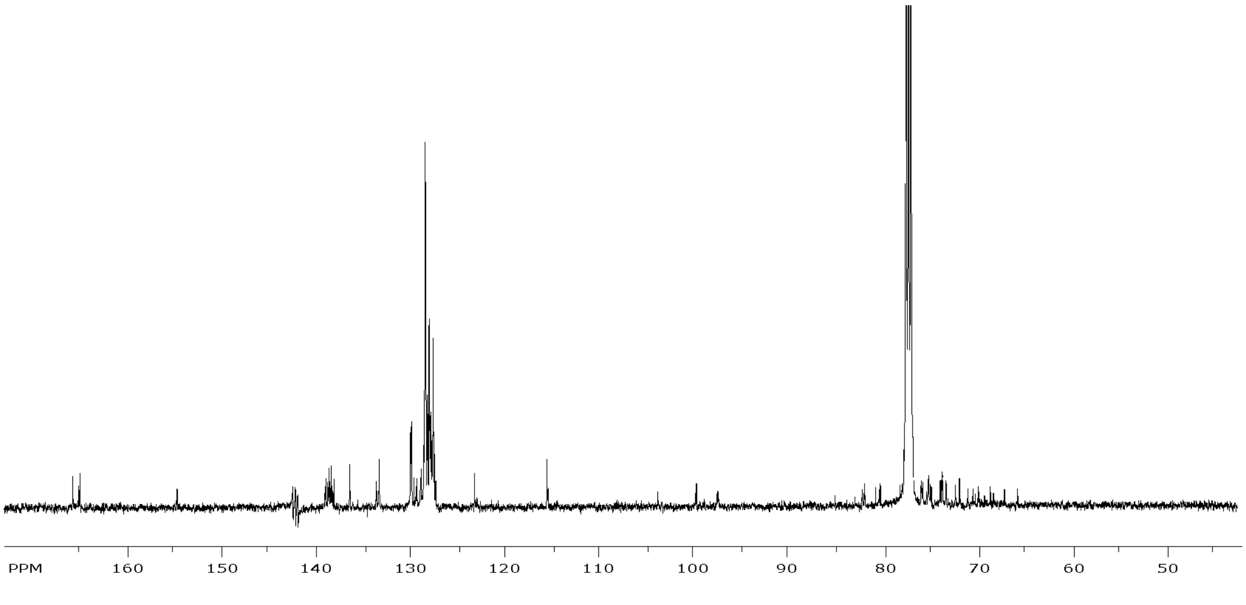

CDCl$_3$ at 75 MHz

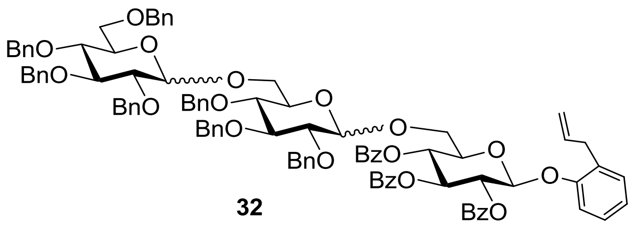

32

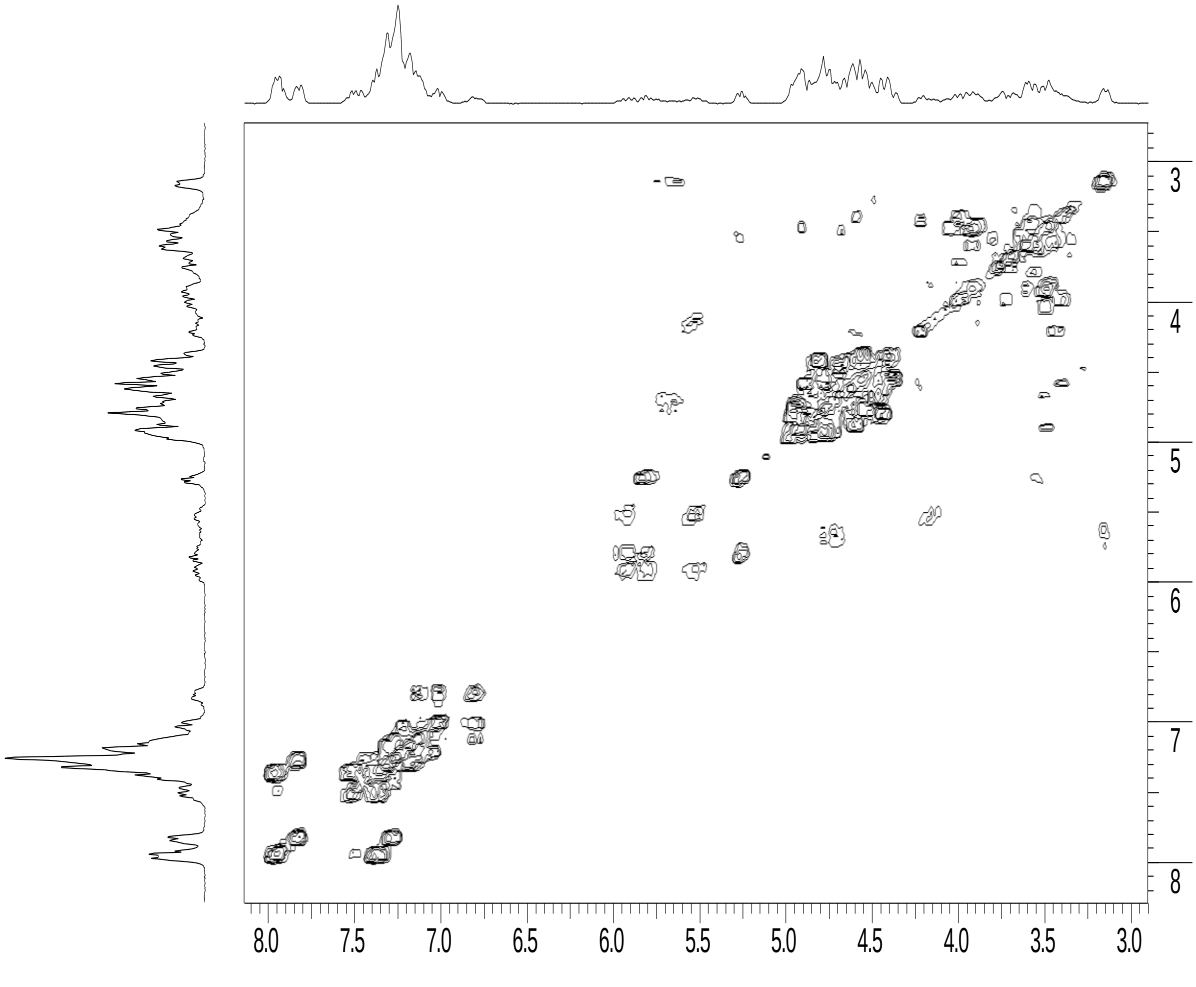

$CDCl_3$ at 300 MHz

References

[1] G. Ekborg and C. P. J. Glaudemans, *Carbohydr. Res.* **1984**, *129*, 287-292.

[2] S. Kaeothip, P. Pornsuriyasak and A. V. Demchenko, *Tetrahedron Lett.* **2008**, *49*, 1542-1545.

[3] A. V. Demchenko, P. Pornsuriyasak, C. De Meo and N. N. Malysheva, *Angew. Chem. Int. Ed.* **2004**, *43*, 3069-3072.

[4] G. Balavoine, S. Berteina, A. Gref, J. C. Fischer and A. Lubineau, *J. Carbohydr. Chem.* **1995**, *14*, 1217-1236.

[5] J. Dinkelaar, A. R. de Jong, R. van Meer, M. Somers, G. Lodder, H. S. Overkleeft, J. D. C. Codee and G. A. van der Marel, *J. Org. Chem.* **2009**, *74*, 4982-4991.

[6] J. T. Smoot, P. Pornsuriyasak and A. V. Demchenko, *Angew. Chem. Int. Ed.* **2005**, *44*, 7123-7126.

[7] J. M. Kuester and I. Dyong, *Justus Liebigs Ann. Chem.* **1975**, 2179-2189.

[8] S. C. Ranade, S. Kaeothip and A. V. Demchenko, *Org. Lett.* **2010**, *12*, 5628-5631.

[9] C. Fei and T. H. Chan, *Acta Chimica Sinica, Engl. Edit.* **1989**, 258-264.

[10] X. Huang, L. Huang, H. Wang and X. S. Ye, *Angew. Chem. Int. Ed.* **2004**, *43*, 5221-5224.

[11] P. J. Pfaeffli, S. H. Hixson and L. Anderson, *Carbohydr. Res.* **1972**, *23*, 195-206.

[12] L. A. J. M. Sliedregt, G. A. van der Marel and J. H. van Boom, *Tetrahedron Lett.* **1994**, *35*, 4015-4018.

[13] B. A. Garcia and D. Y. Gin, *J. Am. Chem. Soc.* **2000**, *122*, 4269-4279.

[14] H. M. Nguyen, Y. N. Chen, S. G. Duron and D. Y. Gin, *J. Am. Chem. Soc.* **2001**, *123*, 8766-8772.

[15] Y. D. Vankar, P. S. Vankar, M. Behrendt and R. R. Schmidt, *Tetrahedron* **1991**, *47*, 9985-9992.

[16] B. Wegmann and R. R. Schmidt, *J. Carbohydr. Chem.* **1987**, *6*, 357-375.

[17] H. Chiba, S. Funasaka and T. Mukaiyama, *Bull. Chem. Soc. Jpn.* **2003**, *76*, 1629-1644.

[18] Y. Kobashi and T. Mukaiyama, *Bull. Chem. Soc. Jpn.* **2005**, *78*, 910-916.

[19] P. Pornsuriyasak and A. V. Demchenko, *Chem. Eur. J.* **2006**, *12*, 6630-6646.

[20] S. Kaeothip, P. Pornsuriyasak, N. P. Rath and A. V. Demchenko, *Org. Lett.* **2009**, *11*, 799-802.
